# An agent-based model of dengue virus transmission shows how uncertainty about breakthrough infections influences vaccination impact projections

**DOI:** 10.1371/journal.pcbi.1006710

**Published:** 2019-03-20

**Authors:** T. Alex Perkins, Robert C. Reiner, Guido España, Quirine A. ten Bosch, Amit Verma, Kelly A. Liebman, Valerie A. Paz-Soldan, John P. Elder, Amy C. Morrison, Steven T. Stoddard, Uriel Kitron, Gonzalo M. Vazquez-Prokopec, Thomas W. Scott, David L. Smith

**Affiliations:** 1 Department of Biological Sciences and Eck Institute for Global Health, University of Notre Dame, Notre Dame, IN, United States of America; 2 Fogarty International Center, National Institutes of Health, Bethesda, MD, United States of America; 3 Department of Epidemiology and Biostatistics, Indiana University, Bloomington, IN, United States of America; 4 Department of Biostatistics and Bioinformatics, Emory University, Atlanta, GA; 5 Department of Entomology and Nematology, University of California, Davis, CA, United States of America; 6 Department of Global Community Health and Behavioral Sciences, Tulane University School of Public Health and Tropical Medicine, New Orleans, LA, United States of America; 7 Institute for Behavioral and Community Health, Graduate School of Public Health, San Diego State University, San Diego, CA, United States of America; 8 Department of Environmental Sciences, Emory University, Atlanta, GA, United States of America; 9 Institute for Health Metrics and Evaluation, University of Washington, Seattle, WA, United States of America; CNRS, FRANCE

## Abstract

Prophylactic vaccination is a powerful tool for reducing the burden of infectious diseases, due to a combination of direct protection of vaccinees and indirect protection of others via herd immunity. Computational models play an important role in devising strategies for vaccination by making projections of its impacts on public health. Such projections are subject to uncertainty about numerous factors, however. For example, many vaccine efficacy trials focus on measuring protection against disease rather than protection against infection, leaving the extent of breakthrough infections (i.e., disease ameliorated but infection unimpeded) among vaccinees unknown. Our goal in this study was to quantify the extent to which uncertainty about breakthrough infections results in uncertainty about vaccination impact, with a focus on vaccines for dengue. To realistically account for the many forms of heterogeneity in dengue virus (DENV) transmission, which could have implications for the dynamics of indirect protection, we used a stochastic, agent-based model for DENV transmission informed by more than a decade of empirical studies in the city of Iquitos, Peru. Following 20 years of routine vaccination of nine-year-old children at 80% coverage, projections of the proportion of disease episodes averted varied by a factor of 1.76 (95% CI: 1.54–2.06) across the range of uncertainty about breakthrough infections. This was equivalent to the range of vaccination impact projected across a range of uncertainty about vaccine efficacy of 0.268 (95% CI: 0.210–0.329). Until uncertainty about breakthrough infections can be addressed empirically, our results demonstrate the importance of accounting for it in models of vaccination impact.

## Author summary

Vaccines are vital tools for safeguarding public health from a variety of infectious disease threats. When decisions are being made about investments in vaccination, computational models provide decision makers with projections of the benefits of vaccination. There are many types of uncertainty that can affect these projections, such as statistical uncertainty about the extent to which vaccination reduces one’s risk of experiencing disease. While this type of uncertainty is well accounted for by vaccine trials, a different type of uncertainty often is not: whether the vaccine blocks infection altogether or simply reduces the severity of disease symptoms. In the case of the latter, “breakthrough infections” occur, meaning that those who are vaccinated are protected but those who are not receive little or no indirect benefit from herd immunity. Focusing on a newly licensed vaccine for dengue, we developed and applied a new simulation model of dengue virus transmission to assess the extent to which uncertainty about breakthrough infections contributes to uncertainty about vaccination impact. We found that a vaccine that prevents breakthrough infections is capable of nearly doubling the impact of vaccination as compared to a vaccine that confers protection solely by reducing the severity of disease symptoms.

## Introduction

Computational models have much to contribute to the advancement of vaccines as tools for public health benefit. These contributions range from aiding the design and interpretation of vaccine trials [1] to projecting the impact of vaccination policies on public health [2]. Projecting impact has been a major focus of modeling efforts over several decades [3], with applications to a wide range of vaccine-preventable diseases. A challenge common to all of these projections is accounting for the many forms of uncertainty that are relevant to vaccination impact. These can include alternative scenarios for how vaccination could be targeted [4,5], unknown aspects of the pathogen’s natural history [6,7], and uncertainty about the vaccine’s profile [8,9].

Uncertainty about a vaccine’s profile is something that all projections of vaccination impact must confront. At a minimum, a vaccine’s profile is characterized by the relative risk, RR, of some outcome in vaccinees as compared to unvaccinated people. This quantity is related to vaccine efficacy, VE, as VE = 1-RR. Other aspects of a vaccine’s profile can include whether protection is “leaky” (reduces per-event risk uniformly for all) or “all or none” (reduces risk fully, but only for some), or whether protection wanes over time or depends on an individual’s age or other characteristics. Uncertainty about these and other aspects of a vaccine’s profile can be addressed through sensitivity analysis [10,11] or by fitting a model to vaccine trial data [12,13]. By either approach, uncertainty about a vaccine’s profile can be propagated into uncertainty about vaccination impact.

One vaccine with a complex profile for which impact projections [14] have played an important role in shaping recent policy decisions [15] is CYD-TDV (brand name Dengvaxia, by Sanofi-Pasteur). This vaccine was developed to protect against dengue, a major viral disease of humans caused by any of four serotypes of dengue virus (DENV) and transmitted among humans by *Aedes aegypti* mosquitoes. Following a series of efficacy trials [16], VE estimates for CYD-TDV were significantly lower for children under nine years of age (0.45) than for children nine years of age or older (0.66). VE estimates were also greater for individuals with prior, natural exposure to DENV (referred to as seropositive), especially for children under nine (seropositive: 0.70, seronegative: 0.14). Additionally, VE estimates varied by serotype, ranging 0.34–0.62 in children under nine. Assumptions about vaccine profile used in projections of CYD-TDV vaccination impact to date have focused primarily on reconciling these age and serotype differences in VE [13,17].

One aspect of CYD-TDV profile that has not been explored in impact projections concerns the clinical nature of trial endpoints. Specifically, the primary endpoint for efficacy trials of CYD-TDV was virologically confirmed dengue among trial participants who experienced acute febrile illness; i.e., a fever of ≥38°C for at least two consecutive days [16]. Among trial participants for whom acute febrile illness was averted due to vaccination, the vaccine could have either blocked DENV infection altogether or ameliorated symptoms but still allowed infection [18]. If we denote the relative risk of infection conditional on exposure as RR_inf|exp_ and disease conditional on infection as RR_dis|inf_, it follows that relative risk of the disease endpoint is RR_dis_ = RR_dis|inf_ x RR_inf|exp_. Without measuring a secondary endpoint related to infection, RR_inf|exp_ cannot be estimated and we are left knowing only the product RR_dis_.

The distinction between RR_dis|inf_ and RR_inf|exp_ is an important one, because people with clinically inapparent DENV infections have been shown to transmit DENV to mosquitoes [19] and have been estimated to contribute appreciably to transmission [20]. This raises the possibility of breakthrough DENV infections among CYD-TDV vaccinees, particularly if RR_inf|exp_ is large. Vaccines that prevent breakthrough infections can have a substantial impact on public health outcomes [21], due to the fact that they provide both direct protection of vaccinees and indirect protection of others. Indirect protection derives from a population-level phenomenon known as herd immunity [22], projections of which require assumptions about population-level transmission dynamics. To the extent that there is uncertainty about breakthrough infections among vaccinees, there will inevitably be uncertainty about the extent of indirect protection of those who go unvaccinated [23].

Our primary interest here was in assessing the extent of uncertainty in CYD-TDV impact projections attributable to uncertainty about breakthrough infections. The effect of breakthrough infections on vaccination impact is a function of the extent to which they erode indirect protection, which depends in part on the nature of contact between vaccinated and unvaccinated people and on the structure of transmission more generally [24,25]. To obtain a realistic portrayal of the structure of transmission in an endemic setting, we used an agent-based simulation model of DENV transmission developed and calibrated for the city of Iquitos, Peru, which has had ongoing studies of dengue epidemiology for more than a decade [26,27]. We simulated DENV transmission in the presence and absence of routine vaccination across a range of assumptions about breakthrough infections. To place these results into context, we compared them to results from simulations with varying values of VE under two different models of dengue vaccine profile.

## Methods

## Model overview

**Fig 1:**
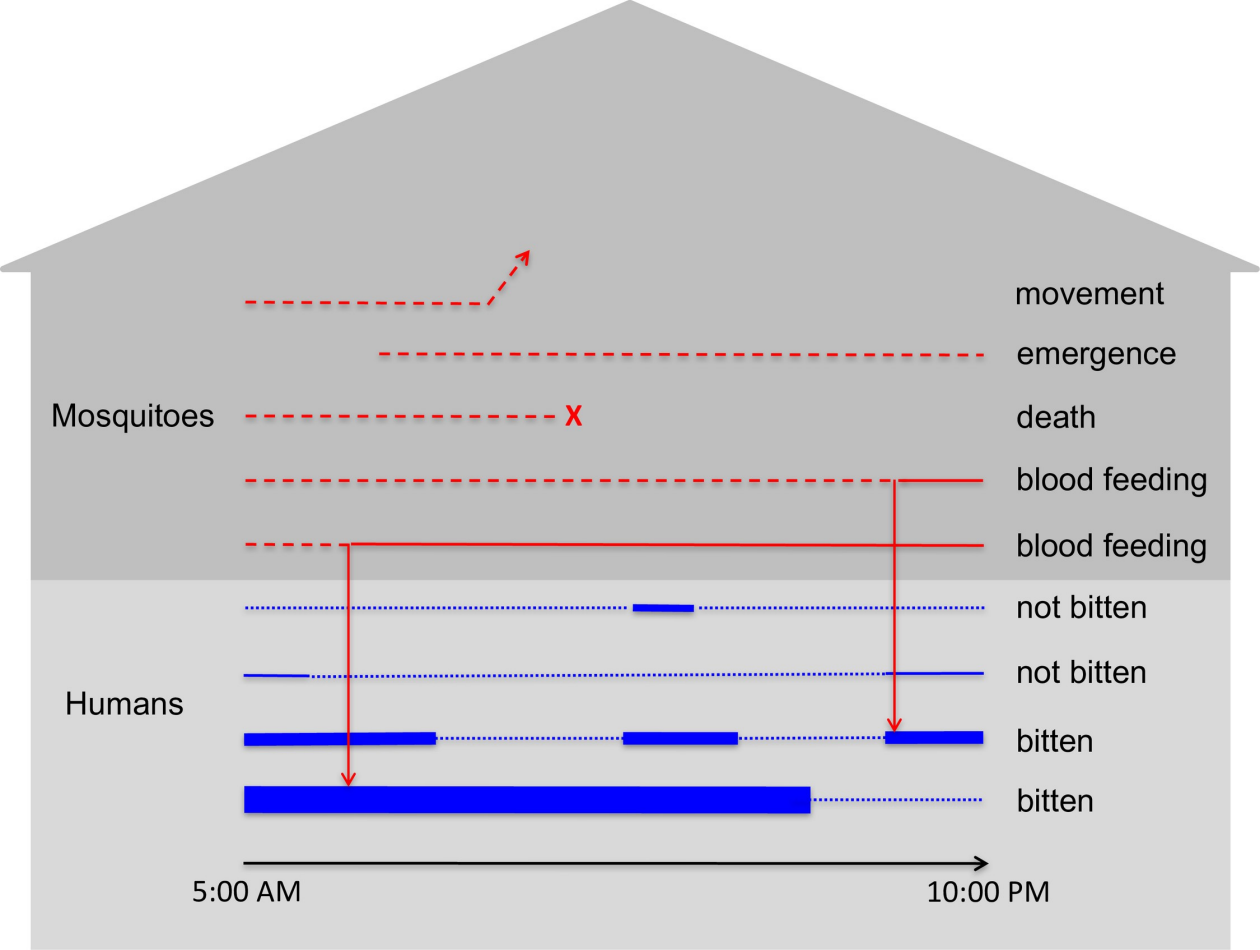
Example of events that occur at the individual level (lines) at a single location (gray house shape) over the course of a single day. Red lines correspond to individual mosquitoes, with dashed and solid lines representing host-seeking and resting states, respectively. Blue lines refer to individual people, with thin dotted lines indicating that the person is at another location at that time and thick solid lines indicating their presence at the location at that time. The thickness of the solid blue lines indicates the relative attractiveness of each person to blood feeding by mosquitoes.

We developed a stochastic, agent-based model for simulating DENV transmission that is parameterized in a number of respects around studies of dengue epidemiology conducted in Iquitos, Peru. The model simulates DENV transmission in a population of approximately 200,000 people residing in the core of Iquitos, which consists of 38,835 geo-referenced houses and 2,004 other buildings [28]. Events such as mosquito biting, mosquito death, and movements by humans and mosquitoes are scheduled to occur at continuous time points throughout the day (Fig 1), with updating of individuals’ statuses with respect to infection, immunity, and demographics occurring once daily. The only abiotic factor incorporated into the model explicitly was temperature, which influenced several time-varying parameters and was informed by daily mean temperature recordings from a weather station at the Iquitos International Airport. We describe the model in full detail in S1 Text, following the ODD (Overview, Design concepts, Details) Protocol [29,30] for describing agent-based models. In the paragraphs below, we provide an overview of key features of the model pertaining to humans, mosquitoes, and viruses.

Humans are populated in the city consistent with national age and sex distributions for Peru and in individual houses consistent with demographic data collected over the course of studies in Iquitos. Birth and death processes are parameterized consistent with demographic estimates and future projections by the United Nations [31], with age- and year-specific death rates, year-specific population birth rates, and age- and year-specific relative fertility among females aged 15–49. Aging involves the acquisition of lifelong, serotype-specific immunity as each person is exposed, and sex-specific growth of an individual’s body size over the course of childhood to allow for an effect of body size on propensity to be bitten by mosquitoes [32]. Each individual human possesses a unique “activity space,” which is defined as an average pattern of time allocation across all the locations that they frequent between 05:00 and 22:00, when risk of biting from *Ae*. *aegypti* mosquitoes is expected to be highest [33]. Individuals move about this activity space in a manner based on retrospective interviews performed on residents of Iquitos and modeled in a way described previously by Perkins et al. [34].

The number of adult female mosquitoes in the area is determined by a combination of mosquito emergence and death processes. Mosquito death occurs according to unique daily, temperature-dependent rates derived from Brady et al. [35]. Mosquito emergence occurs differentially by location according to unique daily emergence rates that were estimated by determining what emergence patterns, when combined with the death process in our model, would yield spatiotemporal patterns of mosquito density consistent with statistical estimates by Reiner et al. [36]. In addition to emergence and death, mosquitoes move from their current location to a nearby location on any given day with a fixed probability [37]. They engage in biting at temperature-dependent rates that differ depending on whether it is the mosquito’s first bite [38] or a subsequent bite [39]. They select an individual on whom to blood feed based on the body size of each person present at a location at the time that a mosquito bites [32,34]. Because the emphasis of the present analysis is on vaccination rather than vector control, we deferred the inclusion of additional entomological details for future work.

The model allows for the transmission of all four DENV serotypes, which are assumed to be identical in the following respects based on empirical studies: infectiousness [19,40], intrinsic and temperature-dependent extrinsic incubation periods [41], and rate of symptomatic disease [20,42]. For infectiousness of mosquitoes to people, we adopted a value used by another modeling study (0.9, [43]), given the difficulty of estimating this parameter empirically. For infectiousness of people to mosquitoes, we used a time-varying function of infectiousness based on empirical data [44], which peaks on the day of symptom onset and lasts for a total of five days. Individual people can experience up to four distinct infections over the course of their lifetimes, as they experience lifelong immunity to each serotype to which they have been exposed and temporary cross-immunity to all serotypes following exposure [45]. The probability that an individual infected with DENV develops symptomatic disease depends on whether the infection is primary (0.18), secondary (0.24), or post-secondary (0.14) [20,42].

To seed transmission, viruses of each serotype are introduced into the population at a time-varying rate through infected people that are each simulated to have an activity space identical to a randomly chosen resident for the duration of their infection. Because currently available data in the geographic information system only permits us to simulate approximately half the population of the metropolitan area, we viewed these infections in temporary individuals primarily as representative of people from other parts of the city moving DENV into the population represented explicitly in our model. Were we to model the entire population of Iquitos, we expect that fewer such infections would be necessary to seed transmission within our synthetic population. In addition, although Iquitos is relatively isolated in general, some limited introduction of DENV is known to occur from surrounding areas [46].

### Model calibration

Wherever possible, we parameterized the model directly based on empirical estimates from Iquitos or from studies conducted elsewhere and reported in the literature. This included human demography [31,32], human mobility [34], human-mosquito encounters [32], mosquito abundance patterns in space and time [36,47,48], mosquito movement [37], mosquito mortality [35], mosquito blood-feeding rates [38,39], virus incubation in mosquitoes and humans [41], infectiousness of humans to mosquitoes [44], and naturally acquired immunity to DENV [45].

Two primary uncertainties that we were not able to quantify a priori were DENV importation into Iquitos and the scaling relationship between household mosquito surveys and true mosquito abundance. We calibrated those parameters such that simulated model behavior was consistent with empirical estimates of time-varying, serotype-specific patterns of incidence of DENV infection [49]. These empirical estimates to which our model was calibrated were based on interval-censored, serotype-specific seroconversions obtained through longitudinal cohort studies conducted over a period of 11 years [49]. The basis of our calibrations was maximization of the goodness of fit of simulated incidence *I*_*s*,*t*_ of serotype *s* at time *t* to probabilistic estimates of *I*_*s*,*t*_ by Reiner et al. [49]. For each month between January, 2000 and June, 2010, we first performed maximum-likelihood fitting of a Dirichlet distribution to 1,000 draws of the serotype proportions of *I*_*s*,*t*_ and a normal distribution to 1,000 draws of the total incidence *I*_*t*_ at time *t* from the posterior distribution estimated by Reiner et al [49]. We then used the product of the probability densities of those distributions evaluated at the simulated values of *I*_*s*,*t*_ for each serotype as our measure of goodness of fit.

Using this measure of goodness of fit, we obtained estimates of unknown parameters for DENV importation and scaling of mosquito abundance using a particle filtering algorithm. The premise of this algorithm is to make use of the fact that most of the unknown parameters pertain to only a portion of the time series—and thereby only a portion of the likelihood—to allow for calibrating different subsets of the unknown parameters sequentially rather than simultaneously. There are a wide range of particle filtering algorithms, but ours most closely resembles a sequential importance resampling algorithm [50].

The first step in our algorithm involved proposing a set of 1,000 initial particles spanning a range of parameter values, simulating the first year of the model for each particle, evaluating the goodness of fit measure described above on a monthly basis within the first year, and combining the monthly goodness of fit measures to obtain an annual goodness of fit measure for each particle for the first year. Next, we resampled the particles 1,000 times with replacement weighted by

$${\text{weight}}_{i}\;=\;\frac{\exp\left(-c\;LL_{i}\right)}{\sum_{i}\exp\left(-c\;LL_{i}\right)}$$ (1)

for each particle *i*, where *c* is a scaling parameter that we tuned to a value of 0.1 to result in resampled particles containing at least 10% of the original particles. We then obtained maximum-likelihood estimates of the means and covariance matrix describing the distribution of the particles. Using that fitted multivariate normal distribution, we then drew 1,000 new particles and simulated both the first and second year of transmission. We then computed the goodness of fit measure for the first two years by aggregating monthly goodness of fit measures across the first two years. Additional steps in the algorithm were repeated in the same way in yearly increments through the last year for which empirical estimates of time-varying, serotype-specific incidence were available. Finally, we performed two additional rounds of resampling on the full time series following the last year of simulation and particle resampling. The resulting set of 1,000 particles constituted our distributional estimate of parameter values most consistent with available empirical estimates.

### Vaccine profile

#### CYD-TDV vaccine

The mode of action of CYD-TDV is not completely clear, given that there are multiple mechanisms by which results from clinical trials could have come about [51]. We modeled VE against the primary trial endpoint of virus-confirmed disease as a function of age and serostatus, which is consistent with one hypothesis for how the vaccine achieves its efficacy [16,51] but differs from others [13]. Specifically, for a given serostatus, we modeled the relationship between age and VE against disease as

$${\text{VE}}_{\text{dis}}\left(\text{age}\right)=\;1\;-\;\frac{c_{1}}{1+\exp\left(\;c_{2}\;\left(\text{age}\;-\;c_{3}\right)\right)}$$ (2)

using values of *c*_1_, *c*_2_, and *c*_3_ fitted separately to data from groups that were seropositive or seronegative at baseline. This functional form was chosen based on the fact that its shape is relatively flexible and that it yields a monotonic relationship between age and VE that allows for VE to assume negative values at young ages (increase in risk of endpoint) and to approach 1 (full protection) at older ages, consistent with assumptions about the profile of CYD-TDV [51]. To obtain point estimates of *c*_1_, *c*_2_, and *c*_3_ for both serostatus groups, we fitted Eq (2) under different values of these parameters to mean estimates of VE for seropositive and seronegative 2–9 and 10–16 year-olds reported in Fig 2 of Hadinegoro et al. [16]. Fitting was performed on the basis of least squares using the Nelder-Mead algorithm as implemented in the optim function in R [52]. These calculations assumed an even age distribution within each age class in the trials.

**Fig 2:**
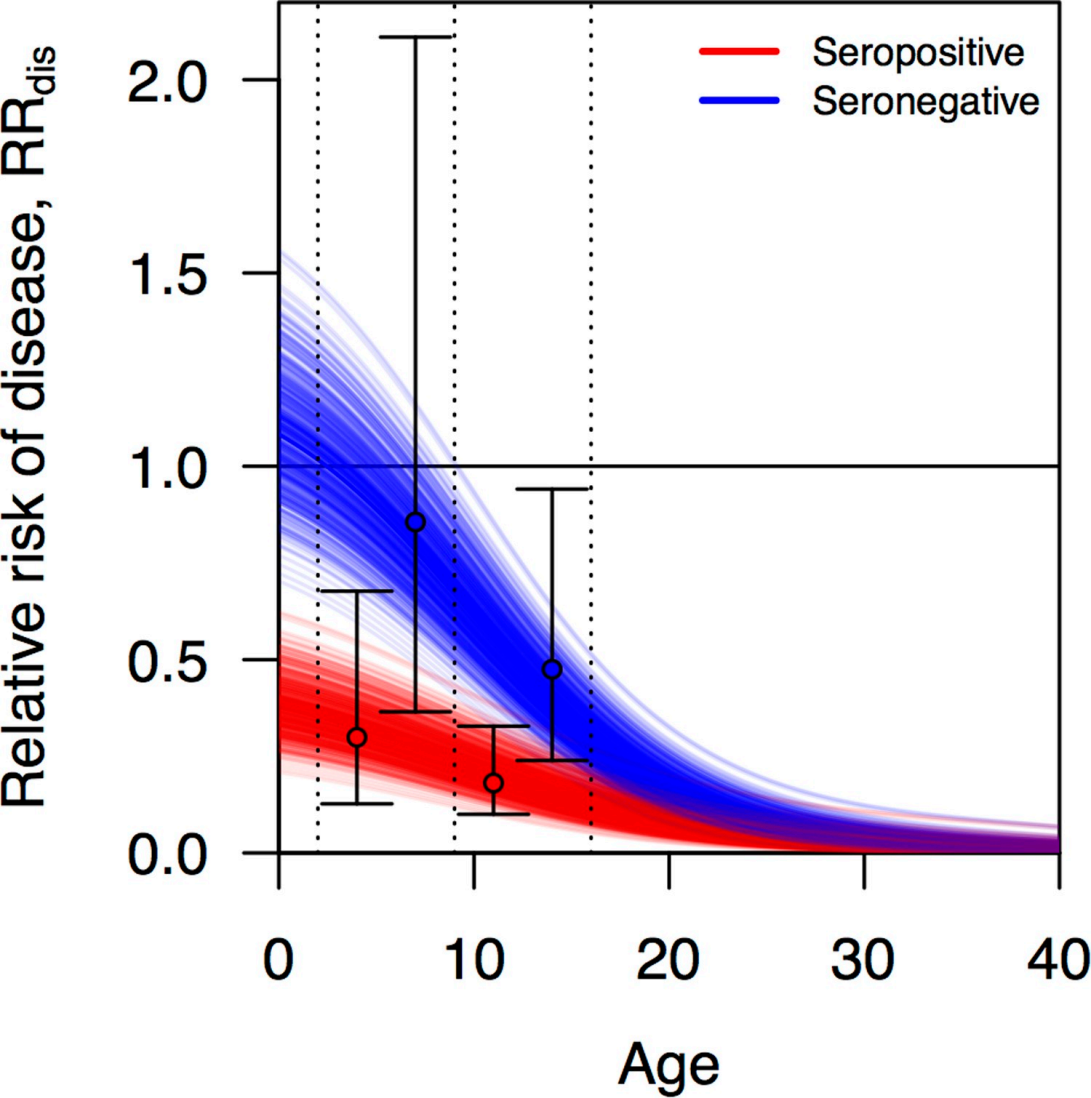
Relative risk of disease, RR_dis_, as a function of age and serostatus (blue: seronegative, red: seropositive) estimated from vaccine trial data [16]. Each line represents a distinct random draw from the distribution of these relationships. Black circles correspond to point estimates of relative risk of disease in the trial for a given age group (2–9 left, 9–16 right) and serostatus (red vs. blue), and error bars indicate 95% confidence intervals around those estimates.

We modeled statistical uncertainty around estimates of VE with a parameter σ that describes the standard deviation of the log of the relative risk (RR), defined mathematically as

$$\sigma \left(\text{ln}\left({\text{RR}}_{\text{dis}}\right)\right)\;=\;{\left(1/\left(c_{4}+c_{5}\right)+1/\left(c_{4}\left(1-{\text{VE}}_{\text{dis}}\right)+c_{5}\right)\right)}^{\frac{1}{2}}$$ (3)

to yield a one-to-one relationship between VE_dis_ and the standard deviation of the log of RR_dis_. To fit values of *c*_4_ and *c*_5_, we used a method based on the assumption of asymptotic normality of the log of the ratio of Poisson rates [53], applied to standard errors presented in Fig 2 of Hadinegoro et al. [16]. To draw a quantile *q* of VE_dis_ for a given instance of the simulation, we drew the *q* quantile from a normal random variable with mean 0 and standard deviation 1, multiplied it by $\sigma (ln(RR_{dis})),$ added the result to ln(1-VE_dis_), exponentiated the result, and subtracted it from 1 [16]. In all simulations, we applied the same value of *q* to the calculation of VE_dis_ for both seropositive and seronegative vaccine recipients.

#### Generic dengue vaccine

To examine the robustness of our results to assumptions about the profile of CYD-TDV, we also considered a hypothetical and more generic dengue vaccine with a wide range of possible profiles. We characterized this vaccine’s profile with three parameters: VE_mean_, VE_serostatus_, and VE_serotype_. Under this model, an individual’s RR_dis_ depends on their pre-vaccination serostatus and the DENV serotype to which they were exposed, but not their age. To ensure that VE_mean_ does in fact represent a mean, each individual’s VE began there and was adjusted up or down by VE_serostatus_ and VE_serotype_. One half of VE_serostatus_ was always subtracted from VE_mean_ for seronegative individuals and added for seropositives. From there, an increment was added or subtracted such that the four serotype-specific VEs spanned a range of VE_serotype_. Which serotypes had higher or lower VE was randomized across simulations. Following these steps, RR was calculated as 1 less the VE determined by an individual’s serostatus and the infecting serotype.

For example, consider values of VE_mean_ = 0.6, VE_serostatus_ = 0.1, and VE_serotype_ = 0.06. Under these parameters, the average VE would be 0.6, and the average VEs for seropositive and seronegative groups would be 0.65 and 0.55, respectively. For seropositive (seronegative) individuals, VE would be 0.68 (0.58), 0.66 (0.56), 0.64 (0.54), and 0.62 (0.52) against four randomly ordered serotypes. Although we selected specific values of these parameters here to illustrate how these calculations work, these values are only illustrative. We intentionally chose a wide range of values of each parameter to consider in our analysis.

#### Protection against breakthrough infections

Due to the relationship RR_dis_ = RR_dis|inf_ x RR_inf|exp_, a vaccine could derive its efficacy against disease solely through amelioration of symptoms (RR_dis|inf_ = RR_dis_), solely through blocking breakthrough infections (RR_inf|exp_ = RR_dis_), or from some combination of RR_dis|inf_ and RR_inf|exp_ along a continuum between these two extremes. To fully explore that continuum, we define a parameter *p*, which quantifies the relationship among these three different versions of RR as RR_inf|exp_ = RR_dis_^*p*^ and RR_dis|inf_ = RR_dis_^1-*p*^. Constraining values of *p* to range between 0 and 1, this guarantees that the definitional relationship among these RR variables is followed. The extreme of *p* = 1 represents complete blocking of breakthrough infection, and the extreme of *p* = 0 represents protection deriving only from amelioration of symptoms. In all simulations, we assumed that the vaccine is leaky, meaning that an individual has some chance of becoming infected each time they are exposed and has some chance of developing disease each time they are infected. Definitions of parameters related to vaccine profile are summarized in Table 1.

**Table 1:** Definitions of key terms.

Term	Symbol	Definition
Relative risk of disease conditional on infection	RR_dis|inf_	Proportion of vaccine recipients that experience disease after becoming infected relative to the proportion of placebo recipients that experience disease after becoming infected.
Relative risk of infection conditional on exposure	RR_inf|exp_	Proportion of vaccine recipients that become infected after being bitten by an infectious mosquito relative to the proportion of placebo recipients that become infected after being bitten by an infectious mosquito.
Relative risk of disease	RR_dis_	Proportion of vaccine recipients that experience disease after being bitten by an infectious mosquito relative to the proportion of placebo recipients that experience disease after being bitten by an infectious mosquito. This is equal to the product of RR_dis|inf_ and RR_inf|exp_.
Vaccine efficacy against disease	VE_dis_	1—RR_dis_
Proportion of protection against disease derived from protection against infection	*p*	This parameter relates RR_inf|exp_ to RR_dis_ according to the relationship RR_inf|exp_ = RR_dis_^p^. Likewise, it is implied that RR_dis|inf_ = RR_dis_^1-p^ and RR_dis_ = RR_dis|inf_ x RR_inf|exp_.
Quantile of RR_dis_ estimate	*q*	Quantile between 0 and 1 applied to the uncertainty distribution of the RR_dis_ estimate.

### Vaccination impact projections

For each of the two sets of assumptions about vaccine profile, we performed 1,000 pairs of simulations with model parameters drawn from the final set of parameter samples obtained through the calibration process. In both cases, we randomly sampled values of *p* between 0 and 1 for each simulation pair. For the CYD-TDV vaccine, we also sampled values of a parameter *q* that represents the quantile of the RR_dis_ estimates. For the generic dengue vaccine, we sampled values of VE_mean_ of 0.15–0.85, VE_serostatus_ of 0–0.15, and VE_serotype_ of 0–0.15. Values of the latter three parameters were chosen to ensure that the maximum VE could not exceed 1 or fall below 0 and that a broad range of VE_mean_ was covered. Parameter draws were performed with the sobol function in the pomp package [54] in R [52] to maximize the evenness of our coverage of parameter space.

The two simulations in each pair exhibited identical dynamics for the first 11 years, because they were both driven by the same parameter particle and both shared common random number seeds for processes related to mosquito-human contact and DENV infection, respectively. Following that initial time period, we continued one simulation without vaccination but commenced the other with routine vaccination at age nine, both for an additional 20 years. Consistent with other CYD-TDV impact projections [14], we assumed 80% coverage. For each simulation pair, we recorded the following in the population as a whole: proportion of cumulative infections averted and proportion of cumulative disease episodes averted, with both accruing over the period that followed the time period calibrated to Iquitos. Despite taking steps to reduce noise by controlling random number seeds, simulation results were still relatively noisy due to the highly stochastic nature of the model. To distinguish signal from noise when examining relationships between predictor and response variables in these simulation analyses, we fitted generalized additive models (GAMs) to these relationships using the mgcv package [55] in R.

### Sensitivity analysis

To assess the robustness of our conclusions about the effect of breakthrough infections on vaccination impact projections, we performed a series of sensitivity analyses. These analyses repeated the full process of model calibration and vaccination impact projection under alternative assumptions about several parameters: duration of cross-immunity, mosquito infectiousness, human infectiousness, mosquito movement probability, extrinsic incubation period, mosquito death rate, and mosquito biting rate (Table 2). Analyses identical to the primary analysis with default parameter values were performed on each of the sets of vaccination impact projections associated with each of these alternative parameter scenarios. Our primary interest in these sensitivity analyses was assessing the consistency of differences in infections and disease episodes averted across the range of values of parameters describing different aspects of vaccine profile.

**Table 2:** Scenarios examined through sensitivity analysis. As each parameter was varied from its default value, all other parameters were held at their default values. Under each parameter scenario, a separate calibration was performed prior to performing vaccination impact projections.

Parameter	Low	Default	High
Human infectiousness	Asymptomatic infections have ~80% infectiousness [20]	All infections follow the same trajectory of infectiousness [44]	–
Duration of cross-immunity	180 days	686 days [45]	–
Distribution of cross-immunity	Fixed	Exponential	–
Mosquito infectiousness	0.5	1.0	–
Mosquito biting rate	Multiply default by 0.5	Temperature-dependent [38,39]	Multiply default by 2
Mosquito death rate	Multiply default by 0.5	Temperature-dependent [35]	Multiply default by 2
Extrinsic incubation period	Multiply default by 0.5	Temperature-dependent [41]	Multiply default by 2
Mosquito movement probability	0.1	0.3 [37]	0.5

## Results

### Model calibration

Over the course of the calibration process, the marginal distributions of individual parameters narrowed relative to their starting ranges, particularly during three final iterations that made use of the full time series (S1–S9 Figs). Correlations among parameters all started at zero but diversified over the course of successive iterations of the calibration process (S10 Fig), suggesting that effects of some parameters on model performance interacted with effects of others. In general, the strongest correlations tended to be between the e_0_ and e_1_ parameters for scaling mosquito emergence and parameters describing DENV introduction patterns in years with large epidemics. To account for uncertainty associated with correlations among parameter estimates, replicate parameter draws sampling from the final set of parameters (rightmost columns in S1–S9 Figs) were used in all simulations subsequent to calibration.

**Fig 3:**
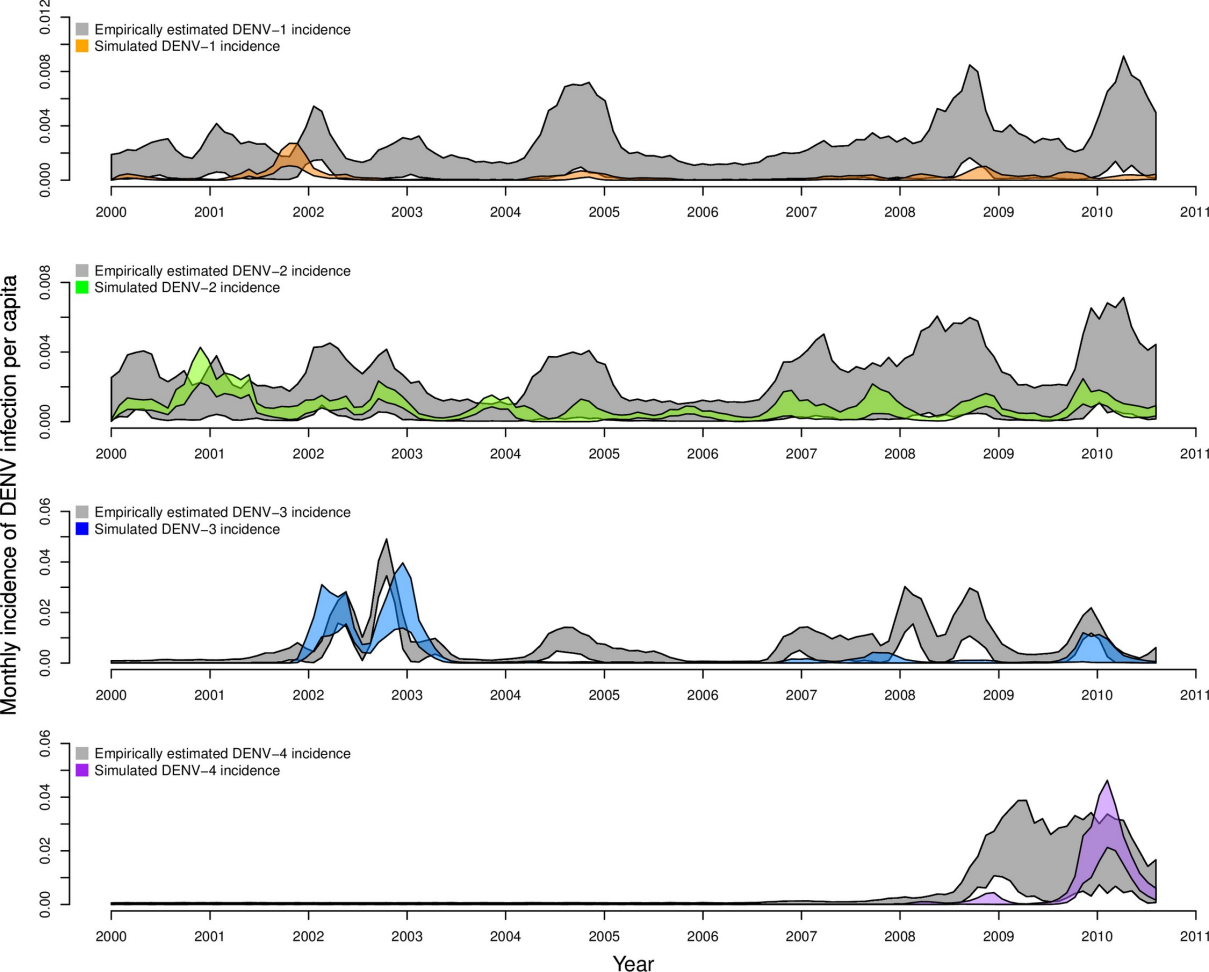
Monthly, serotype-specific incidence of infection per capita, as estimated by Reiner et al. [49] (gray bands) and as reproduced by our calibrated model (colored bands). Bands show the range of values in which 95% of simulated values lie for a given serotype in a given month. These values were obtained under default parameter assumptions detailed in Table 2.

The behavior of the calibrated model (Fig 3, colored bands) was largely in agreement with the estimates of time-varying, serotype-specific incidence of infection to which it was calibrated (Fig 3, gray bands). For all serotypes, the 95% prediction interval of simulations from our agent-based model and estimates from Reiner et al. [49] overlapped for the majority of the 2000–2010 timeframe. Both patterns reflected relatively low and seasonally variable patterns of DENV-1 and DENV-2 transmission, and both captured large seasonal peaks in 2002–2003 for DENV-3 and in 2009–2010 for DENV-4, coinciding with the respective invasions of those serotypes. Note that y-axis ranges span a full order of magnitude (Fig 3: 0.008–0.08) for panels corresponding to different serotypes. These results were largely similar under eleven alternative scenarios about model parameters described in Table 2 (S11–S22 Figs), indicating that the model’s ability to reproduce dynamics from Iquitos was robust to these assumptions and that our algorithm for calibrating the model led to convergent estimates across multiple runs.

**Fig 4:**
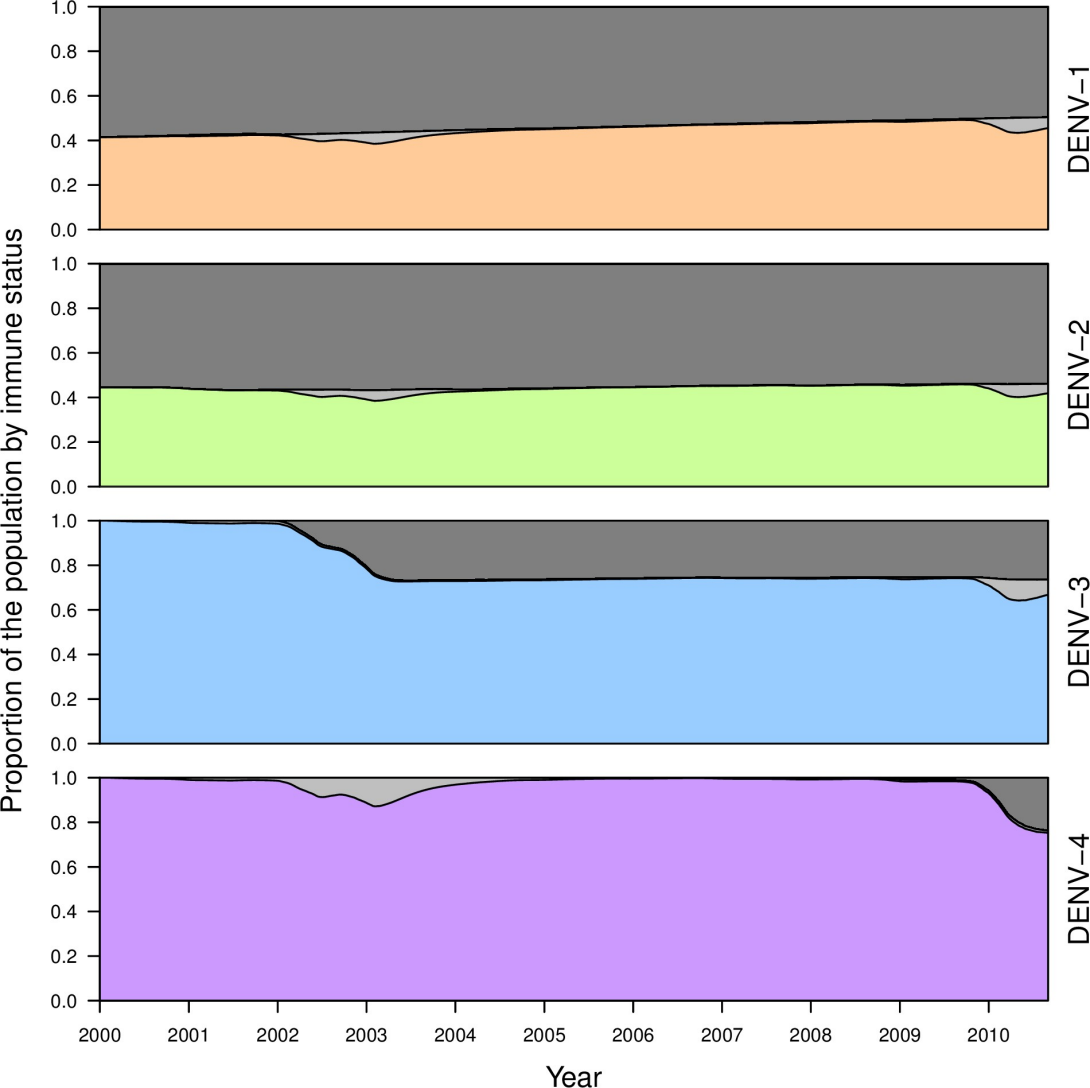
Proportion of the population during the period of the calibration that was not immune to a given serotype (colored), temporarily cross-immune to that serotype (light gray), or permanently immune to that serotype (dark gray). Values shown reflect medians across 1,000 simulations drawing parameters from across the set of particles obtained through the calibration process under default parameter assumptions detailed in Table 2.

**Fig 5:**
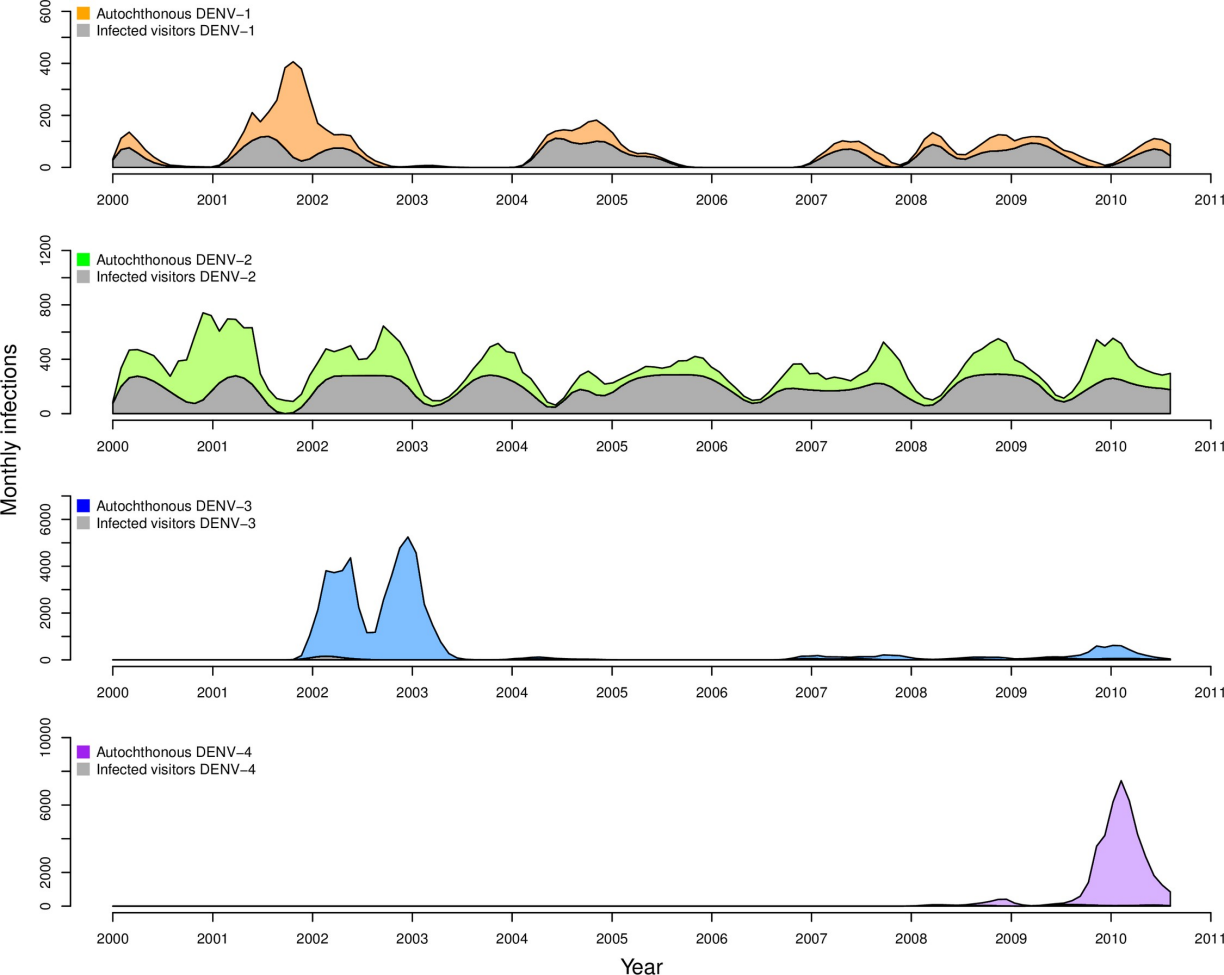
Median numbers of infections on a monthly basis for each serotype, stratified by whether the infection was acquired through biting by an infectious mosquito (colored) or by exogenously driven infections (gray) that were used to seed transmission in the model. These bands represent median values across the set of calibrated parameter values, and the colored bands are added on top of the gray bands. These values were obtained under default parameter assumptions detailed in Table 2.

Although the calibrated model was in relatively good agreement with estimates of incidence patterns by Reiner et al. [49] for large transmission seasons early in the occurrence of a given serotype, the model had a tendency to produce somewhat lower incidence patterns afterwards (Fig 3). These periods of lower incidence were associated with lower population susceptibility to a given serotype (Fig 4) and tended to require a larger number of infections to seed transmission (Fig 5). The relatively low number of infections required to seed the larger epidemics was encouraging with regard to the model’s ability to reproduce large epidemics in Iquitos on the basis of the model’s characterization of local transmission processes. The relatively high number of infections required to seed inter-epidemic transmission is likely a result of the limited population size of 200,000 and the highly stochastic nature of transmission at times of low incidence.

### Vaccine profile

**Fig 6:**
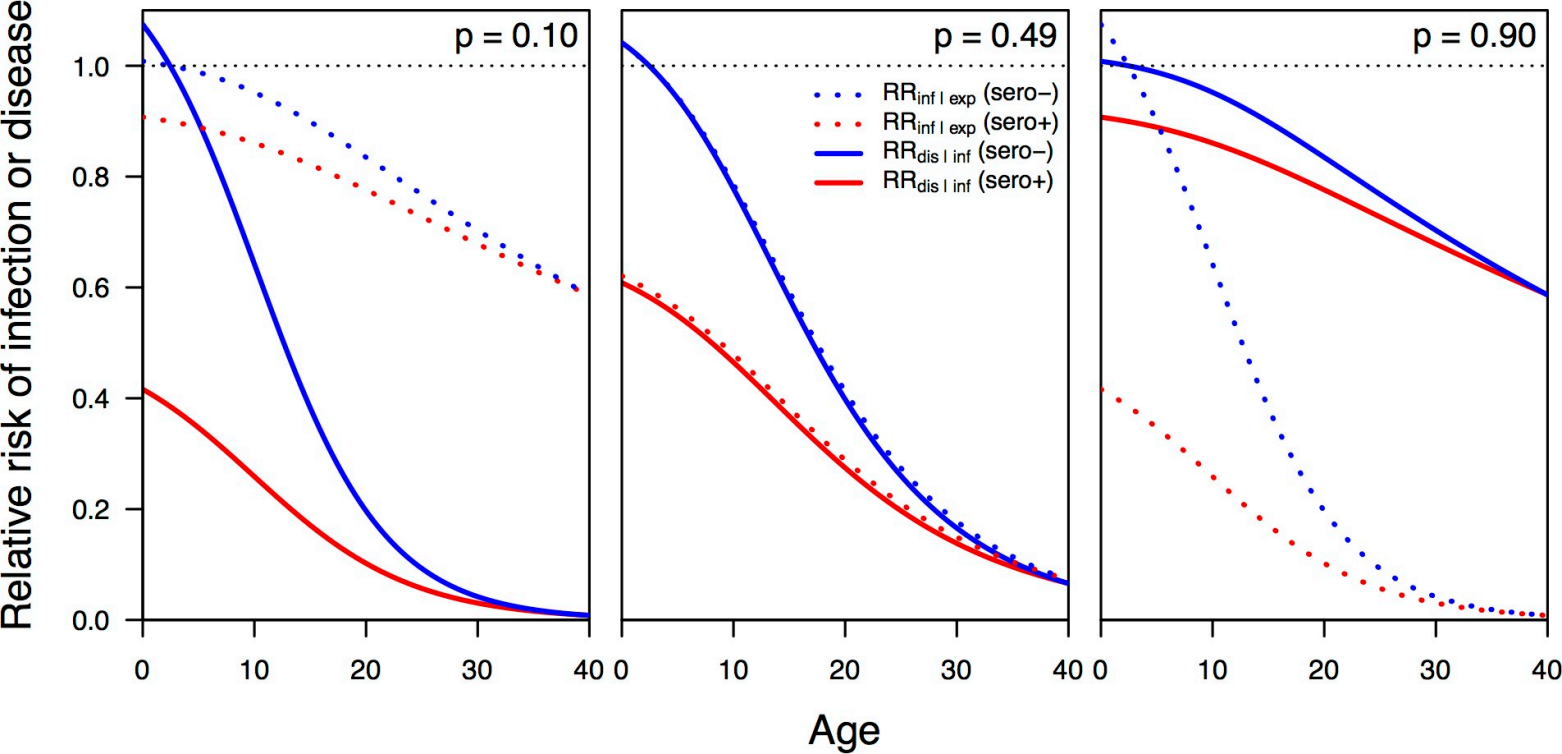
Relative risk of infection conditional on exposure (dashed) and of disease conditional on infection (solid) for seropositive (red) and seronegative (blue) individuals of different ages. These relationships are shown for three different values of the parameter p that specifies the proportion of the overall efficacy against disease that is attributable to protection against infection conditional on exposure.

We obtained estimates of the parameters for relative risk in Eq (2) that best matched empirical estimates [16] of *c*_1_ = 0.47, *c*_2_ = 0.148, and *c*_3_ = 9.17 for seropositive vaccine recipients and *c*_1_ = 1.26, *c*_2_ = 0.28, and *c*_3_ = 9.27 for seronegative vaccine recipients. We obtained estimates of the parameters determining the standard error of the log of the risk ratio in Eq (3) of *c*_4_ = 100 and *c*_5_ = 0.5. Under this model and with these parameters, relative risk decreased steeply with age until around age 20, when it began to decrease more slowly towards almost no risk in older people (Fig 6). As in the CYD-TDV trial data, relative risk under our model was several fold lower in seropositive than seronegative children, and relative risk in excess of 1 was likely only at ages below nine years (Fig 6).

Implementing either vaccine scenario in our simulations required an estimate of RR per event, rather than RR over the course of a trial [16]. For a leaky vaccine, these two different interpretations of RR may vary depending on how many times study participants are exposed [56]. In S2 Text, we showed that these values of RR are unlikely to differ for a dengue vaccine by more than 5%. Given that relatively small difference and in the absence of more detailed information about the number of exposures that participants experienced during CTD-TDV trials, we operated under the assumption that these two interpretations of RR were equal.

Under our assumptions about how efficacy observed in trials derived from two different forms of protection, an assumption of equal parts protection against infection and protection against disease (i.e., *p* = 0.5) gave, on average, relative risks of 48% for either infection or disease in seropositive nine-year olds and 80% for either in seronegative nine-year olds (Fig 6). In the event that 90% of protection derived from protection against disease and only 10% from protection against infection (i.e., *p* = 0.1), the relative risk for seropositive nine-year-olds was 27% for disease and 87% for infection and 68% for disease and 96% for infection for seronegative nine-year-olds (Fig 6).

### Vaccination impact projections

#### Example vaccination impact projections

**Fig 7:**
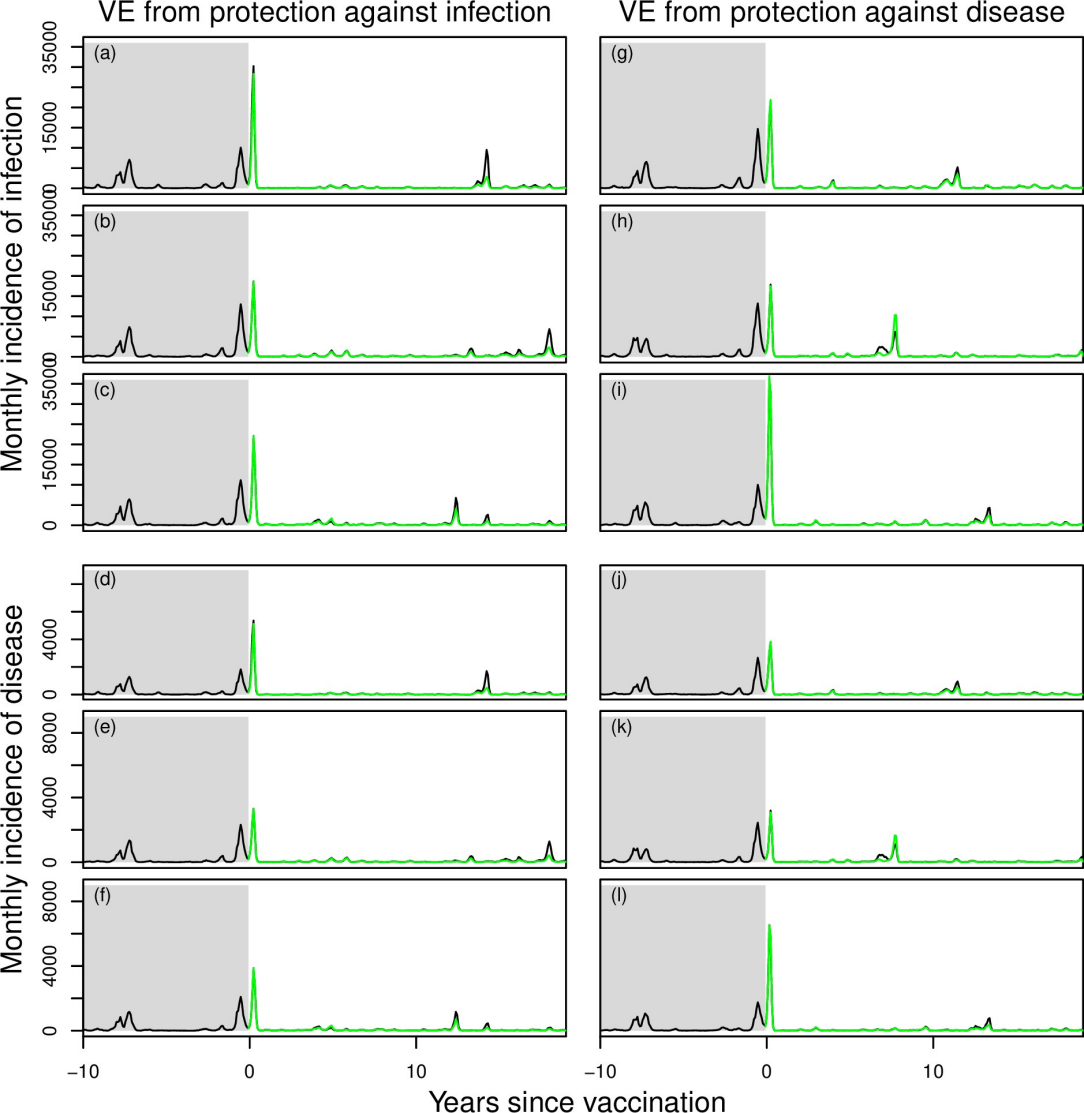
Examples of paired time series of annual incidence of human DENV infections simulated from the model with vaccination (green) and without (black). Prior to year 0, both simulations in each pair are identical and follow dynamics calibrated as shown in Fig 2. Beginning in year 0, routine vaccination commences in the simulation colored in green, but not in the one in black. Three different realizations are shown for each of two scenarios (left: p = 1; right: p = 0), with different outcomes from the same realization plotted in each of four sets of panels: a, d, g, and j; b, e, h, and k; c, f, i, and l.

In three pairs of simulations in which the CYD-TDV vaccine derived its efficacy from protection against infection (*p* = 1), epidemic size was noticeably smaller during outbreak years in simulations with vaccination (Fig 7, green lower than black in left column). In three pairs of simulations in which the vaccine derived its efficacy from protection against disease but not infection (*p* = 0), epidemic size was somewhat lower in terms of incidence of disease but essentially identical in terms of incidence of infection (Fig 7, green similar to black in right column). Across larger numbers of simulations, we sometimes observed that a negative proportion of infections or disease episodes was averted by vaccination (i.e., there were more cumulative infections or disease episodes in the simulation with vaccination). This was a result of occasional chance differences in paired simulations with and without vaccination, due to the fact that random number seeds could maintain identical behavior with respect to some processes but not all.

#### Ensemble projections of CYD-TDV impact

**Fig 8:**
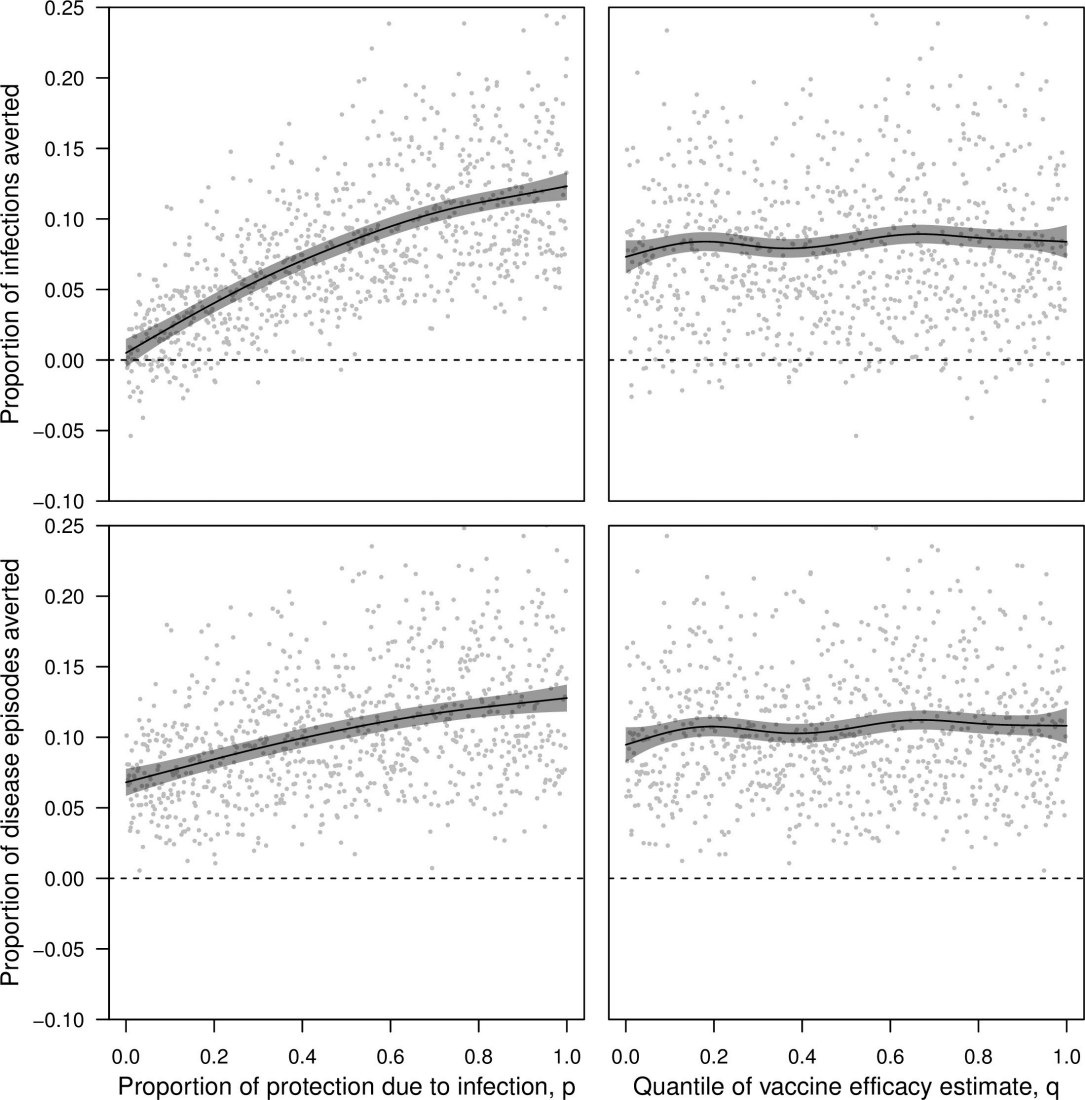
Impacts of vaccination assessed in 1,000 pairs of simulations with and without vaccination under the CYD-TDV vaccine profile. Simulation pairs varied with respect to the proportion of vaccine efficacy due to protection from infection, p, (left column) and the quantile of estimated vaccine efficacy, q (right column). The proportions of cumulative infections averted (top row) and cumulative disease episodes averted (bottom row) were based on the number of each in the simulation without vaccination minus the number of each in the simulation with vaccination, both following 20 years of routine vaccination of 9-year olds at 80% coverage. Lines show the proportion of cumulative infections and disease episodes averted as a function of p and q, as estimated by a generalized additive model with independent smooth terms for p and q. When one of p or q is varied, the other is held constant at 0.5. Gray bands indicate 95% confidence intervals.

Simulations conducted with the CYD-TDV vaccine across the full range of the parameter *p* showed that, on average, the proportion of infections averted (0.123, 95% CI: 0.113–0.133) and the proportion of disease episodes averted (0.128, 95% CI: 0.118–0.137) were both maximized when vaccine efficacy derived from protection against infection (*p* = 1) (Fig 8, left). Under the opposite extreme (*p* = 0), a lower proportion of disease episodes were averted (0.068, 95% CI: 0.059–0.078), reflecting direct protection of vaccinees. The proportion of infections averted was approximately zero under this scenario (0.005, 95% CI: -0.005–0.015), as infections experienced neither by vaccinees nor by others were prevented by a vaccine that had no impact on the ability of vaccinees to transmit DENV upon becoming infected. Differences in the proportion of infections averted and the proportion of disease episodes averted were not as large across the range of uncertainty in VE captured by *q* (Fig 8, right). Across the full range of *q* (holding *p* at 0.5), the proportion of infections averted varied from 0.073 (95% CI: 0.061–0.085) to 0.084 (95% CI: 0.072–0.096) (Fig 8, right). Results for disease episodes averted were similar (from 0.095, 95% CI: 0.082–0.107, to 0.108, 95% CI: 0.096–0.121).

#### Ensemble projections of generic dengue vaccine impact

**Fig 9:**
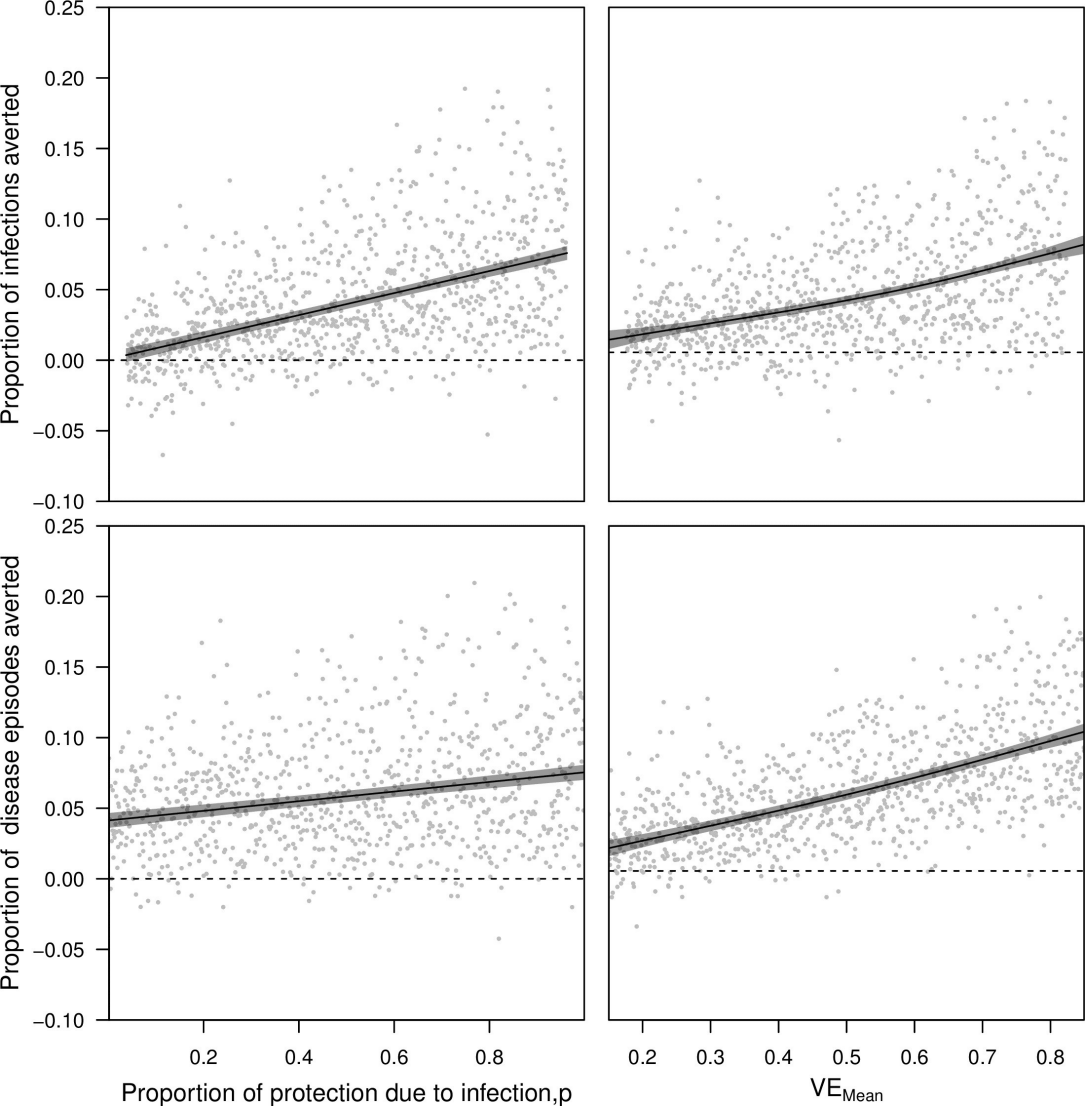
Impacts of vaccination assessed in 1,000 pairs of simulations with and without vaccination under the generic dengue vaccine profile. Simulation pairs varied with respect to the proportion of vaccine efficacy due to protection from infection, p, (left column) and mean vaccine efficacy, VE_mean_ (right column). The proportion of cumulative infections averted (top row) and cumulative disease episodes averted (bottom row) were based on the number of each in the simulation without vaccination minus the number of each in the simulation with vaccination, both following 20 years of routine vaccination of 9-year olds at 80% coverage. Lines show the proportion of infections or diseases episodes averted as a function of each parameter varied on the x-axis, as estimated by a generalized additive model with independent smooth terms for each parameter. When one parameter is varied, the other is held constant at the midpoint of its range, as are VE_serostatus_ and VE_serotype_. Gray bands indicate 95% confidence intervals.

Simulations conducted with the generic dengue vaccine across the range of *p* also showed that proportions of infections averted (0.076, 95% CI: 0.071–0.081) and disease episodes averted (0.075, 95% CI: 0.070–0.081) were maximized when vaccine efficacy derived from protection against infection (*p* = 1) (holding other parameters at the midpoints of their ranges: VE_mean_ = 0.5, VE_serostatus_ = 0.075, VE_serotype_ = 0.075) (Fig 9, left column). Likewise, the lowest proportions of infections averted (0.004, 95% CI: -0.001–0.008) and disease episodes averted (0.041, 95% CI: 0.036–0.047) were observed when *p* = 0. Because a much broader range of VE_mean_ was explored in these simulations than was represented by the range of *q* in simulations with the CYD-TDV vaccine, the range of proportions of infections and disease episodes averted was much larger across the range of VE_mean_ (Fig 9, right column) than across the range of *q* (Fig 8, right column). Differences in vaccine efficacy associated with serostatus and serotype had a negligible effect on proportions of infections and disease episodes averted (S23 Fig).

#### Summary across models of vaccine profile

**Fig 10:**
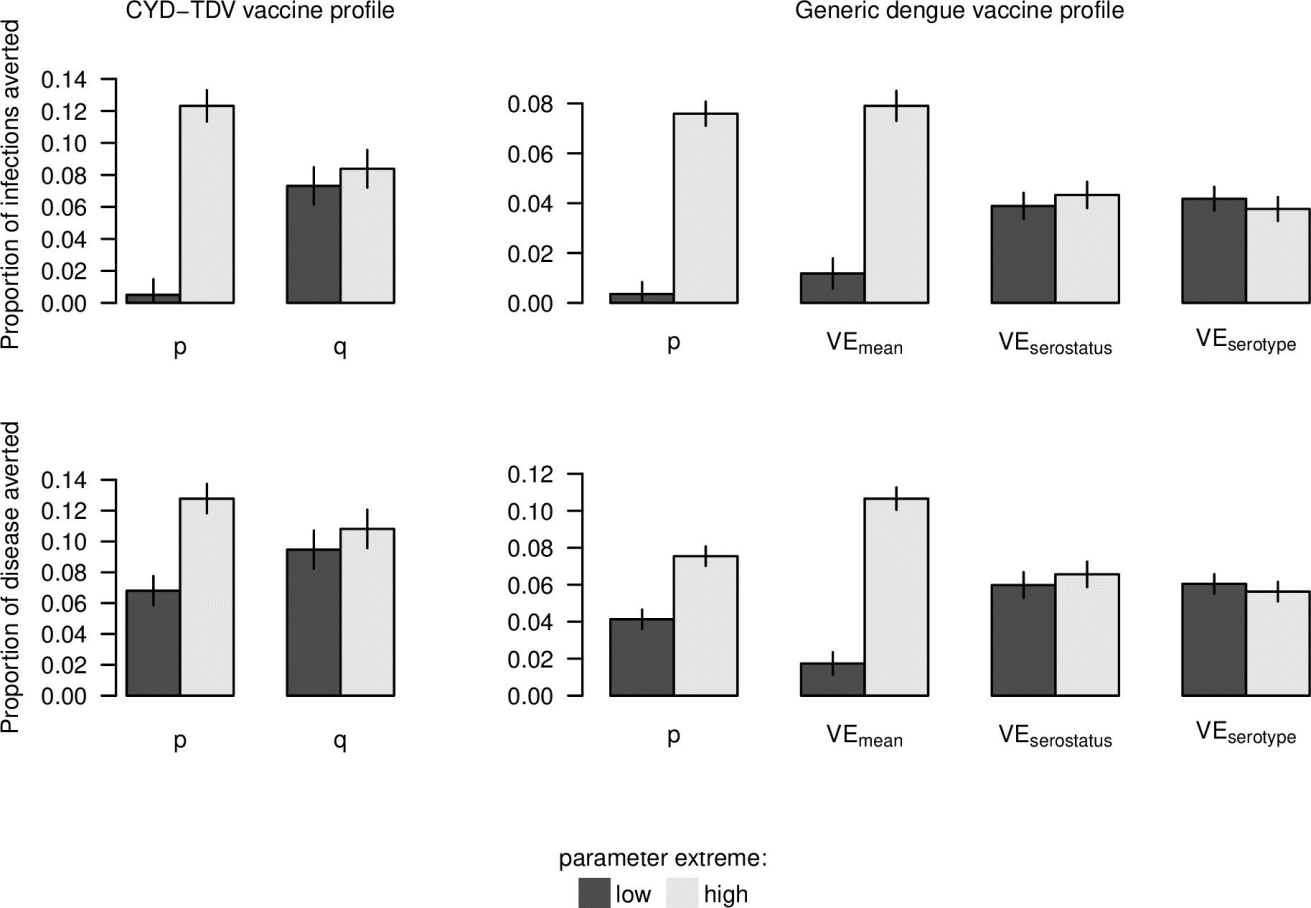
Summary of vaccination impact on infections and disease episodes (rows) under two sets of assumptions about vaccine profile (columns) at extreme values of parameters that varied across simulations (colors). Bars display point estimates and 95% confidence intervals obtained from fitting generalized additive models to simulation results across the range of each parameter while holding others at the midpoints of their ranges. These values correspond to the extremes displayed in Fig 8 (p, q), Fig 9 (p, VE_mean_), and S23 Fig (VE_serostatus_, VE_serotype_).

Under both models of vaccine profile that we considered, the assumption that efficacy derives from protection against disease (*p* = 0) resulted in essentially no infections averted and relatively modest disease episodes averted (Fig 10). In contrast, the assumption that efficacy derives from protection against infection (*p* = 1) resulted in disease episodes averted that were nearly twofold higher (median: 1.76; 95% CI: 1.54–2.06). In comparison, the extent of variation in infections and disease episodes averted that was explained by variation across VE quantiles (*q*: 0–1) was small for the CYD-TDV vaccine. For the generic dengue vaccine, the proportion of disease episodes averted across the range of uncertainty in *p* (0–1) was equivalent to a difference in VE_mean_ of 0.268 (95% CI: 0.210–0.329). Differences in vaccination impact due to differences in VE_serostatus_ and VE_serotype_ were more modest.

### Sensitivity analysis

Under both models of vaccine profile that we considered, alternative parameter values yielded results that were largely similar to those under default parameter values. Across all scenarios with the CYD-TDV vaccine, differences in the proportion of infections and disease episodes averted were relatively large across the range of *p* but small across the range of *q* (S24–S31 Figs). Across all scenarios with the generic dengue vaccine, differences in the proportion of infections and disease episodes averted were large across the ranges of *p* and VE_mean_ and small across the ranges of VE_serostatus_ and VE_serotype_ (S32–S39 Figs).

## Discussion

We developed an agent-based model for DENV transmission and used it to assess the extent of uncertainty in dengue vaccination impact projections attributable to uncertainty about breakthrough infections. Our analysis was not intended to represent a comprehensive assessment of the suitability of the CYD-TDV vaccine as a public health tool or to make a recommendation about its use. Instead, the value of this analysis is that it provides an assessment of the extent to which a potentially important source of uncertainty about vaccine profile might affect vaccination impact projections. Our results indicate that uncertainty about the extent to which a dengue vaccine prevents breakthrough infections makes a contribution to uncertainty about vaccination impact equivalent to not knowing whether VE is 0.70 or 0.43. In the event that information about breakthrough infections remains lacking, limiting a model to a single assumption about this aspect of a vaccine’s profile could result in the communication of recommendations to decision makers that convey a false sense of confidence.

Across two sets of assumptions about aspects of vaccine profile unrelated to breakthrough infections and thirteen different assumptions about model parameterization, our results were consistent in their suggestion that the projected impact of routine vaccination of nine-year olds is sensitive to the extent to which vaccination prevents breakthrough infections. These findings mirror conclusions from impact projections of vaccines for herpes simplex virus [10], malaria [21], and tuberculosis [57]; i.e., that vaccines that protect against infection should result in greater indirect protection of unvaccinated people than vaccines that primarily protect against disease. Compared to numerical uncertainty about VE for CYD-TDV, uncertainty about breakthrough infections contributed much more to uncertainty about vaccination impact in our analysis. This does not mean that precise numerical estimates of VE are unimportant, but instead that uncertainty about VE following trials is typically low relative to uncertainty about the degree to which efficacy derives from protection against infection or protection against disease. Echoing this, our results using a generic dengue vaccine across a wide range of VE show that the numerical value of vaccine efficacy is indeed important. Efficacy trials substantially narrow that range, because that is precisely what they are designed to do. At present, however, they are generally not designed to narrow uncertainty about breakthrough infections.

An important question following our analysis is whether, and how, information about protection against infection could be obtained empirically for a dengue vaccine. Disease is typically the preferred endpoint in efficacy trials, but arguments in favor of an infection endpoint can also be made in certain cases [58]. For example, the possibility of a secondary endpoint of infection has been proposed for efficacy trials for Zika vaccines [59]. Motivations for this include the infrequent occurrence of the disease outcome of greatest interest (congenital Zika syndrome, CZS) [60] and a clearer relationship between Zika virus infection and CZS than between more common disease outcomes and CZS [61]. At the same time, the short duration of Zika virus infection [62] makes reliable detection of active infection impractical, and issues such as cross-reactivity with other flaviviruses [63] make serological surrogates of infection problematic. Similar challenges would likely apply to estimation of efficacy against an infection endpoint for dengue vaccines. In the event that these challenges for endpoint measurement could be overcome, RR_inf|exp_ could then be estimated directly (e.g., [64,65]). Until that happens, our work demonstrates that uncertainty in RR_inf|exp_ can (and should) be accounted for in vaccination impact projections.

The importance of understanding the extent of breakthrough infections owes to their critical role in determining the extent of indirect protection from vaccination. Still other factors are expected to further modulate indirect protection in a given context [22]. First, vaccination coverage—and, for that matter, naturally acquired immunity—clearly has an influence, with high coverage potentially compensating somewhat for imperfections in a vaccine’s profile [66]. Second, contact structure is thought to play an important role in determining the extent of indirect protection [24,25]. Recent modeling analyses of influenza [67] and onchocerciasis [68] showed that realistic assumptions about contact structure can lead to substantial differences in intervention impact projections. Although we view our own realistic portrayal of mosquito-human contact patterns as a strength of our model, the realism of a model’s assumptions does not necessarily translate into accuracy of a model’s predictions. To address that issue, uncertainty about model structure can be propagated into uncertainty about vaccination impact by combining projections from models with diverse structures and assumptions, as has been done recently for malaria [69], dengue [14], and pneumococcal disease [7].

Although our model of vaccine efficacy is consistent with several key findings from clinical trials of CYD-TDV [16], such as serostatus- and age-dependent efficacy against disease, there are other findings that we did not account for. One notable feature of CYD-TDV that we have not considered pertains to protection against severe disease [70]. In particular, to the extent that vaccination serves as a “primary-like” infection in seronegative vaccine recipients [52], the incidence of severe disease could increase as transmission is lowered by indirect effects of vaccination and the proportion of seronegative vaccine recipients increases [71]. Clinical trial data indicate, however, that whatever protection against severe disease the vaccine does afford may wane within a few years of vaccination [16]. In addition, direct protection against severe disease could affect indirect protection of unvaccinated individuals, given that severe disease could be associated with heightened [20] or attenuated [72] infectiousness. At the same time, associations between disease severity and infectiousness may trade off with other factors [73], such as limited mobility [74]. In the end though, the relatively infrequent occurrence of severe disease may limit the overall impact of such effects on population-level transmission [20].

Although we expect that our overall conclusions have implications beyond the context of dengue in Iquitos, we note that our quantitative projections of cumulative proportions of infections and disease episodes averted are not directly applicable outside this setting. These numerical results could vary as a function of vaccination coverage, overall transmission intensity, and many other factors, similar to how estimates of vaccine efficacy can be context-dependent [75,76]. That said, our projections of CYD-TDV vaccination impact are in rough agreement with a study based on an earlier version of our model and seven others [14]. We hope that our model’s detailed representation of a well-studied, dengue-endemic population provides a tool for future studies to explore additional questions about vaccination impact in ways that acknowledge realistic variability in transmission patterns of the four DENV serotypes.

## Supporting information

S1 Text Detailed model description. (PDF)

S2 Text Impact of number of exposures on the interpretation of vaccine efficacy. (PDF)

S3 Text Supplemental references. (PDF)

S1 Fig Marginal distributions of parameters describing the scaling relationship between mosquito emergence derived from Reiner et al. [49] and mosquito emergence used in the model. Calibration iteration refers to the iteration in the calibration process to which these distributions apply, with labels corresponding to the years up to which the calibration applies (e.g., 00 corresponds to 2000) or to calibration iterations involving all data (i.e., All^1^ and All^2^). Two parameters were used due to switch in data collection methods used in empirical work underlying our description of mosquito population dynamics.
(TIF)

S2 Fig Marginal distributions of parameters describing the modal timing of DENV-1 introductions into the simulated population in each year. Calibration iteration refers to the iteration in the calibration process to which these distributions apply, with labels corresponding to the years up to which the calibration applies (e.g., 00 corresponds to 2000) or to calibration iterations involving all data (i.e., All^1^ and All^2^).
(TIF)

S3 Fig Marginal distributions of parameters describing the modal timing of DENV-2 introductions into the simulated population in each year. Calibration iteration refers to the iteration in the calibration process to which these distributions apply, with labels corresponding to the years up to which the calibration applies (e.g., 00 corresponds to 2000) or to calibration iterations involving all data (i.e., All^1^ and All^2^).
(TIF)

S4 Fig Marginal distributions of parameters describing the modal timing of DENV-3 introductions into the simulated population in each year. Calibration iteration refers to the iteration in the calibration process to which these distributions apply, with labels corresponding to the years up to which the calibration applies (e.g., 00 corresponds to 2000) or to calibration iterations involving all data (i.e., All^1^ and All^2^).
(TIF)

S5 Fig Marginal distributions of parameters describing the modal timing of DENV-4 introductions into the simulated population in each year. Calibration iteration refers to the iteration in the calibration process to which these distributions apply, with labels corresponding to the years up to which the calibration applies (e.g., 00 corresponds to 2000) or to calibration iterations involving all data (i.e., All^1^ and All^2^). No DENV-3 importation was simulated in years in which DENV-3 was not observed empirically in Iquitos.
(TIF)

S6 Fig Marginal distributions of parameters for scaling the magnitude of DENV-1 introductions into the simulated population in each year. Calibration iteration refers to the iteration in the calibration process to which these distributions apply, with labels corresponding to the years up to which the calibration applies (e.g., 00 corresponds to 2000) or to calibration iterations involving all data (i.e., All^1^ and All^2^). No DENV-4 importation was simulated in years in which DENV-4 was not observed empirically in Iquitos.
(TIF)

S7 Fig Marginal distributions of parameters for scaling the magnitude of DENV-2 introductions into the simulated population in each year. Calibration iteration refers to the iteration in the calibration process to which these distributions apply, with labels corresponding to the years up to which the calibration applies (e.g., 00 corresponds to 2000) or to calibration iterations involving all data (i.e., All^1^ and All^2^).
(TIF)

S8 Fig Marginal distributions of parameters for scaling the magnitude of DENV-3 introductions into the simulated population in each year. Calibration iteration refers to the iteration in the calibration process to which these distributions apply, with labels corresponding to the years up to which the calibration applies (e.g., 00 corresponds to 2000) or to calibration iterations involving all data (i.e., All^1^ and All^2^). No DENV-3 importation was simulated in years in which DENV-3 was not observed empirically in Iquitos.
(TIF)

S9 Fig Marginal distributions of parameters for scaling the magnitude of DENV-4 introductions into the simulated population in each year. Calibration iteration refers to the iteration in the calibration process to which these distributions apply, with labels corresponding to the years up to which the calibration applies (e.g., 00 corresponds to 2000) or to calibration iterations involving all data (i.e., All^1^ and All^2^). No DENV-4 importation was simulated in years in which DENV-4 was not observed empirically in Iquitos.
(TIF)

S10 Fig Correlations between 64 parameters in the joint distribution of model parameters at each iteration of the calibration. Calibration iteration refers to the iteration in the calibration process to which these distributions apply, with labels corresponding to the years up to which the calibration applies (e.g., 00 corresponds to 2000) or to calibration iterations involving all data (i.e., All^1^ and All^2^). Each line indicates the trajectory of the correlation between a given pair of parameters over the course of the calibration process.
(TIF)

S11 Fig Monthly, serotype-specific incidence of infection per capita, as estimated by Reiner et al. [49] (gray bands) and as reproduced by our calibrated model (colored bands). Bands show the range of values in which 95% of simulated values lie for a given serotype in a given month. These values were obtained under the assumption that the net infectiousness of asymptomatic infections is half that of symptomatic infections. Other assumptions followed the default set of assumptions.
(TIF)

S12 Fig Monthly, serotype-specific incidence of infection per capita, as estimated by Reiner et al. [49] (gray bands) and as reproduced by our calibrated model (colored bands). Bands show the range of values in which 95% of simulated values lie for a given serotype in a given month. These values were obtained under the assumption that the average duration of temporary cross-immunity is 180 days. Other assumptions followed the default set of assumptions.
(TIF)

S13 Fig Monthly, serotype-specific incidence of infection per capita, as estimated by Reiner et al. [49] (gray bands) and as reproduced by our calibrated model (colored bands). Bands show the range of values in which 95% of simulated values lie for a given serotype in a given month. These values were obtained under the assumption that the duration of temporary cross-immunity was identical for all individuals. Other assumptions followed the default set of assumptions.
(TIF)

S14 Fig Monthly, serotype-specific incidence of infection per capita, as estimated by Reiner et al. [49] (gray bands) and as reproduced by our calibrated model (colored bands). Bands show the range of values in which 95% of simulated values lie for a given serotype in a given month. These values were obtained under the assumption that mosquito infectiousness was 0.5. Other assumptions followed the default set of assumptions.
(TIF)

S15 Fig Monthly, serotype-specific incidence of infection per capita, as estimated by Reiner et al. [49] (gray bands) and as reproduced by our calibrated model (colored bands). Bands show the range of values in which 95% of simulated values lie for a given serotype in a given month. These values were obtained under the assumption that mosquito biting rate was half that under default assumptions. Other assumptions followed the default set of assumptions.
(TIF)

S16 Fig Monthly, serotype-specific incidence of infection per capita, as estimated by Reiner et al. [49] (gray bands) and as reproduced by our calibrated model (colored bands). Bands show the range of values in which 95% of simulated values lie for a given serotype in a given month. These values were obtained under the assumption that mosquito biting rate was double that under default assumptions. Other assumptions followed the default set of assumptions.
(TIF)

S17 Fig Monthly, serotype-specific incidence of infection per capita, as estimated by Reiner et al. [49] (gray bands) and as reproduced by our calibrated model (colored bands). Bands show the range of values in which 95% of simulated values lie for a given serotype in a given month. These values were obtained under the assumption that mosquito death rate was half that under default assumptions. Other assumptions followed the default set of assumptions.
(TIF)

S18 Fig Monthly, serotype-specific incidence of infection per capita, as estimated by Reiner et al. [49] (gray bands) and as reproduced by our calibrated model (colored bands). Bands show the range of values in which 95% of simulated values lie for a given serotype in a given month. These values were obtained under the assumption that mosquito death rate was double that under default assumptions. Other assumptions followed the default set of assumptions.
(TIF)

S19 Fig Monthly, serotype-specific incidence of infection per capita, as estimated by Reiner et al. [49] (gray bands) and as reproduced by our calibrated model (colored bands). Bands show the range of values in which 95% of simulated values lie for a given serotype in a given month. These values were obtained under the assumption that the extrinsic incubation period was half that under default assumptions. Other assumptions followed the default set of assumptions.
(TIF)

S20 Fig Monthly, serotype-specific incidence of infection per capita, as estimated by Reiner et al. [49] (gray bands) and as reproduced by our calibrated model (colored bands). Bands show the range of values in which 95% of simulated values lie for a given serotype in a given month. These values were obtained under the assumption that the extrinsic incubation period was double that under default assumptions. Other assumptions followed the default set of assumptions.
(TIF)

S21 Fig Monthly, serotype-specific incidence of infection per capita, as estimated by Reiner et al. [49] (gray bands) and as reproduced by our calibrated model (colored bands). Bands show the range of values in which 95% of simulated values lie for a given serotype in a given month. These values were obtained under the assumption that mosquito movement probability was 0.1. Other assumptions followed the default set of assumptions.
(TIF)

S22 Fig Monthly, serotype-specific incidence of infection per capita, as estimated by Reiner et al. [49] (gray bands) and as reproduced by our calibrated model (colored bands). Bands show the range of values in which 95% of simulated values lie for a given serotype in a given month. These values were obtained under the assumption that mosquito movement probability was 0.5. Other assumptions followed the default set of assumptions.
(TIF)

S23 Fig Impacts of vaccination assessed in 1,000 pairs of simulations with and without vaccination with the generic vaccine. Simulation pairs varied with respect to variation in vaccine efficacy associated with serostatus, VE_serostatus_, (second column) and variation in vaccine efficacy associated with serotype, VE_serotype_ (right column). The proportion of cumulative infections averted (top row) and cumulative disease episodes averted (bottom row) were based on the number of each in the simulation without vaccination minus the number of each in the simulation with vaccination, both following 20 years of routine vaccination of 9-year olds at 80% coverage. Lines show the proportion of infections or disease episodes averted as a function of each parameter varied on the x-axis, as estimated by a generalized additive model with independent smooth terms for each parameter. When one parameter is varied, the other is held constant at the midpoint of its range, as are *p* and VE_mean_. Gray bands indicate 95% confidence intervals.
(TIF)

S24 Fig Summary of vaccination impact on infections and disease episodes (columns) under the CYD-TDV vaccine profile at extreme values of parameters that varied across simulations (rows) under different assumptions about human infectiousness (colors). Bars display point estimates and 95% confidence intervals obtained from fitting generalized additive models to simulation results across the range of each parameter while holding others at the midpoints of their ranges. These values are comparable to the extremes displayed in Fig 8 (p, q) but under different assumptions about human infectiousness.
(TIF)

S25 Fig Summary of vaccination impact on infections and disease episodes (columns) under the CYD-TDV vaccine profile at extreme values of parameters that varied across simulations (rows) under different assumptions about the duration of cross-immunity (colors). Bars display point estimates and 95% confidence intervals obtained from fitting generalized additive models to simulation results across the range of each parameter while holding others at the midpoints of their ranges. These values are comparable to the extremes displayed in Fig 8 (p, q) but under different assumptions about the duration of cross-immunity.
(TIF)

S26 Fig Summary of vaccination impact on infections and disease episodes (columns) under the CYD-TDV vaccine profile at extreme values of parameters that varied across simulations (rows) under different assumptions about inter-individual variability in the duration of cross-immunity (colors). Bars display point estimates and 95% confidence intervals obtained from fitting generalized additive models to simulation results across the range of each parameter while holding others at the midpoints of their ranges. These values are comparable to the extremes displayed in Fig 8 (p, q) but under different assumptions about inter-individual variability in the duration of cross-immunity.
(TIF)

S27 Fig Summary of vaccination impact on infections and disease episodes (columns) under the CYD-TDV vaccine profile at extreme values of parameters that varied across simulations (rows) under different assumptions about mosquito infectiousness (colors). Bars display point estimates and 95% confidence intervals obtained from fitting generalized additive models to simulation results across the range of each parameter while holding others at the midpoints of their ranges. These values are comparable to the extremes displayed in Fig 8 (p, q) but under different assumptions about mosquito infectiousness.
(TIF)

S28 Fig Summary of vaccination impact on infections and disease episodes (columns) under the CYD-TDV vaccine profile at extreme values of parameters that varied across simulations (rows) under different assumptions about mosquito biting rate (colors). Bars display point estimates and 95% confidence intervals obtained from fitting generalized additive models to simulation results across the range of each parameter while holding others at the midpoints of their ranges. These values are comparable to the extremes displayed in Fig 8 (p, q) but under different assumptions about mosquito biting rate.
(TIF)

S29 Fig Summary of vaccination impact on infections and disease episodes (columns) under the CYD-TDV vaccine profile at extreme values of parameters that varied across simulations (rows) under different assumptions about mosquito death rate (colors). Bars display point estimates and 95% confidence intervals obtained from fitting generalized additive models to simulation results across the range of each parameter while holding others at the midpoints of their ranges. These values are comparable to the extremes displayed in Fig 8 (p, q) but under different assumptions about mosquito death rate.
(TIF)

S30 Fig Summary of vaccination impact on infections and disease episodes (columns) under the CYD-TDV vaccine profile at extreme values of parameters that varied across simulations (rows) under different assumptions about the extrinsic incubation period (colors). Bars display point estimates and 95% confidence intervals obtained from fitting generalized additive models to simulation results across the range of each parameter while holding others at the midpoints of their ranges. These values are comparable to the extremes displayed in Fig 8 (p, q) but under different assumptions about the extrinsic incubation period.
(TIF)

S31 Fig Summary of vaccination impact on infections and disease episodes (columns) under the CYD-TDV vaccine profile at extreme values of parameters that varied across simulations (rows) under different assumptions about mosquito movement probability (colors). Bars display point estimates and 95% confidence intervals obtained from fitting generalized additive models to simulation results across the range of each parameter while holding others at the midpoints of their ranges. These values are comparable to the extremes displayed in Fig 8 (p, q) but under different assumptions about mosquito movement probability.
(TIF)

S32 Fig Summary of vaccination impact on infections and disease episodes (columns) under the generic dengue vaccine profile at extreme values of parameters that varied across simulations (rows) under different assumptions about human infectiousness (colors). Bars display point estimates and 95% confidence intervals obtained from fitting generalized additive models to simulation results across the range of each parameter while holding others at the midpoints of their ranges. These values are comparable to the extremes displayed in Fig 9 (p, VE_mean_) and S23 Fig (VE_serostatus_, VE_serotype_) but under different assumptions about human infectiousness.
(TIF)

S33 Fig Summary of vaccination impact on infections and disease episodes (columns) under the generic dengue vaccine profile at extreme values of parameters that varied across simulations (rows) under different assumptions about the duration of cross-immunity (colors). Bars display point estimates and 95% confidence intervals obtained from fitting generalized additive models to simulation results across the range of each parameter while holding others at the midpoints of their ranges. These values are comparable to the extremes displayed in Fig 9 (p, VE_mean_) and S23 Fig (VE_serostatus_, VE_serotype_) but under different assumptions about the duration of cross-immunity.
(TIF)

S34 Fig Summary of vaccination impact on infections and disease episodes (columns) under the generic dengue vaccine profile at extreme values of parameters that varied across simulations (rows) under different assumptions about inter-individual variability in the duration of cross-immunity (colors). Bars display point estimates and 95% confidence intervals obtained from fitting generalized additive models to simulation results across the range of each parameter while holding others at the midpoints of their ranges. These values are comparable to the extremes displayed in Fig 9 (p, VE_mean_) and S23 Fig (VE_serostatus_, VE_serotype_) but under different assumptions about inter-individual variability in the duration of cross-immunity.
(TIF)

S35 Fig Summary of vaccination impact on infections and disease episodes (columns) under the generic dengue vaccine profile at extreme values of parameters that varied across simulations (rows) under different assumptions about mosquito infectiousness (colors). Bars display point estimates and 95% confidence intervals obtained from fitting generalized additive models to simulation results across the range of each parameter while holding others at the midpoints of their ranges. These values are comparable to the extremes displayed in Fig 9 (p, VE_mean_) and S23 Fig (VE_serostatus_, VE_serotype_) but under different assumptions about mosquito infectiousness.
(TIF)

S36 Fig Summary of vaccination impact on infections and disease episodes (columns) under the generic dengue vaccine profile at extreme values of parameters that varied across simulations (rows) under different assumptions about mosquito biting rate (colors). Bars display point estimates and 95% confidence intervals obtained from fitting generalized additive models to simulation results across the range of each parameter while holding others at the midpoints of their ranges. These values are comparable to the extremes displayed in Fig 9 (p, VE_mean_) and S23 Fig (VE_serostatus_, VE_serotype_) but under different assumptions about mosquito biting rate.
(TIF)

S37 Fig Summary of vaccination impact on infections and disease episodes (columns) under the generic dengue vaccine profile at extreme values of parameters that varied across simulations (rows) under different assumptions about mosquito death rate (colors). Bars display point estimates and 95% confidence intervals obtained from fitting generalized additive models to simulation results across the range of each parameter while holding others at the midpoints of their ranges. These values are comparable to the extremes displayed in Fig 9 (p, VE_mean_) and S23 Fig (VE_serostatus_, VE_serotype_) but under different assumptions about mosquito death rate.
(TIF)

S38 Fig Summary of vaccination impact on infections and disease episodes (columns) under the generic dengue vaccine profile at extreme values of parameters that varied across simulations (rows) under different assumptions about the extrinsic incubation period (colors). Bars display point estimates and 95% confidence intervals obtained from fitting generalized additive models to simulation results across the range of each parameter while holding others at the midpoints of their ranges. These values are comparable to the extremes displayed in Fig 9 (p, VE_mean_) and S23 Fig (VE_serostatus_, VE_serotype_) but under different assumptions about the extrinsic incubation period.
(TIF)

S39 Fig Summary of vaccination impact on infections and disease episodes (columns) under the generic dengue vaccine profile at extreme values of parameters that varied across simulations (rows) under different assumptions about mosquito movement probability (colors). Bars display point estimates and 95% confidence intervals obtained from fitting generalized additive models to simulation results across the range of each parameter while holding others at the midpoints of their ranges. These values are comparable to the extremes displayed in Fig 9 (p, VE_mean_) and S23 Fig (VE_serostatus_, VE_serotype_) but under different assumptions about mosquito movement probability.
(TIF)

S40 FigRelationship between vaccine efficacy against disease (VE) and per-exposure protection, *θ*, for different values of the infection attack rate, FOIΔt, over the period of a vaccine trial.(TIF)
