# Supplementary material for: An agent-based model of dengue virus transmission shows how uncertainty about breakthrough infections influences vaccination impact projections
Source: PLoS Comput Biol. 2019 Mar 20;15(3):e1006710. doi: 10.1371/journal.pcbi.1006710 (PMC6443188; doi:10.1371/journal.pcbi.1006710)
Supplement: S1 Text — (PDF) [file pcbi.1006710.s001.pdf]

S1 Text. Detailed model description.

for

An agent-based model of dengue virus transmission shows how uncertainty about breakthrough infections influences vaccination impact projections

T. Alex Perkins[1,2,$,*], Robert C. Reiner[2,3,$], Guido España[1,$], Quirine A. ten Bosch[1], Amit Verma[4], Kelly A. Liebman[5], Valerie A. Paz-Soldan[6], John P. Elder[7], Amy C. Morrison[5], Steven T. Stoddard[7], Uriel Kitron[2,8], Gonzalo M. Vazquez-Prokopec[2,8], Thomas W. Scott[2,5], David L. Smith[2,9]

[1] Department of Biological Sciences and Eck Institute for Global Health, University of Notre Dame, Notre Dame, IN, USA
[2] Fogarty International Center, National Institutes of Health, Bethesda, MD, USA
[3] Department of Epidemiology and Biostatistics, Indiana University, Bloomington, IN, USA
[4] Center for Disease Dynamics, Economics, and Policy, Washington, DC, USA
[5] Department of Entomology and Nematology, University of California, Davis, CA, USA
[6] Department of Global Community Health and Behavioral Sciences, Tulane University School of Public Health and Tropical Medicine, New Orleans, LA, USA
[7] Institute for Behavioral and Community Health, Graduate School of Public Health, San Diego State University, San Diego, CA, USA
[8] Department of Environmental Sciences, Emory University, Atlanta, GA, USA
[9] Institute for Health Metrics and Evaluation, University of Washington, Seattle, WA, USA

$ Denotes equal contributions
* Author for correspondence: taperkins@nd.edu

Detailed Model Description

This model description follows the ODD (Overview, Design concepts, Details) protocol for agent-based models [29,30]. Given how complex agent-based models can be, the ODD protocol was developed as a way to standardize the description of these models throughout ecology and other fields. This model has been implemented as a software program using object-oriented code in C++.

Purpose

The model simulates transmission of dengue virus (DENV) between individual people and mosquitoes that occupy a landscape of discrete locations where they encounter each other. The model also simulates the impact of a hypothetical dengue vaccine on the occurrence of DENV infection and on the occurrence of symptomatic disease associated with DENV infection within the simulated human population.

Entities, state variables, and scales

The model focuses on five primary entities: individual people, individual mosquitoes, infections, locations, and vaccines. Individual people have the following state variables: a location that is designated as the individual's home, an activity space, gender, body size, infection status, infection history, and immune status. Individual mosquitoes have the following state variables: location and infection status. All individual mosquitoes are adult female *Aedes aegypti*. Infections have the following state variables: DENV serotype 1-4, day of infection, and, for infections in humans, day of recovery and day of immune acquisition. Locations have the following state variables: longitudinal and latitudinal coordinates of the location's centroid, location type (residential, commercial, recreation, education, health care, religion, institutions, or others), and daily emergence rates of new adult mosquitoes. Vaccines have the following two state variables: efficacy against disease as a function of the age and pre-exposure history of the vaccinee, and the proportion of that efficacy attributable to protection against disease conditional on infection versus protection against infection conditional on exposure. Each of these five entities is defined by its own object class in our code.

For purposes of software implementation, our model is, on one level, iterated on a daily basis. On another level and for most processes, however, our model treats time in a continuous fashion because it is fundamentally an event-based model, analogous to the Gillespie algorithm [77]. We made this decision to minimize inaccuracies associated with lumping and discretizing events that occur continuously [78], to avoid being forced to make a fixed decision about the order of different types of events, and to allow for maximal precision in describing probabilistic distributions of waiting times for various stochastic processes. This precision is important not only for realistic modeling of the temporal dynamics of transmission but will be increasingly important in the future to realistically account for the sometimes subtle effects that mosquito-based interventions can have; e.g., reducing mosquito biting rates. The fundamental spatial unit in the model is the location, which we model after a city lot that each house or other building sits on. Altogether, our model applies to 40,839 lots, comprised primarily of homes but also including shops, markets, schools, churches, parks, and other locations. This constitutes the entirety of the core of the city of Iquitos, where the majority of relevant data collection has occurred over the last 15+ years and where roughly half of the entire metropolitan population lives.

Process overview and scheduling
The key processes in the model are 1) movement by individual people, 2) movement by mosquitoes, 3) mosquito emergence, 4) mosquito death, 5) mosquito blood-feeding, 6) infection of susceptible people by infectious mosquitoes or 7) vice versa, 8) changes in the infectiousness of individual people over time, 9) changes in the immune status of individual people, 10) demographic changes in the human population, and 11) vaccination. The first of these processes is pre-calculated and incorporated into the model as an input. The timing until an event of each of the other types occurs is represented as a continuous random variable, such that events occur at specific times of day. These random variables are drawn from probability distributions described separately for each process in the Submodels section. An illustration of the continuous timing of events related to mosquito-host encounters is shown in Fig. 1.

On a daily basis, the model iterates through each mosquito in the city and executes the events scheduled for that mosquito for that day in the order in which the events are scheduled to occur. One example of why this scheduling is important is if a mosquito is scheduled to both die and infect someone in the same day, the infection will never occur if death takes place first. Because events that apply to one mosquito have no effect on any other mosquitoes on that day, the order in which individual mosquitoes are processed is inconsequential. Mosquitoes are also assumed to have no effect on a human's status within a day, and instead rely only on each person's pre-scheduled whereabouts when selecting a person upon whom to blood-feed. This decoupling of event scheduling within versus across days is possible because the onset of infectiousness in both people and mosquitoes always takes longer than a single day [41,79].

Design concepts

Basic principles
The model seeks to leverage years of studies in Iquitos, Peru, quantifying heterogeneities in DENV transmission that manifest at individual, household, and neighborhood scales. Priorities for the model include realistically modeling individual human movement patterns and biting heterogeneity among individuals simultaneously co-located at a single location. We also seek to model details of infection dynamics and vaccine effects at an individual level in accordance with the best available data from clinical trials.

Emergence
Patterns of DENV infection in space and time are emergent properties of the model and are not predefined. Stochasticity is the major driver of a priori uncertainty about these patterns.

Adaptation
The mosquito's decision about whom to bite is influenced by the number of people present at a given time and each person's attractiveness to mosquitoes [32].

Objectives
The only entities in the model with any explicit objectives are mosquitoes, which seek to blood-feed. That said, they suffer no penalty nor reap any reward as a consequence of failing or succeeding in their quest to blood-feed.

Learning
None of the entities in the model have a learning capacity.

Prediction
None of the entities in the model have a predictive capacity.

Sensing
Each mosquito has the ability to sense the number of people present at its current location at a given time of day, as well as the attractiveness for blood-feeding on each person. The latter is a human state variable that depends on body surface area [32]. No mosquito is able to sense the presence or attractiveness of people at locations at which the mosquito is not currently present. As a consequence, their movement decisions are not affected by the presence or attractiveness of people at a location to which they might move.

Interaction
Mosquitoes interact with humans through blood-feeding and through the associated transmission of viruses in some cases. The movement trajectories of people are not affected by having been blood-fed upon.

Stochasticity
Prior to being incorporated into the model as an input, human movement trajectories are generated from a continuous-time Markov chain according to the algorithm by Perkins et al. [34]. Practically, this means that the duration of a visit to a given location is drawn from an exponential distribution and that the next location an individual visits is drawn from a categorical distribution over all other locations in an individual's activity space. The rates associated with these exponential distributions are related to the duration of visits to each location, and the probabilities of the categorical distributions are related to the frequency of visits to each location. Proceeding each day after initialization, one of the individual's daily trajectories is selected with equal probability.

Mosquito lifespan is drawn from an exponential distribution at the time of a mosquito's emergence. Mosquito movement is also highly stochastic, with the decision to stay or leave a location on a given day determined by a Bernoulli trial and the mosquito's destination location drawn randomly from the set of all locations within 50 meters with even probability. The elapsed time before a mosquito blood-feeds again is drawn from an exponential distribution.

A mosquito blood-feeds on a person at its current location, with the identity of that person determined by a random draw from a categorical distribution with probabilities proportional to each person's biting attractiveness [32]. If either a mosquito is infectious and a person susceptible or vice versa, an infection results depending on the outcome of a Bernoulli trial. Thenceforth, the duration of the incubation period in a given mosquito is drawn randomly from a predefined lognormal distribution specified based on empirical studies [41]. Lastly, the duration of temporary cross-immunity in a given person is drawn randomly from an exponential distribution. Given uncertainty in the literature about the duration of temporary cross-immunity, we considered three different values that spanned the majority of variation in values assumed by other models: 180, 365, and 686 days [45]. We also examined alternative scenarios about whether the duration of temporary cross-immunity among individuals was fixed or exponentially distributed.

For the sake of comparability across analyses, we use two distinct random number seeds in each simulation. One applies to events that directly involve vaccines or viruses, whereas the other applies to other events, most of which have to do with demographic events, movement, and human-mosquito contact. This allows for the landscape of human-mosquito encounters to

unfold identically across multiple simulations in which aspects of vaccination can be varied separately.

Collectives
Each person is assigned to a home location and as such is part of a household. Membership in a household comes with no special properties in the model other than the general tendency to spend more time at that common location than they would otherwise. Altogether, we considered a population of 200,000 people at the beginning of the simulation living in 38,835 houses within a total landscape of 40,839 locations.

Observation
The model is capable of producing a variety of different output files that report infections and other events, either individually or aggregated temporally.

<u>Initialization</u>
The composition of each house (i.e., how many residents plus each person's age, sex, and body size) was obtained by simulating a population with house-level demographic profiles consistent with available survey data but conforming to a desired total population size and age and gender distribution [80].

The first step in the population simulation algorithm was to simulate household sizes that yielded the correct overall population size. To do so, we weighted the distribution of household sizes in the survey data by a geometric probability mass function with a fitted parameter p=0.34. Sampling 38,835 houses weighted in this way yielded an appropriately sized overall population of 200,000 individuals on average. The second step in the population simulation algorithm was to randomly draw demographic profiles for houses of each size from the survey data. We populated houses serially, keeping track of the number of simulated individuals of each age and sex as the simulation proceeded. Once a given age-sex combination was exhausted in the target age and sex distribution, we attempted to replace the individual in question with one of the same sex and age class (i.e., children under 18, adults 18+). Near the end of the population simulation routine, however, we deviated from the target age class and/or sex of the simulated individual in question. For example, this resulted in some of the very last simulated houses being inhabited by several adult men, which was a household profile not observed in the survey data but one that was necessary to obtain a realistic age and sex distribution for the population as a whole.

Once the initial population was simulated, we simulated each person's serostatus for each of the four DENV serotypes as a function of that person's age and a parameter describing a baseline historical force of infection for each serotype prior to the time period of the simulation. We informed those parameters based on estimates of serotype-specific population seroprevalence at the beginning of the time period of the simulation as estimated by Reiner et al. [49]. No active infections were present at the time of initialization but instead accumulated over time in response to infections in temporary individuals that seeded transmission.

The number of mosquitoes that emerge in each location on each day of the simulation is simulated as a Poisson random variable according to a rate that is specific to each location on each day. The model was initialized with the rate for each location on the first day of the calibration period, which was January 1, 2000. The derivation of this model is described in the "Mosquito emergence" portion of the Submodels section.

Input
Each realization of the model depends on inputs from one master input file and nine additional input files. The master input file specifies the other input files and values of parameters that vary across simulations, with each row of the file corresponding to a distinct simulation. The first input file referred to by the master input file describes attributes of the vaccine and of vaccination strategy, such as relative risk of infection or disease among vaccinees, vaccination coverage, and the age of routine vaccination. The second file specifies which variables will be outputted from the model and how they are to be aggregated with respect to age groups or in time. The third input file specifies assumptions about the probability of developing symptomatic disease conditional on whether an individual is experiencing a primary, secondary, or post-secondary DENV infection. The fourth input file contains rows that each describe a location and its attributes. The fifth input file contains five sample daily movement trajectories for each individual. The sixth input file contains the birth year and death year of each human, all of which are calculated prior to running the simulation. The seventh input file specifies the rate at which visitors infected with each of the four DENV serotypes appear in the simulation on each day. The eighth input file specifies parameters that control initial conditions for serotype-specific seroprevalence in the population on the first day of the simulation due to DENV exposure prior to the timeframe of the simulation. Finally, the ninth input file specifies several time-varying model parameters on a daily basis that are driven by weather conditions and are calculated prior to running the simulation. These parameters include the incubation period of DENV in mosquitoes (i.e., extrinsic incubation period), two different biting rates for the mosquito's first bite and for subsequent bites, adult mosquito death rate, and the temporal contribution to the additive model of mosquito emergence rates.

Submodels
Movement by individual people
We adopt a submodel for movement by people described by Perkins et al. [34]. This model offers a means to simulate an individual's activity space, which is defined as both the collection of locations that a person visits as a matter of routine and a description of the proportion of time that the person spends at each of those locations. The model furthermore specifies that a person moves about those locations through time according to a continuous-time Markov process, which depends on simulated values of two key attributes of a person's connection to a location: how often they visit the location and how long they stay there during an average visit.

This submodel was fitted to data from retrospective, semi-structured interviews of residents of Iquitos. These interviews were structured in such a way as to facilitate recall of specific locations visited during specific time frames during the two weeks preceding the interview. Fitting this model to those data, Perkins et al. [34] found that location type and distance from home significantly affect a person's likelihood of visiting a location and also how often and for how long they visit. Furthermore, by accounting for the availability of locations at different distances from home depending on where a person lives (e.g., in the city center or on the periphery), the model successfully accounted for differences in movement patterns of residents living in two different neighborhoods in Iquitos. This finding is significant because it suggests that this submodel can be reasonably applied to simulated residents throughout the city and not just within the study area. The fitted movement model is also representative in the sense that interviews were conducted on a diverse group of individuals of different ages, sexes, and occupations [34].

For application to the model, we use this submodel to simulate five stochastic realizations of a daily movement trajectory for each simulated person in our synthetic population. Each trajectory

consists of a sequence of locations and what fraction of the day is spent at each location during each visit. These movement trajectories are incorporated into the model through an input file. At the beginning of each simulated day, one of these trajectories is randomly chosen and followed for that day.

Movement by mosquitoes
Each mosquito has a constant probability of 70% of staying at its current location for a given day [37]. If it moves, the location to which it moves is drawn randomly from all locations within a 50 meter radius of the mosquito's location.

Mosquito emergence
Daily emergence of mosquitoes at each location is modeled as a Poisson random variable with rate parameter $\lambda_{l,t}$ for location *l* on day *t*. We derived $\lambda_{l,t}$ by first defining the relationship

$$\frac{dN_{l,t}}{dt} = \lambda_{l,t} - \mu_t \qquad \text{(S1)}$$

between the daily rate of adult mosquito emergence $\lambda_{l,t}$, the daily rate of adult mosquito mortality μ_t, and the daily rate of change $dN_{l,t}/dt$ in the adult female mosquito population at location *l* on day *t*. For μ_t, we used values identical to those used in the simulation model, which were specific to each day *t* as a function of that day's mean temperature and were identical for all locations *l*. For $dN_{l,t}/dt$, we computed daily differences in mosquito abundance $N_{l,t}$ as estimated statistically by Reiner et al. [36]. The estimates by Reiner et al. [36] were based on an analysis of *Ae. aegypti* abundance surveys with hand-held aspirators at the level of individual residences that yielded 48,015 female *Ae. aegypti* mosquitoes captured in total between 1999 and 2011. These estimates of $N_{l,t}$ were obtained using a distributed lag nonlinear model that incorporated nonlinear effects of multiple weather variables—including mean temperature, minimum temperature, daily temperature range, precipitation, and relative humidity—at lags ranging 0-30 days. In addition to temporal variation in $N_{l,t}$ driven by these factors, the model by Reiner et al. [36] also allowed for spatial variation in $N_{l,t}$ driven by smooth splines that were estimated as part of the fitting process for that model.

An important characteristic of the mosquito abundance estimates is that the capturing method was improved in 2009 [36], meaning that the relationship between empirical estimates and mosquito densities simulated in the model could differ before and after 2009. Hence, we applied two different scaling parameters in our calculations of mosquito emergence: e_0 from 1999-2008 and e_1 from 2009-2011. One additional adjustment that we made to the emergence rate $\lambda_{l,t}$ was to multiply it by a factor to account for differences in mosquito emergence by location type as estimated through pupal surveys by Morrison et al. [47,48]. This was necessary given that the data from Reiner et al. [36] derive from data collected exclusively at residential locations, whereas our model pertains to a greater diversity of location types. To account for differences in mosquito emergence rates as a function of location type, we multiplied $\lambda_{l,t}$ by the ratio of pupae per hectare for a given location type relative to pupae per hectare for residential locations (141.5 based on the midpoint of the 122-161 range) reported by [48] and then by the ratio of the area of a given location relative to the area of an average residential location (0.0194 hectares per residence, on average). For example, for a school that is 0.25 hectares in area, we calculated its value of $\lambda_{l,t}$ as differing by a factor of 1.11 (12.2 pupae per hectare for schools / 141.5 pupae per hectare for residences x 0.25 hectares / 0.0194 hectares per residence) relative to what $\lambda_{l,t}$

would be for a residence of average size in the same location. In general, this resulted in residential locations and locations with larger areas having higher $\lambda_{l,t}$.

Mosquito death
Mosquitoes are subject to a daily mortality rate μ_t that varies from day to day as a function of mean daily temperature. To inform the relationship between temperature and μ_t, we used an adaptation of the temperature-mortality relationship estimated by Brady et al. [35]. We simplified the relatively complex functional form of the age-dependent mortality function of temperature estimated by Brady et al. using an age-independent mortality function of temperature. Following Perkins et al. [81], we calculated expected lifespan under the temperature-mortality relationship of Brady et al. across a range of temperatures and then took the reciprocal to obtain an age-independent estimate of daily adult *Ae. aegypti* mortality. Based on the temperature on the day when a simulated mosquito emerged, we used the corresponding mortality rate on that day to simulate a time of death some days later consistent with an exponentially distributed lifespan.

Mosquito blood-feeding
Upon emergence, a mosquito refrains from attempting to bite for an exponentially distributed period of time, with the average duration of that period set to a function of temperature described by Focks et al. [38]. Following the first and all subsequent blood-meals, a mosquito refrains from attempting to bite for another exponentially distributed period of time but with an average duration set to a different function of temperature described by Otero et al. [39]. In general, this captures the tendency for a longer period of time between emergence and the first blood-meal than between subsequent blood-meals. With the time of the attempted bite determined, the mosquito selects a particular person on whom to blood-feed by taking a random draw from a categorical distribution informed by a function of the body size of all people present at the location at that time [32]. If no people are present, the mosquito is assumed to either find another source of blood or wait until its next scheduled blood meal.

Infection of susceptible people by infectious mosquitoes
After a bite by an infectious mosquito, a susceptible human becomes infected with probability 0.9 (following the assumption of another modeling study [43]). Although this probability may in fact be less than 0.9, we fixed its value given that it was assumed to likely be unidentifiable through our model fitting process given an expected trade-off between values of this probability and the multiplier on mosquito densities estimated by Reiner et al. [36]. Infected people develop symptomatic disease with probabilities dependent on the number of previous exposures to DENV that they have experienced: 0.18 for those with no previous exposures, 0.24 for those with one previous exposure, and 0.14 for those with two or three previous exposures [20,42].

Infection of susceptible mosquitoes by infectious people
When blood-feeding on an infectious person, a susceptible mosquito is infected with a probability determined by the person's infectiousness. A person's infectiousness on a given day since infection was derived from an analysis by Nishiura and Halstead [44] of data on human infectiousness originally published by Sabin [79]. We approximated the infectiousness data as presented by Nishiura and Halstead with the function

$$1.01\, e^{-0.278\,(t\,-\,(IIP\,-\,0.288))^{\,2}}, \qquad (S2)$$

where *t* is the number of days since the human was infected by a mosquito and *IIP* is the length of the incubation period in the human (intrinsic incubation period). Each person is assigned an

IIP by taking a random draw from a lognormal distribution fitted by Chan and Johansson [41]. To model whether successful infection occurred, a Bernoulli trial is performed with a probability of infection equal to that person's infectiousness on that day. Upon infection, mosquitoes enter a period of latent infection for a period of time drawn from an empirically estimated lognormal distribution with a mean that depends on mean daily temperature and results in an average incubation period of 6.5 days at 30 °C [41]. Upon completion of the latent period, a mosquito becomes infectious and remains so for the remainder of its life. If a mosquito is exposed a second time after becoming infected once, the latter exposure has no impact on the outcome of the initial infection (e.g., an individual mosquito can only ever be infected by a single DENV serotype).

Introduction of dengue virus into the simulated population
To seed DENV infections into the population, we introduced individual humans into the population infected with a given serotype of DENV as temporary inhabitants for the duration of their infection. We chose to seed infections with temporary inhabitants, rather than residents, to minimize the impact of DENV introduction on population susceptibility among residents. The number of temporary inhabitants infected with a given serotype introduced on a given day was determined in any given simulation by drawing a number of such individuals from a Poisson distribution with rate parameter described by a cubic B-spline function. Because these calculations occurred in R and were later fed into the C++ model with input files, we represented the spline function using the fda package [82] in R. This function was described by parameters $x_{i,j}$ (modal timing of importation in a year) and $y_{i,j}$ (constant for scaling the magnitude of importation in a year), where *i* refers to the serotype and *j* refers to the year.

Changes in the immune status of individual people
Following infection with DENV, it is generally accepted that there is a temporary period of heterologous immunity. We implement the best-fit parameterization of a published model [45] whereby individuals are completely protected following an infection, but the duration of this protection for each individual is drawn independently from an exponential distribution with a mean of 686 days. Because Reich et al. indicated that the support for that particular parameterization is only moderately strong, we also explored the sensitivity of the model's behavior to alternative assumptions about how the duration of heterologous immunity is distributed across different people (exponential distribution or fixed duration) and the average duration of heterologous immunity (180, 360, or 686 days). Under all of these scenarios, individuals resume their susceptibility to serotypes to which they have no prior exposure at the end of their period of heterologous immunity. Although there is emerging evidence that homologous immunity (i.e., immunity to a DENV serotype to which a person has been exposed) may not be complete and lifelong in all instances [83], we followed the prevailing assumption in DENV biology that homologous immunity is fully protective against subsequent infection with a DENV serotype to which a person has been exposed for the remainder of that person's life.

Demographic changes in the human population
We simulated demographic changes in the human population consistent with past estimates and future projections of demography for Peru from the United Nations [31]. We applied numbers appropriate for the period of 2000-2010 for simulated demographic processes during the period of model calibration and for 2011-2031 for the period of vaccine impact projections. On any given day of the simulation, death was simulated for each individual according to a Bernoulli trial with a probability informed by age- and year-specific survival probabilities reported by the UN in five-year increments for both time and age, with the exception of one-year increments for ages 0 and 1. Births were simulated based on UN estimates of births per 1,000

population in five-year increments extrapolated to a daily rate of birth appropriate for the size of the simulated population on a given day to obtain the expected number of births on that day. The realized number of births on that day was then drawn as a random number from a Poisson distribution with rate parameter equal to the expected number of births. Newborn children were assigned to mothers of ages ranging 15-49, with the probability of a given woman being the mother proportional to age-specific fertility rates reported by the UN in five-year increments of age and time. Children are born with a normally distributed body surface area that grows linearly to a normally distributed adult body surface area that is attained at a threshold age. We fitted sex-specific parameters for these body growth parameters using biometric data collected during a study of heterogeneous biting in Iquitos [32]. These parameters include body size at birth for males (normal with mean 0.31 and s.d. 0.30) and females (normal with mean 0.31 and s.d. 0.18), final adult body sizes for males (normal with mean 1.71 and s.d. 0.30) and females (normal with mean 1.51 and s.d. 0.24), and the ages at which adult body sizes were obtained for males (18.65 years) and females (16.52 years). These parameters were sufficient to linearly interpolate the body size for a given individual between its body sizes at birth and in adulthood.

Vaccination

We distribute a vaccine—either resembling CYD-TDV or a more generic vaccine—to 80% of children on their ninth birthday, and we assume that 100% of vaccinees comply with the full vaccination schedule of three doses over 12 months. We assume 80% initial compliance and 100% follow-up compliance in accordance with a previous modeling assessment of the public health impact and cost effectiveness of CYD-TDV [14], and we apply the vaccine at age nine consistent with recommendations for CYD-TDV [84]. For the CYD-TDV vaccine, we assume that the efficacy of the vaccine tracks the relationship in eqn. (2) as an individual ages and in the event of a change in serostatus from negative to positive. For both vaccine types, we specify the portion of overall efficacy in VE_{dis} that derives from protection against infection versus protection against disease conditional on infection using a free parameter p to specify the relative reduction in infection as $RR_{inf|exp} = (1 - VE_{dis})^{p}$ and relative reduction in disease conditional on infection as $RR_{dis|inf} = (1 - VE_{dis})^{1-p}$.
