# Supplementary material for: An agent-based model of dengue virus transmission shows how uncertainty about breakthrough infections influences vaccination impact projections
Source: PLoS Comput Biol. 2019 Mar 20;15(3):e1006710. doi: 10.1371/journal.pcbi.1006710 (PMC6443188; doi:10.1371/journal.pcbi.1006710)
Supplement: S2 Text — (PDF) [file pcbi.1006710.s002.pdf]

S2 Text. Impact of number of exposures on the interpretation of vaccine efficacy.

for

An agent-based model of dengue virus transmission shows how uncertainty about breakthrough infections influences vaccination impact projections

T. Alex Perkins[1,2,$,*], Robert C. Reiner[2,3,$], Guido España[1,$], Quirine A. ten Bosch[1], Amit Verma[4], Kelly A. Liebman[5], Valerie A. Paz-Soldan[6], John P. Elder[7], Amy C. Morrison[5], Steven T. Stoddard[7], Uriel Kitron[2,8], Gonzalo M. Vazquez-Prokopec[2,8], Thomas W. Scott[2,5], David L. Smith[2,9]

[1] Department of Biological Sciences and Eck Institute for Global Health, University of Notre Dame, Notre Dame, IN, USA
[2] Fogarty International Center, National Institutes of Health, Bethesda, MD, USA
[3] Department of Epidemiology and Biostatistics, Indiana University, Bloomington, IN, USA
[4] Center for Disease Dynamics, Economics, and Policy, Washington, DC, USA
[5] Department of Entomology and Nematology, University of California, Davis, CA, USA
[6] Department of Global Community Health and Behavioral Sciences, Tulane University School of Public Health and Tropical Medicine, New Orleans, LA, USA
[7] Institute for Behavioral and Community Health, Graduate School of Public Health, San Diego State University, San Diego, CA, USA
[8] Department of Environmental Sciences, Emory University, Atlanta, GA, USA
[9] Institute for Health Metrics and Evaluation, University of Washington, Seattle, WA, USA

$ Denotes equal contributions
* Author for correspondence: taperkins@nd.edu

Impact of number of exposures on the interpretation of vaccine efficacy

For a leaky vaccine, it is often the case that vaccine efficacy measured during a clinical trial, VE, and per-exposure reduction in the probability of transmission, 1-θ, have different values. In fact, these values are different any time trial participants are exposed more than once. On the one hand, θ is not typically estimated in analyses of dengue vaccine trial results due to the infeasibility of counting the number of exposures that each individual experiences. On the other hand, any modeling analysis of vaccination impact requires estimates of θ given that modeling impact outside the context of a trial is the primary purpose of such an analysis.

Here, we explore the magnitude of the possible difference between VE and 1-θ in the context of phase-III CYD-TDV vaccine trials as summarized by Hadinegoro et al. [16]. To do so, we apply logic presented by Halloran et al. [56] for relating VE and 1-θ in the event of a fixed number of exposures *n*. To apply that result in the context of a variable number of exposures, we assumed an equal distribution of *n* in vaccinated and unvaccinated arms of the trial such that $n \sim \text{Poisson}(FOI\ \Delta t)$, where *FOI* is the force of infection and Δt is the duration of the trial.

For a fixed number of exposures *n* and a per-exposure probability of transmission p_0, Halloran et al. [56] showed that

$$VE = 1 - \frac{1-(1-\theta p_0)^n}{1-(1-p_0)^n}.$$

Under our assumption that *n* follows a Poisson distribution with rate $FOI\Delta t$, the probability that a randomly selected trial participant experiences *n* exposures is

$$\Pr(n) = \frac{(FOI\Delta t)^n e^{-FOI\Delta t}}{n!}.$$

Variability in *n* can be incorporated into the result by Halloran et al. [56] by applying the law of total probability separately in the numerator and denominator of the rightmost term in the first equation, yielding

$$VE = 1 - \frac{\sum_n (1-(1-\theta p_0)^n)\Pr(n)}{\sum_n (1-(1-p_0)^n)\Pr(n)}.$$

To explore the relationship between VE and 1-θ, we numerically solved for 1-θ given VE and $FOI\Delta t$ in R across a range of values of those quantities. For this analysis, we assumed that p_0=1.0. Differences between VE and 1-θ were greatest for intermediate VE (S40 Fig.), with the maximum percentage difference between VE and 1-θ ranging 4.8-13.4% between values of $FOI\Delta t$ ranging 0.1-0.3. The force of infection experienced in phase-III CYD-TDV trials was heterogeneous across sites and over time, but values of $FOI\Delta t$ over the course of those one-year trials generally were observed to be in this range of 0.1-0.3 [85]. Altogether, this suggests that θ may be slightly lower than VE, but likely only by 5% or less. Based in part on this result that VE and 1-θ are not likely to differ by much, we chose to approximate 1-θ with VE in the main text. We also made this choice due to the fact that our primary objective in the main text was to make a qualitative point about the implications of different types of uncertainty in VE for vaccine impact projections rather than to make a quantitatively accurate vaccine impact projection.
