# Supplementary material for: An agent-based model of dengue virus transmission shows how uncertainty about breakthrough infections influences vaccination impact projections
Source: PLoS Comput Biol. 2019 Mar 20;15(3):e1006710. doi: 10.1371/journal.pcbi.1006710 (PMC6443188; doi:10.1371/journal.pcbi.1006710)
Supplement: S3 Text — (PDF) [file pcbi.1006710.s003.pdf]

S3 Text. Supplemental references.

for

An agent-based model of dengue virus transmission shows how uncertainty about breakthrough infections influences vaccination impact projections

T. Alex Perkins[1,2,$,*], Robert C. Reiner[2,3,$], Guido España[1,$], Quirine A. ten Bosch[1], Amit Verma[4], Kelly A. Liebman[5], Valerie A. Paz-Soldan[6], John P. Elder[7], Amy C. Morrison[5], Steven T. Stoddard[7], Uriel Kitron[2,8], Gonzalo M. Vazquez-Prokopec[2,8], Thomas W. Scott[2,5], David L. Smith[2,9]

[1] Department of Biological Sciences and Eck Institute for Global Health, University of Notre Dame, Notre Dame, IN, USA
[2] Fogarty International Center, National Institutes of Health, Bethesda, MD, USA
[3] Department of Epidemiology and Biostatistics, Indiana University, Bloomington, IN, USA
[4] Center for Disease Dynamics, Economics, and Policy, Washington, DC, USA
[5] Department of Entomology and Nematology, University of California, Davis, CA, USA
[6] Department of Global Community Health and Behavioral Sciences, Tulane University School of Public Health and Tropical Medicine, New Orleans, LA, USA
[7] Institute for Behavioral and Community Health, Graduate School of Public Health, San Diego State University, San Diego, CA, USA
[8] Department of Environmental Sciences, Emory University, Atlanta, GA, USA
[9] Institute for Health Metrics and Evaluation, University of Washington, Seattle, WA, USA

$ Denotes equal contributions
* Author for correspondence: taperkins@nd.edu

77. Gillespie DT. Exact stochastic simulation of coupled chemical reactions. J Phys Chem. 1977;81: 2340–2361.

78. Glass K, Xia Y, Grenfell BT. Interpreting time-series analyses for continuous-time biological models—measles as a case study. J Theor Biol. 2003;223: 19–25.

79. Sabin AB. Research on dengue during World War II. Am J Trop Med Hyg. 1952;1: 30–50.

80. UNdata [Internet]. [cited 30 Apr 2016]. Available: http://data.un.org

81. Perkins TA, Siraj AS, Ruktanonchai CW, Kraemer MUG, Tatem AJ. Model-based projections of Zika virus infections in childbearing women in the Americas. Nat Microbiol. 2016;1: 16126.

82. Ramsay JO, Wickham H, Graves S, Hooker G. fda: Functional Data Analysis. R Package Version 2.4.8. 2018. Available: https://CRAN.R-project.org/package=fda

83. Forshey BM, Reiner RC, Olkowski S, Morrison AC, Espinoza A, Long KC, et al. Incomplete Protection against Dengue Virus Type 2 Re-infection in Peru. PLoS Negl Trop Dis. 2016;10: e0004398.

84. WHO | SAGE meeting of April 2016. World Health Organization; 2016; Available: http://www.who.int/immunization/sage/meetings/2016/april/en/

85. Coudeville L, Baurin N, Vergu E. Estimation of parameters related to vaccine efficacy and dengue transmission from two large phase III studies. Vaccine. 2016;34: 6417-6425.
