# Supplementary figures and images for: An agent-based model of dengue virus transmission shows how uncertainty about breakthrough infections influences vaccination impact projections
Source: PLoS Comput Biol. 2019 Mar 20;15(3):e1006710. doi: 10.1371/journal.pcbi.1006710 (PMC6443188; doi:10.1371/journal.pcbi.1006710)

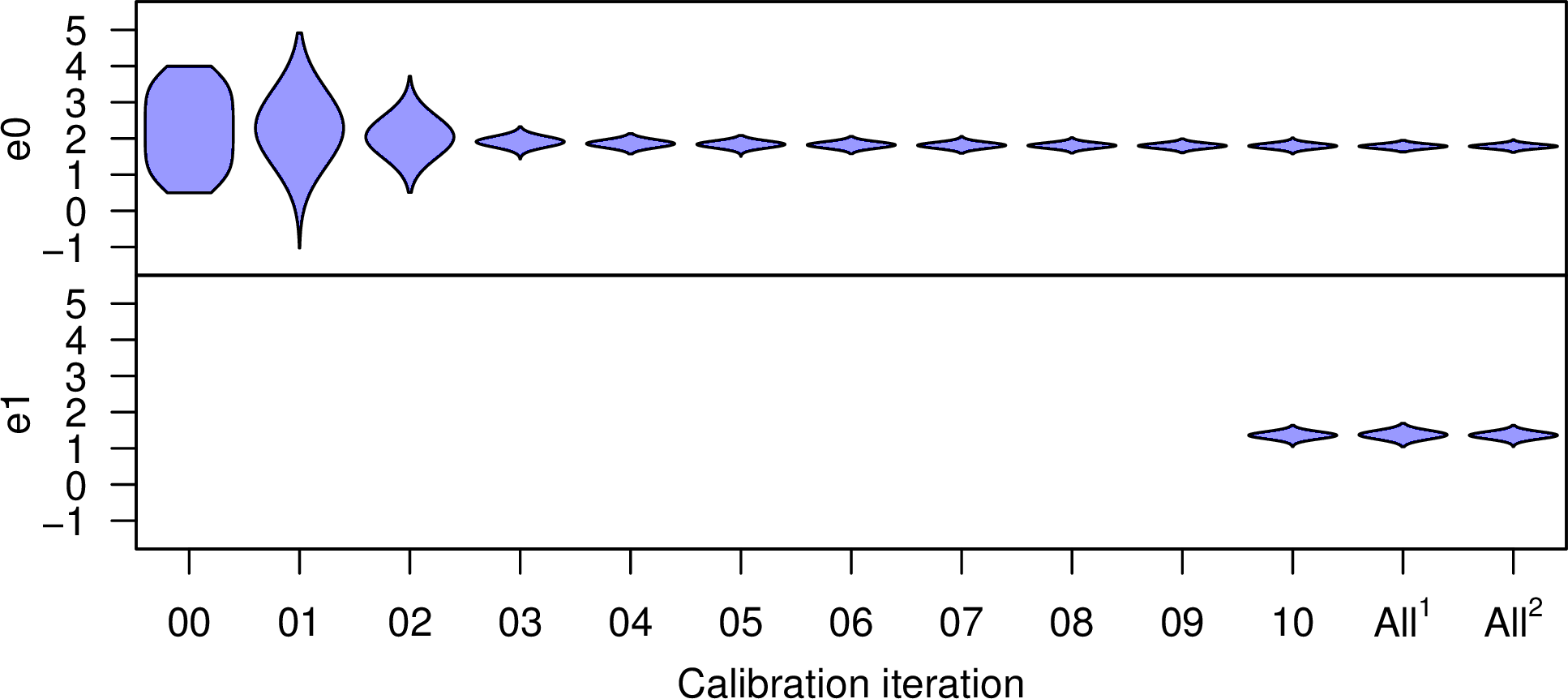

Supplement: S1 Fig — Calibration iteration refers to the iteration in the calibration process to which these distributions apply, with labels corresponding to the years up to which the calibration applies (e.g., 00 corresponds to 2000) or to calibration iterations involving all data (i.e., All1 and All2). Two parameters were used due to switch in data collection methods used in empirical work underlying our description of mosquito population dynamics. (TIF) [file pcbi.1006710.s004.tif]

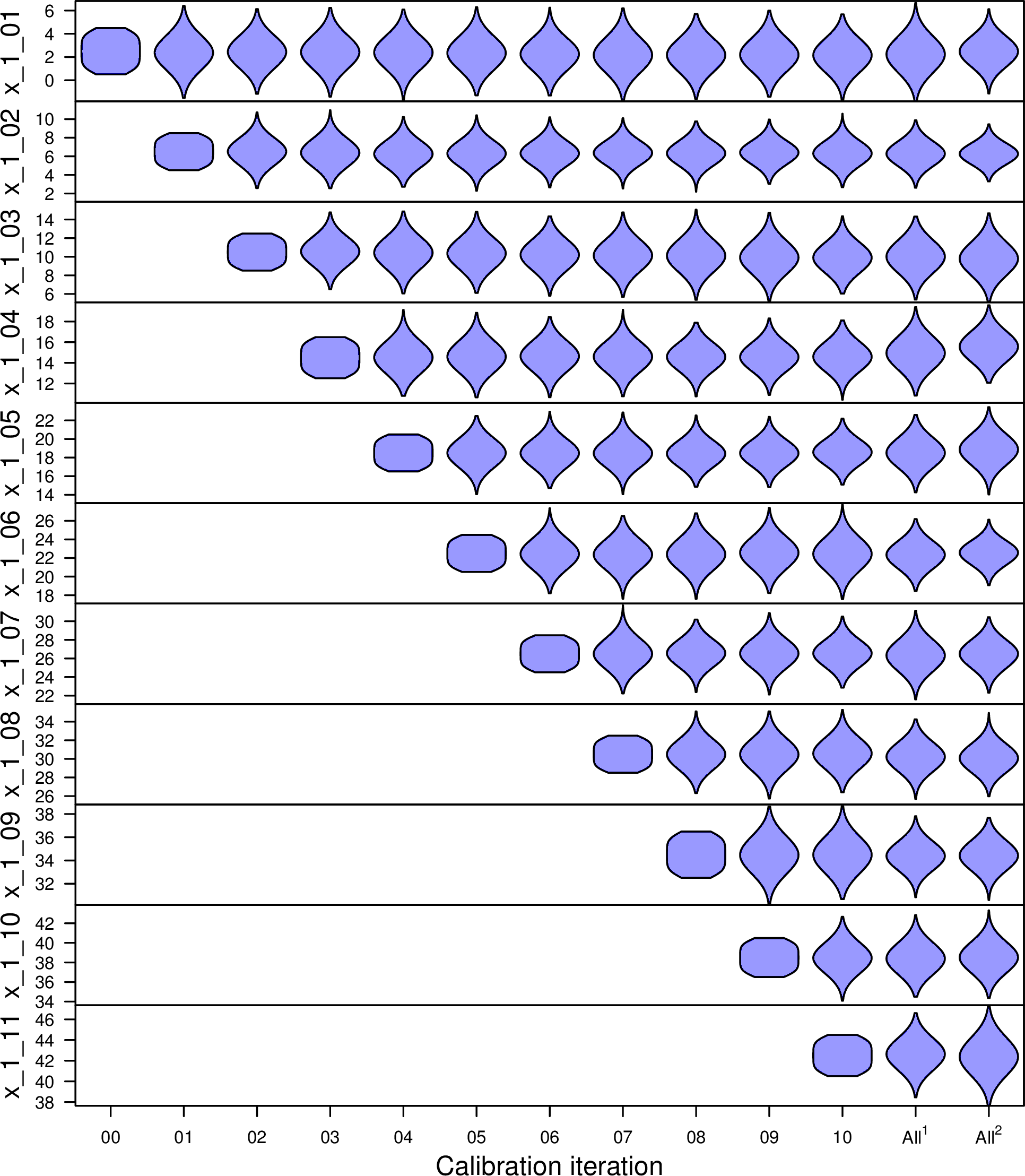

Supplement: S2 Fig — Calibration iteration refers to the iteration in the calibration process to which these distributions apply, with labels corresponding to the years up to which the calibration applies (e.g., 00 corresponds to 2000) or to calibration iterations involving all data (i.e., All1 and All2). (TIF) [file pcbi.1006710.s005.tif]

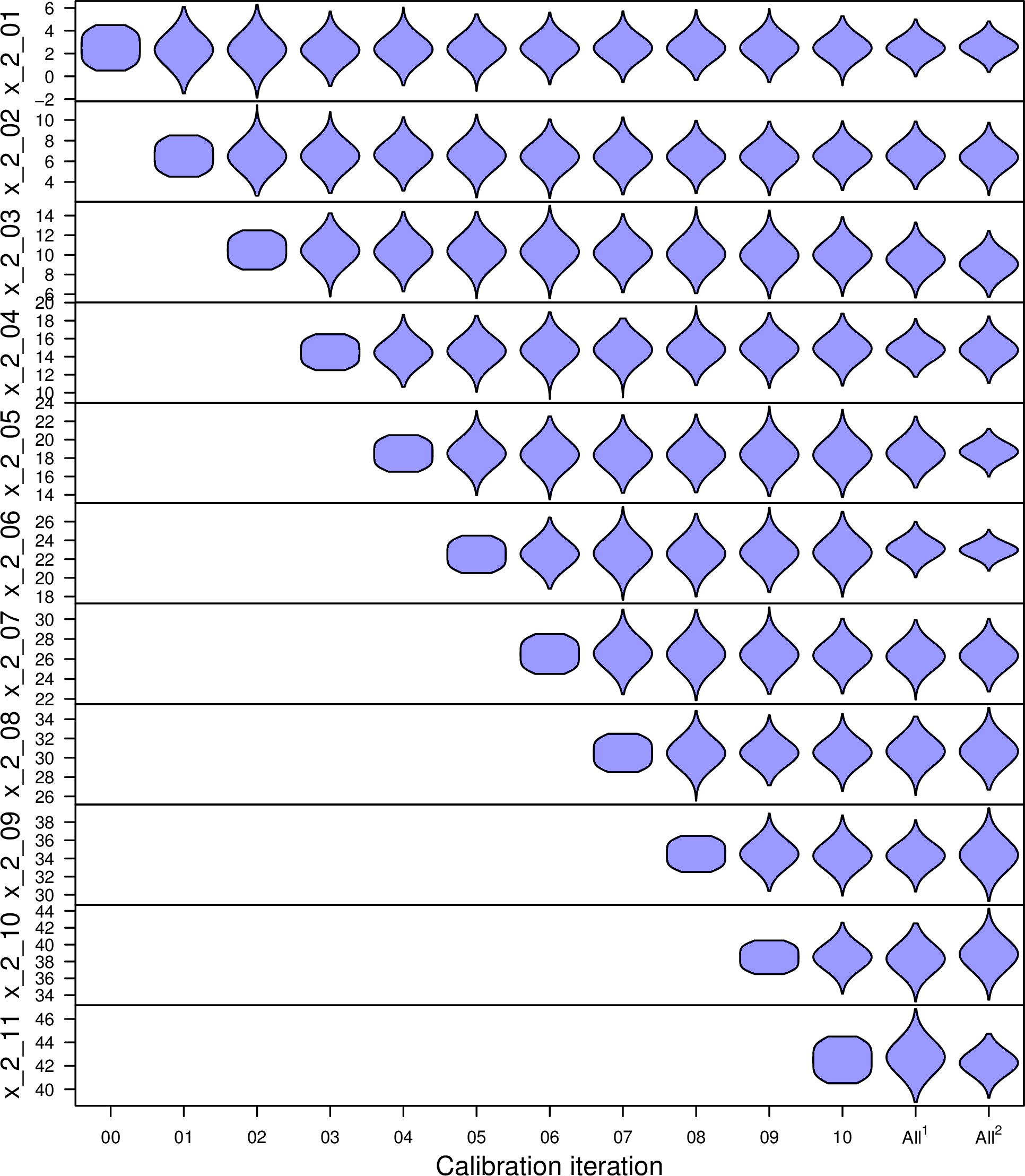

Supplement: S3 Fig — Calibration iteration refers to the iteration in the calibration process to which these distributions apply, with labels corresponding to the years up to which the calibration applies (e.g., 00 corresponds to 2000) or to calibration iterations involving all data (i.e., All1 and All2). (TIF) [file pcbi.1006710.s006.tif]

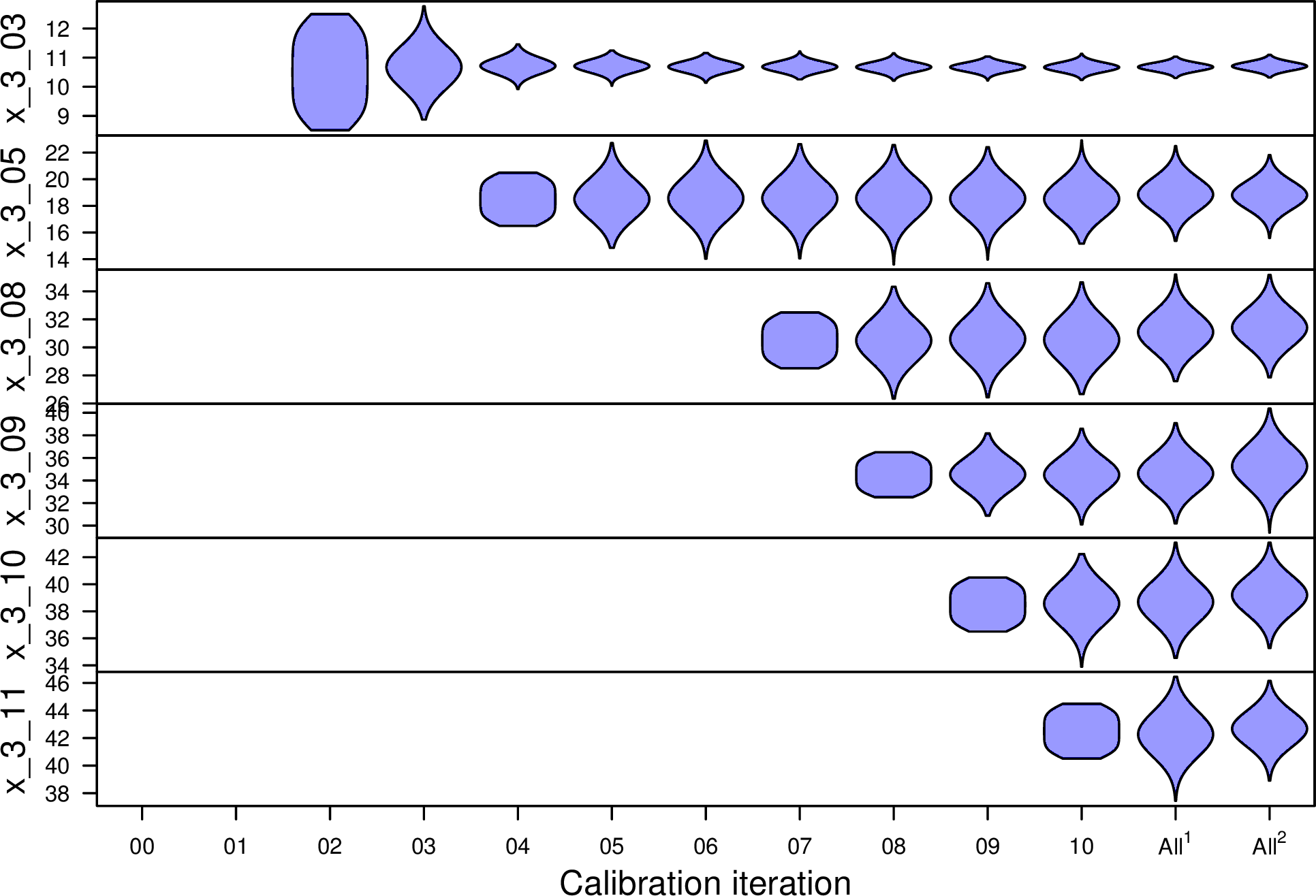

Supplement: S4 Fig — Calibration iteration refers to the iteration in the calibration process to which these distributions apply, with labels corresponding to the years up to which the calibration applies (e.g., 00 corresponds to 2000) or to calibration iterations involving all data (i.e., All1 and All2). (TIF) [file pcbi.1006710.s007.tif]

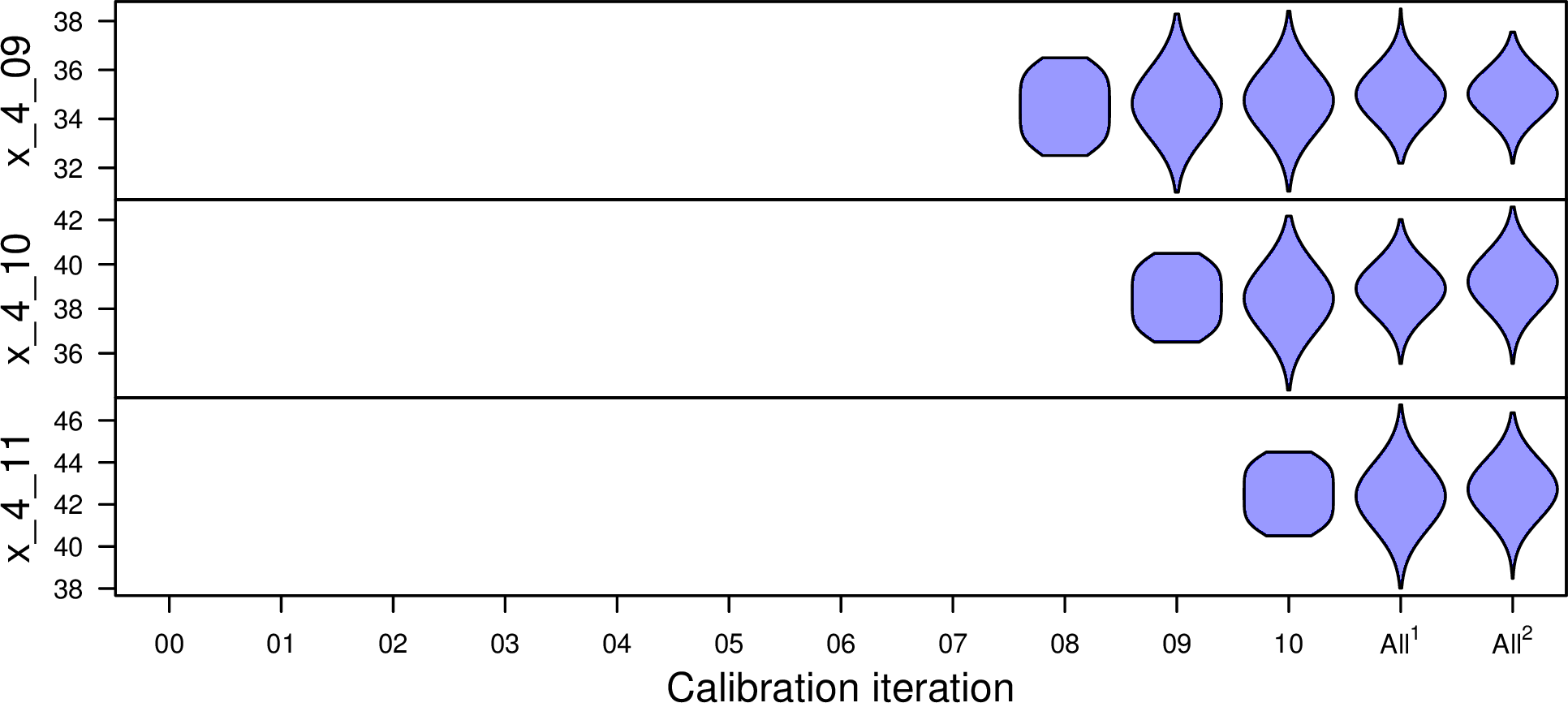

Supplement: S5 Fig — Calibration iteration refers to the iteration in the calibration process to which these distributions apply, with labels corresponding to the years up to which the calibration applies (e.g., 00 corresponds to 2000) or to calibration iterations involving all data (i.e., All1 and All2). No DENV-3 importation was simulated in years in which DENV-3 was not observed empirically in Iquitos. (TIF) [file pcbi.1006710.s008.tif]

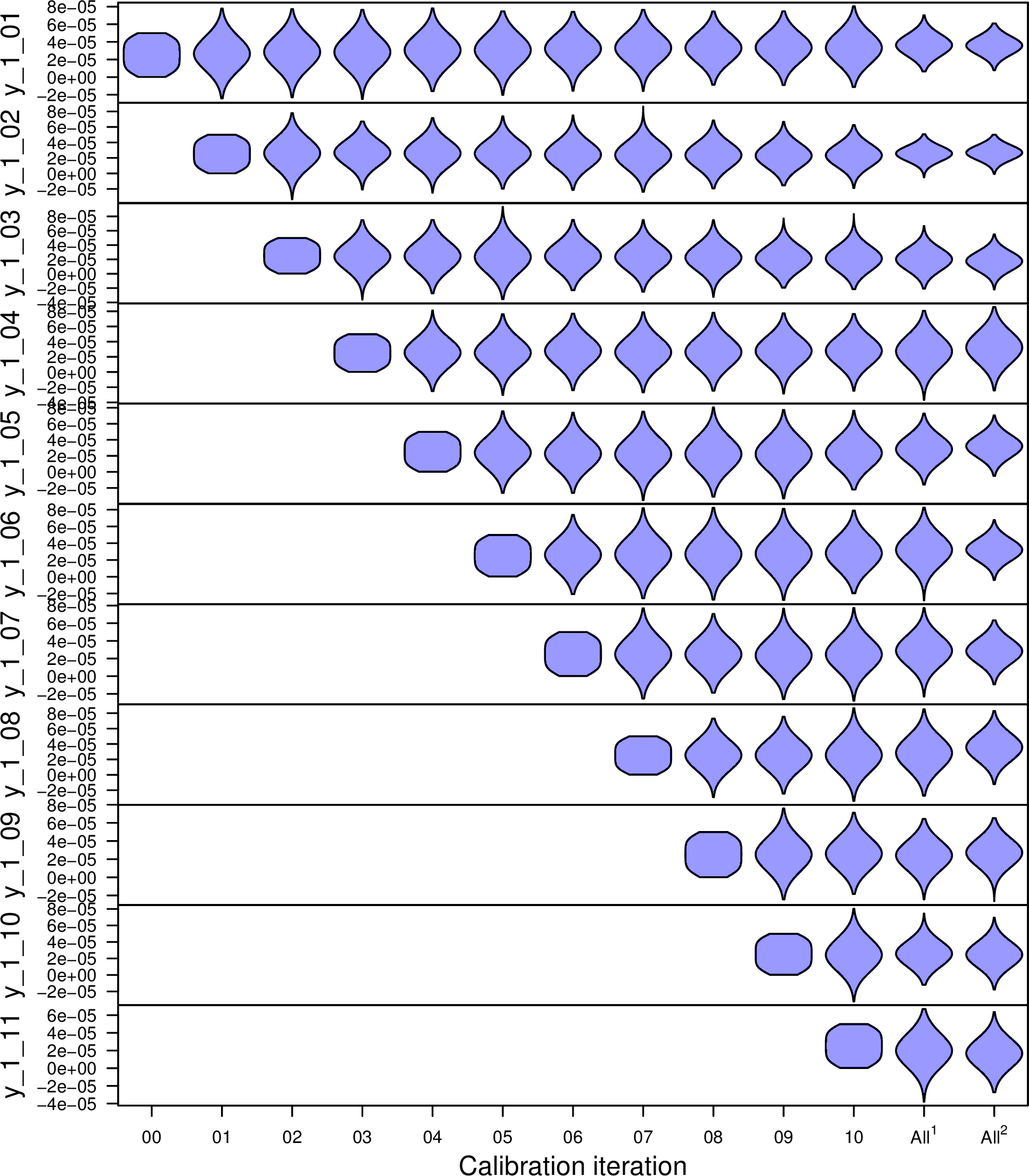

Supplement: S6 Fig — Calibration iteration refers to the iteration in the calibration process to which these distributions apply, with labels corresponding to the years up to which the calibration applies (e.g., 00 corresponds to 2000) or to calibration iterations involving all data (i.e., All1 and All2). No DENV-4 importation was simulated in years in which DENV-4 was not observed empirically in Iquitos. (TIF) [file pcbi.1006710.s009.tif]

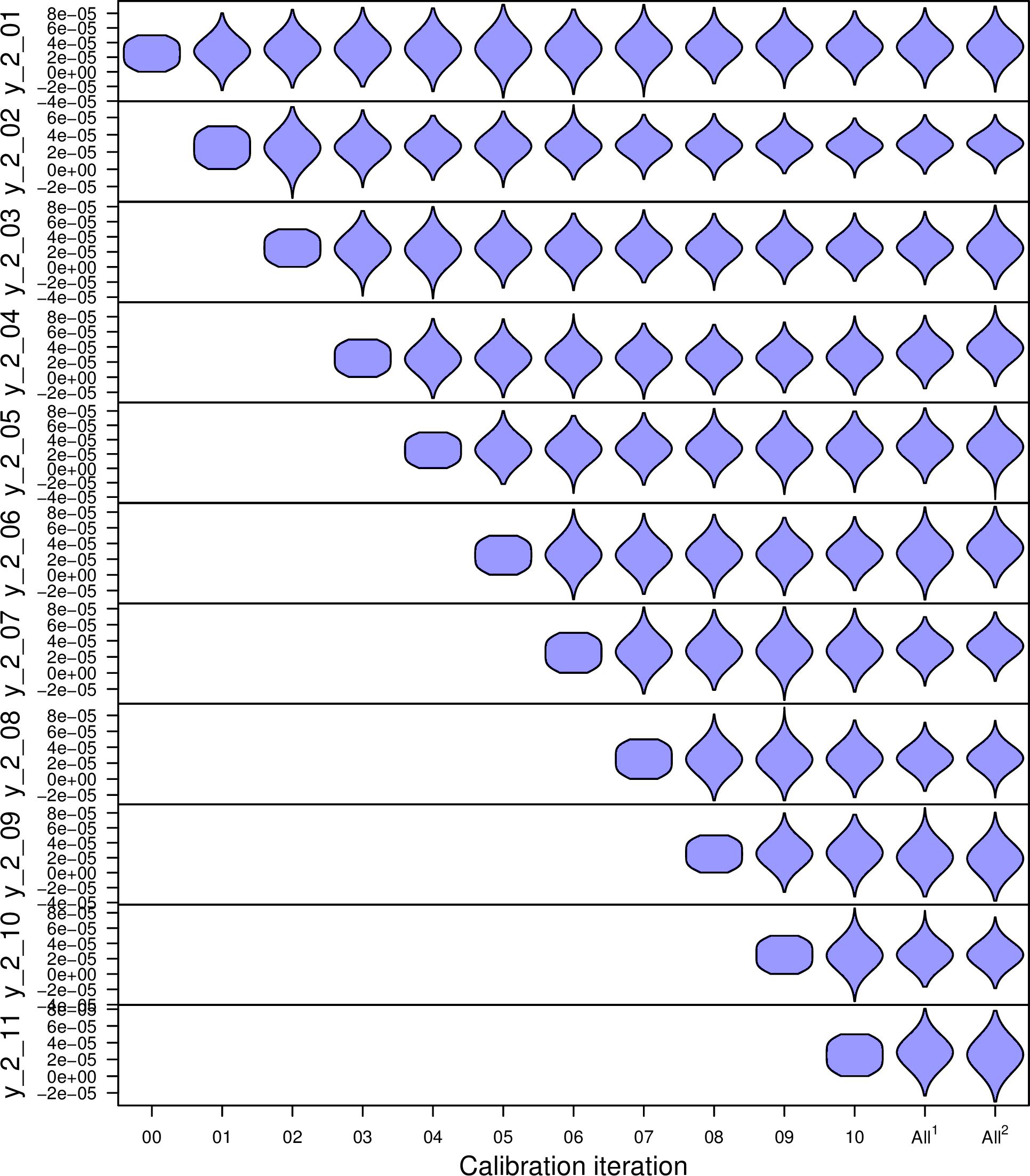

Supplement: S7 Fig — Calibration iteration refers to the iteration in the calibration process to which these distributions apply, with labels corresponding to the years up to which the calibration applies (e.g., 00 corresponds to 2000) or to calibration iterations involving all data (i.e., All1 and All2). (TIF) [file pcbi.1006710.s010.tif]

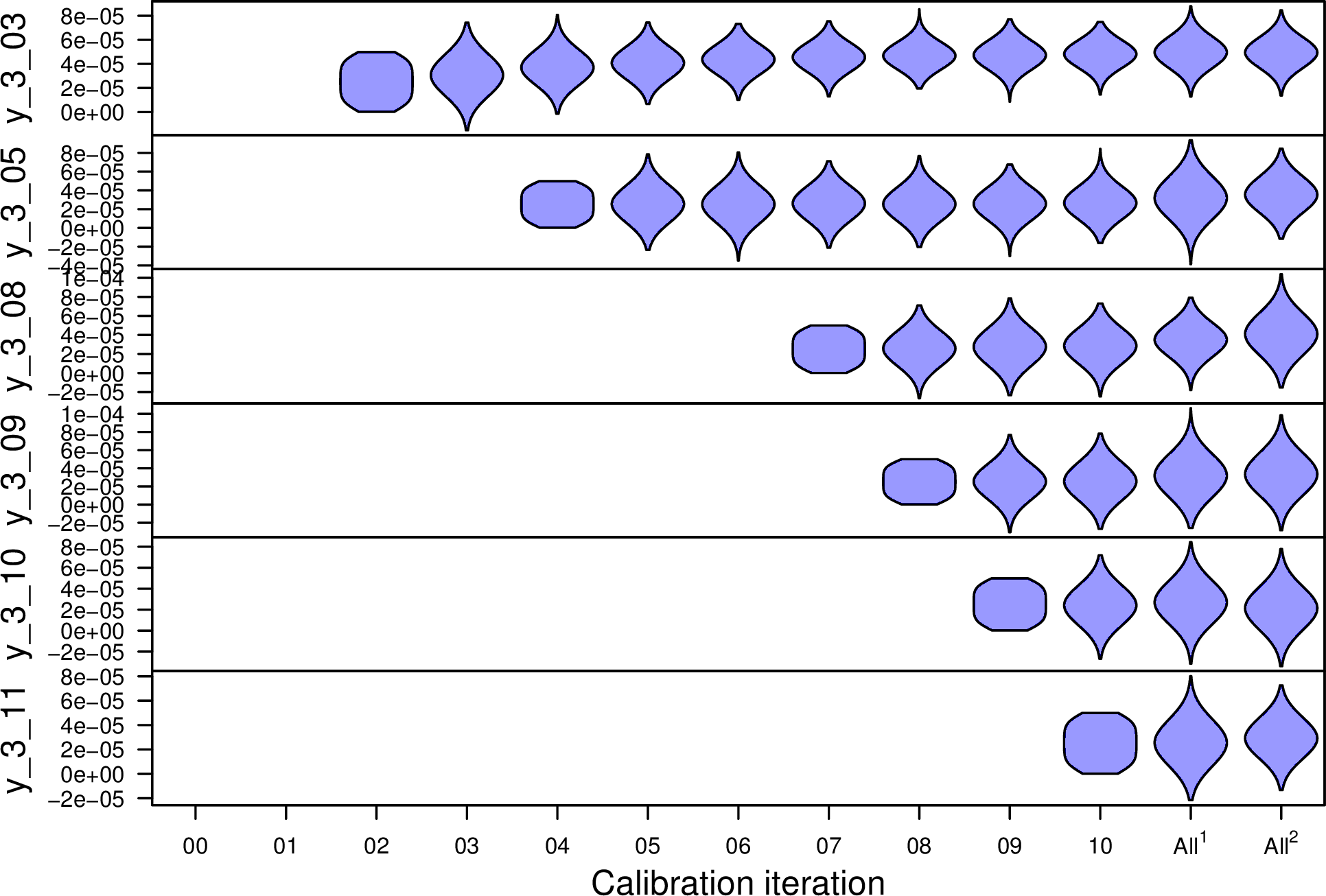

Supplement: S8 Fig — Calibration iteration refers to the iteration in the calibration process to which these distributions apply, with labels corresponding to the years up to which the calibration applies (e.g., 00 corresponds to 2000) or to calibration iterations involving all data (i.e., All1 and All2). No DENV-3 importation was simulated in years in which DENV-3 was not observed empirically in Iquitos. (TIF) [file pcbi.1006710.s011.tif]

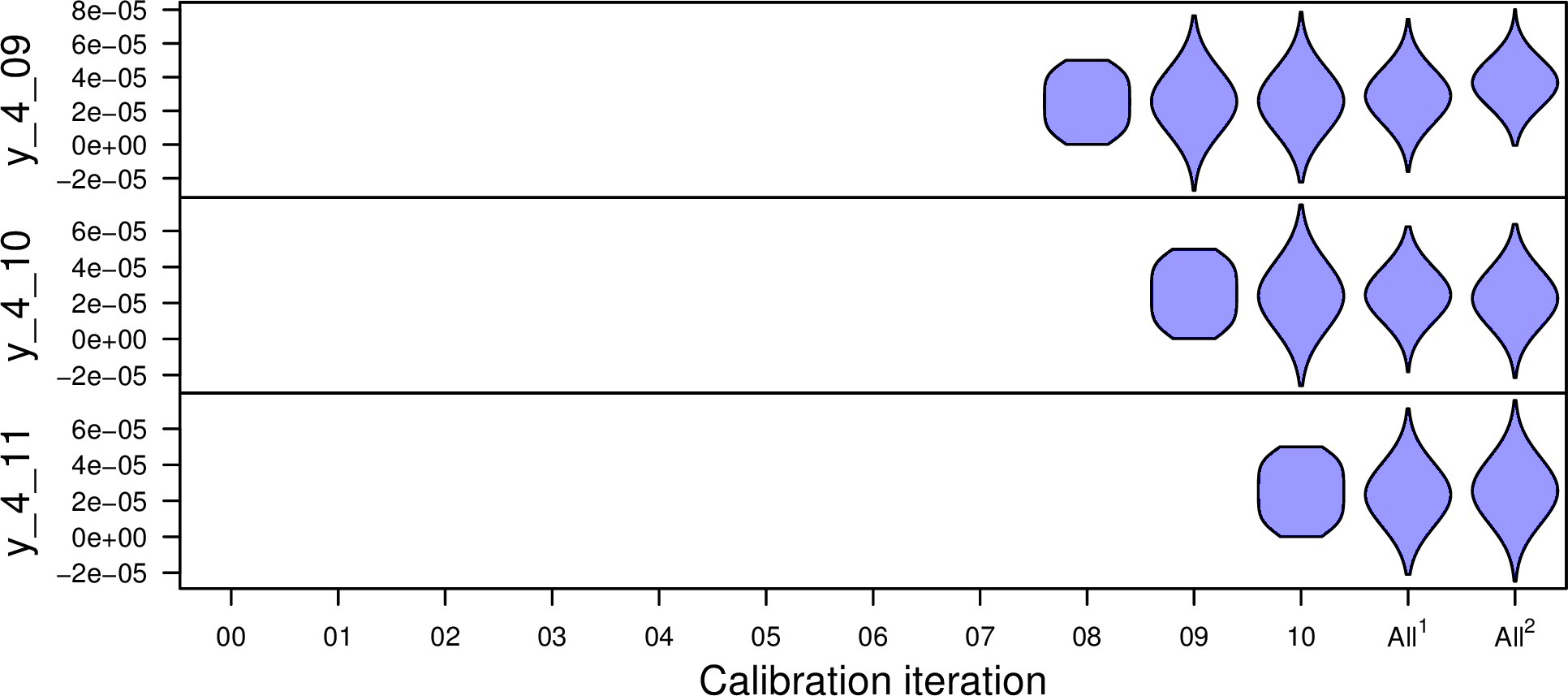

Supplement: S9 Fig — Calibration iteration refers to the iteration in the calibration process to which these distributions apply, with labels corresponding to the years up to which the calibration applies (e.g., 00 corresponds to 2000) or to calibration iterations involving all data (i.e., All1 and All2). No DENV-4 importation was simulated in years in which DENV-4 was not observed empirically in Iquitos. (TIF) [file pcbi.1006710.s012.tif]

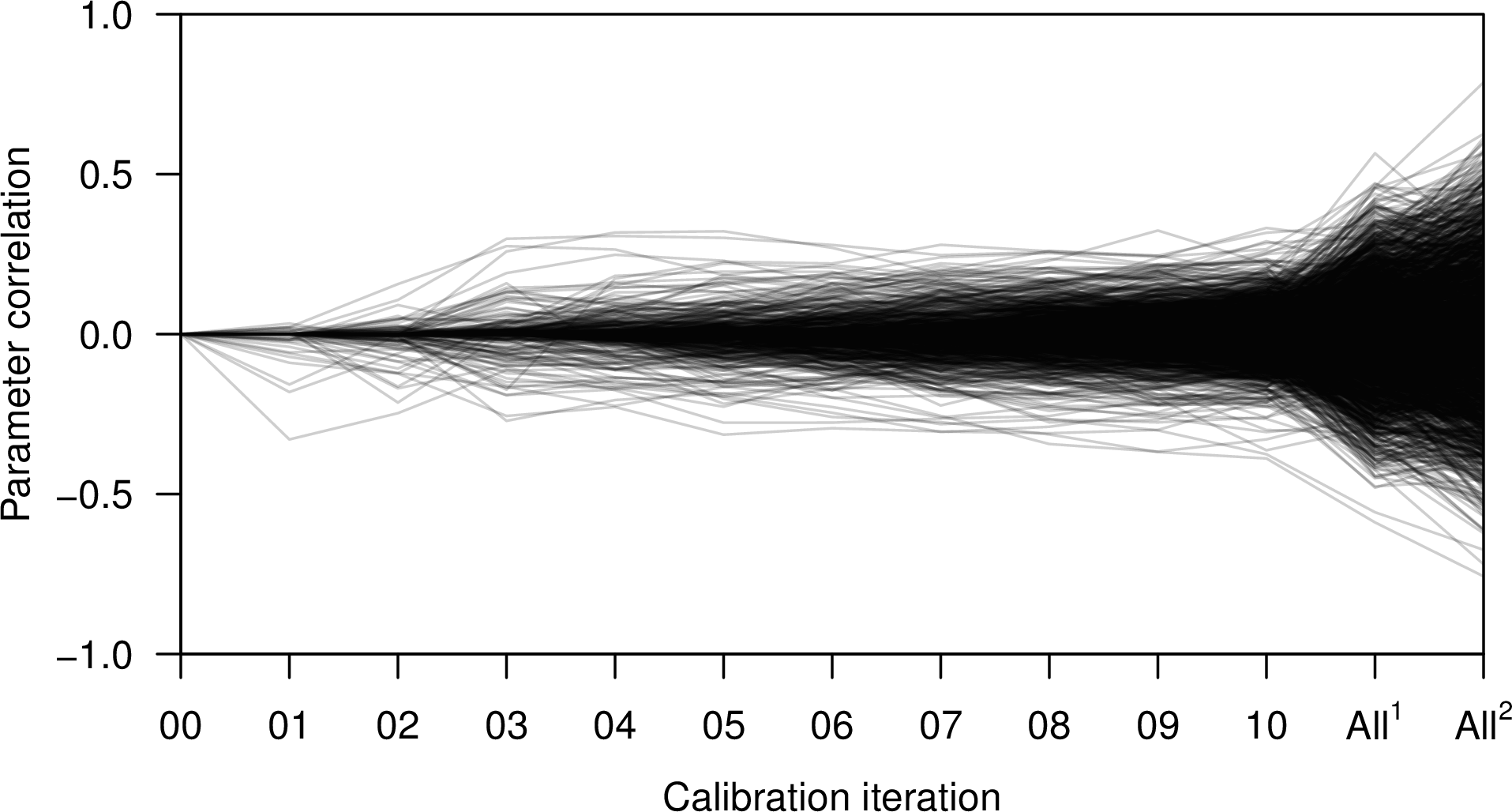

Supplement: S10 Fig — Calibration iteration refers to the iteration in the calibration process to which these distributions apply, with labels corresponding to the years up to which the calibration applies (e.g., 00 corresponds to 2000) or to calibration iterations involving all data (i.e., All1 and All2). Each line indicates the trajectory of the correlation between a given pair of parameters over the course of the calibration process. (TIF) [file pcbi.1006710.s013.tif]

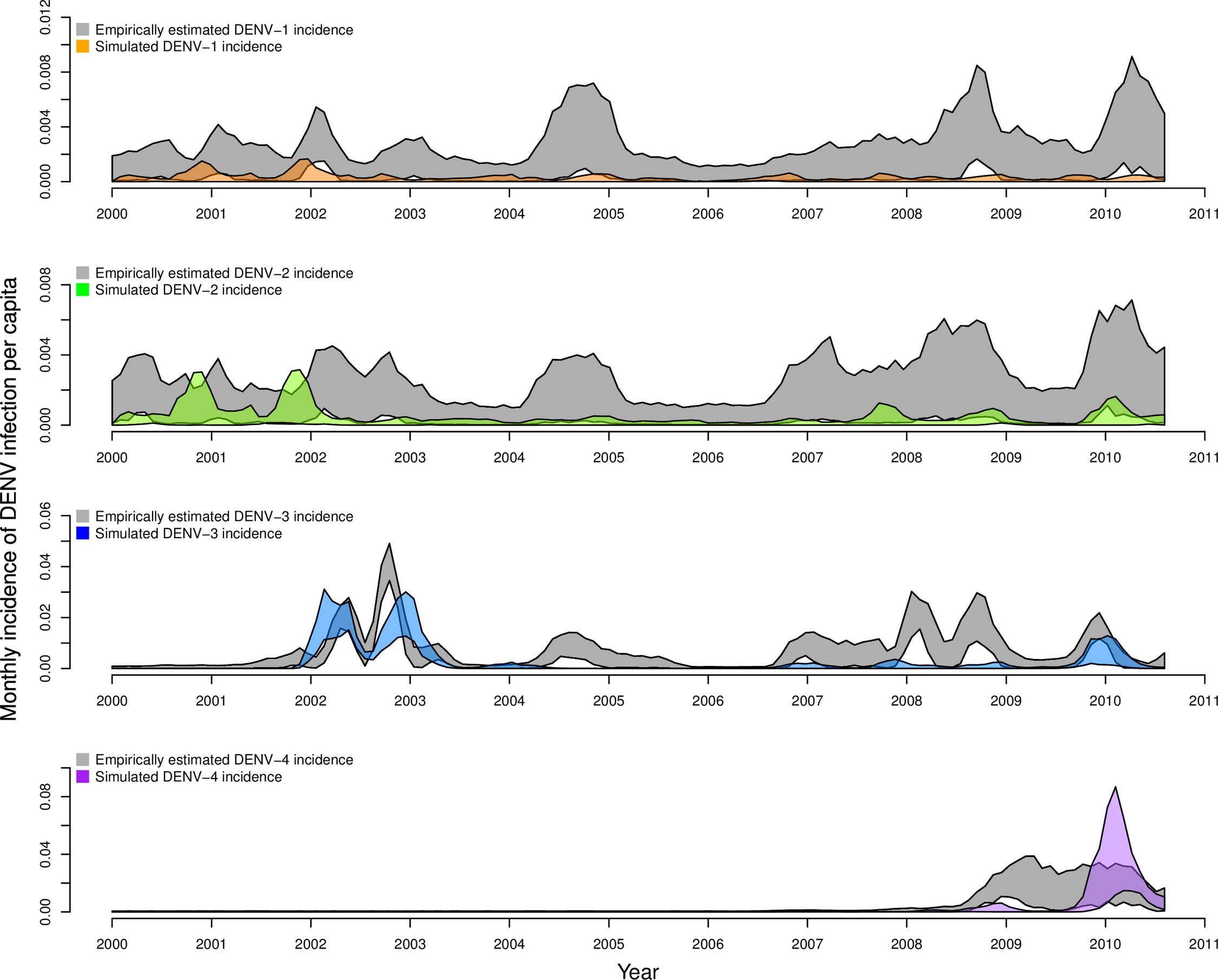

Supplement: S11 Fig — Bands show the range of values in which 95% of simulated values lie for a given serotype in a given month. These values were obtained under the assumption that the net infectiousness of asymptomatic infections is half that of symptomatic infections. Other assumptions followed the default set of assumptions. (TIF) [file pcbi.1006710.s014.tif]

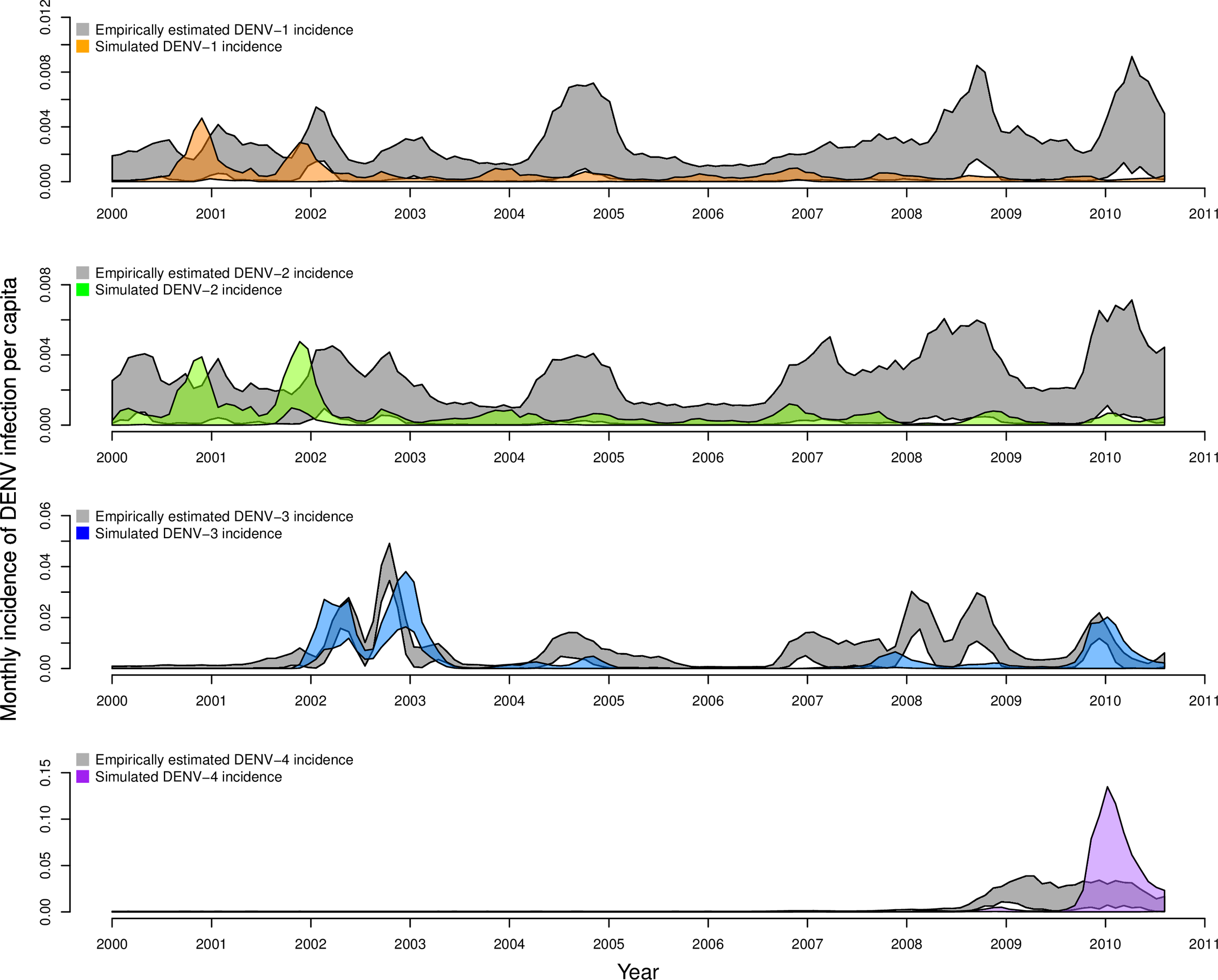

Supplement: S12 Fig — Bands show the range of values in which 95% of simulated values lie for a given serotype in a given month. These values were obtained under the assumption that the average duration of temporary cross-immunity is 180 days. Other assumptions followed the default set of assumptions. (TIF) [file pcbi.1006710.s015.tif]

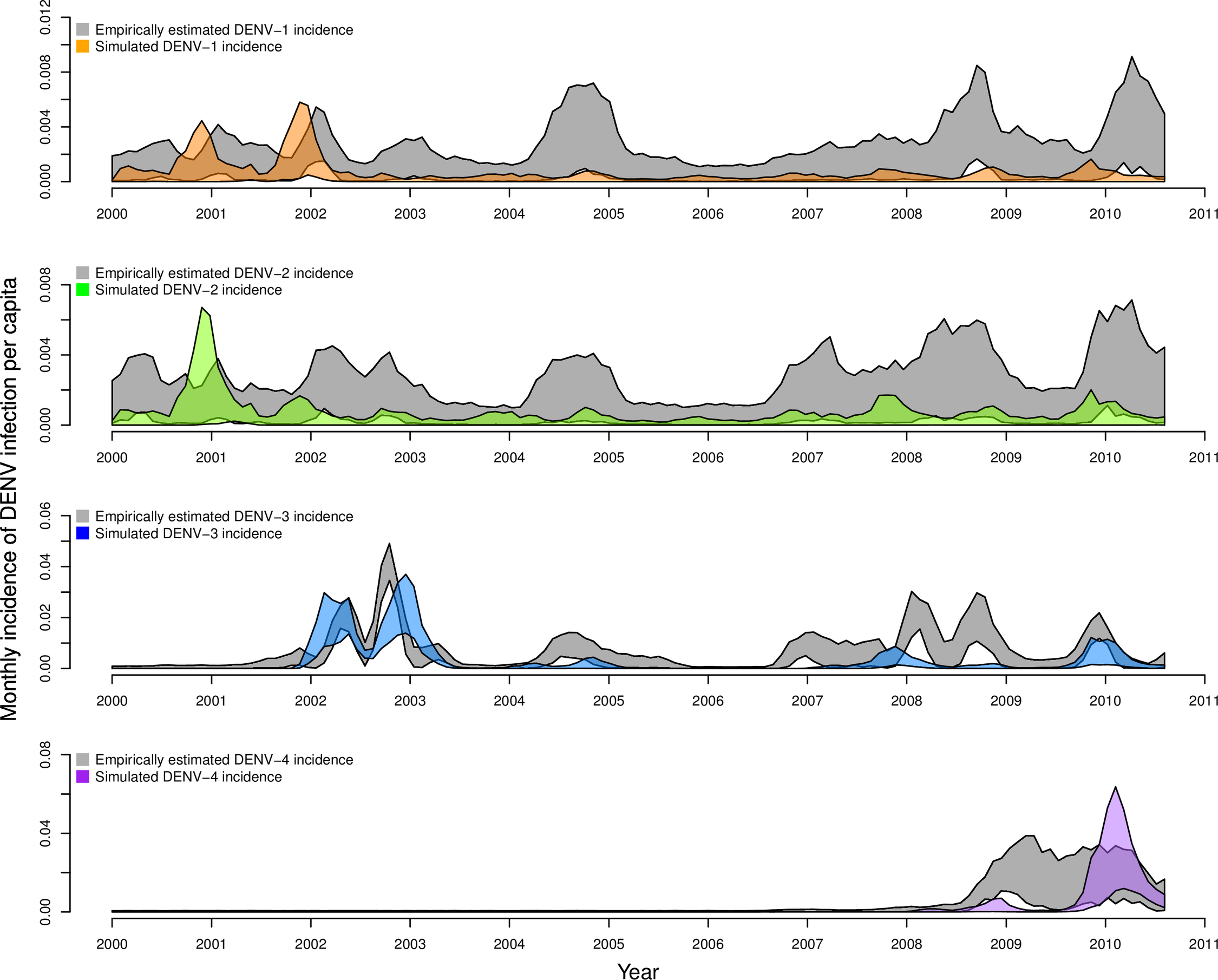

Supplement: S13 Fig — Bands show the range of values in which 95% of simulated values lie for a given serotype in a given month. These values were obtained under the assumption that the duration of temporary cross-immunity was identical for all individuals. Other assumptions followed the default set of assumptions. (TIF) [file pcbi.1006710.s016.tif]

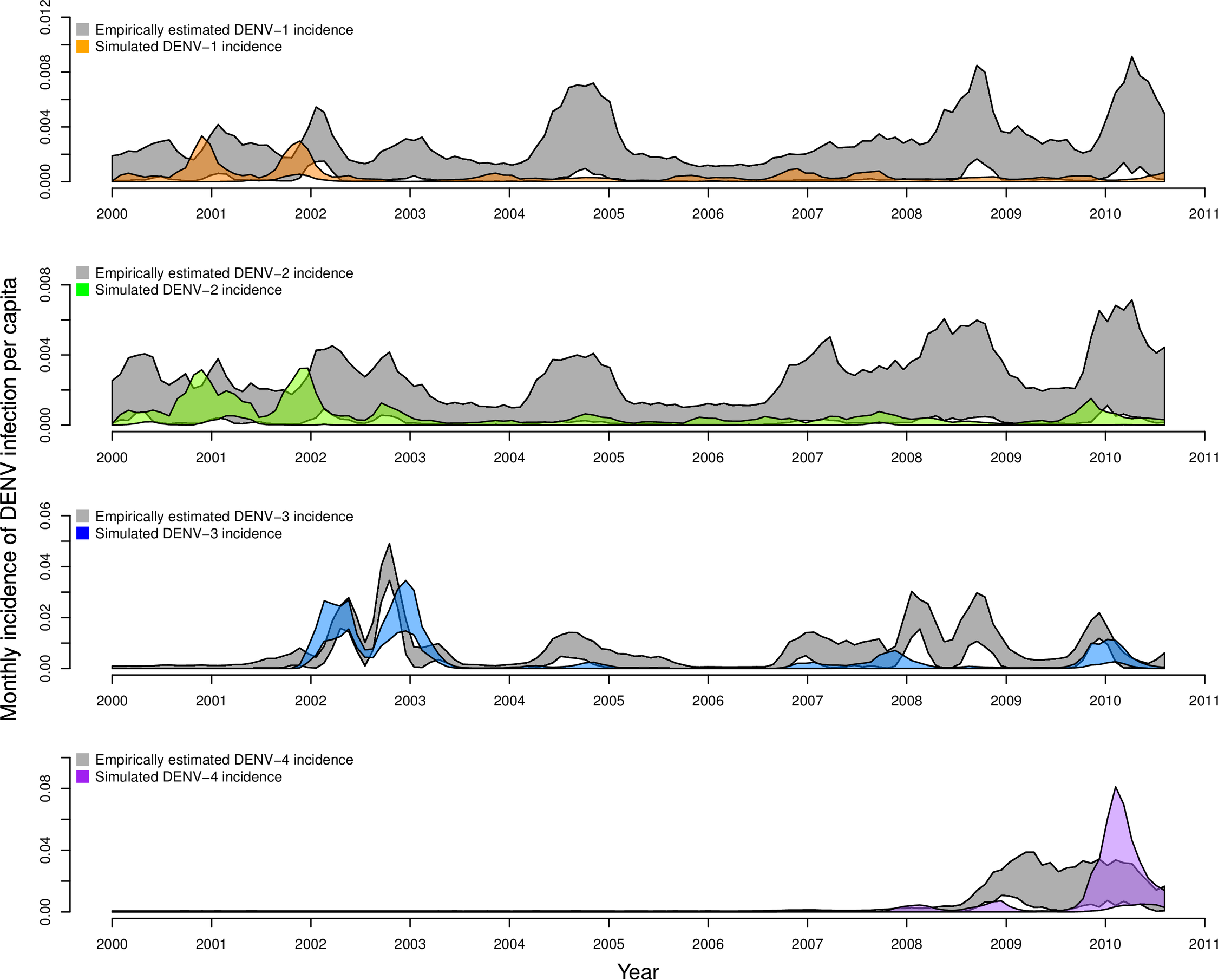

Supplement: S14 Fig — Bands show the range of values in which 95% of simulated values lie for a given serotype in a given month. These values were obtained under the assumption that mosquito infectiousness was 0.5. Other assumptions followed the default set of assumptions. (TIF) [file pcbi.1006710.s017.tif]

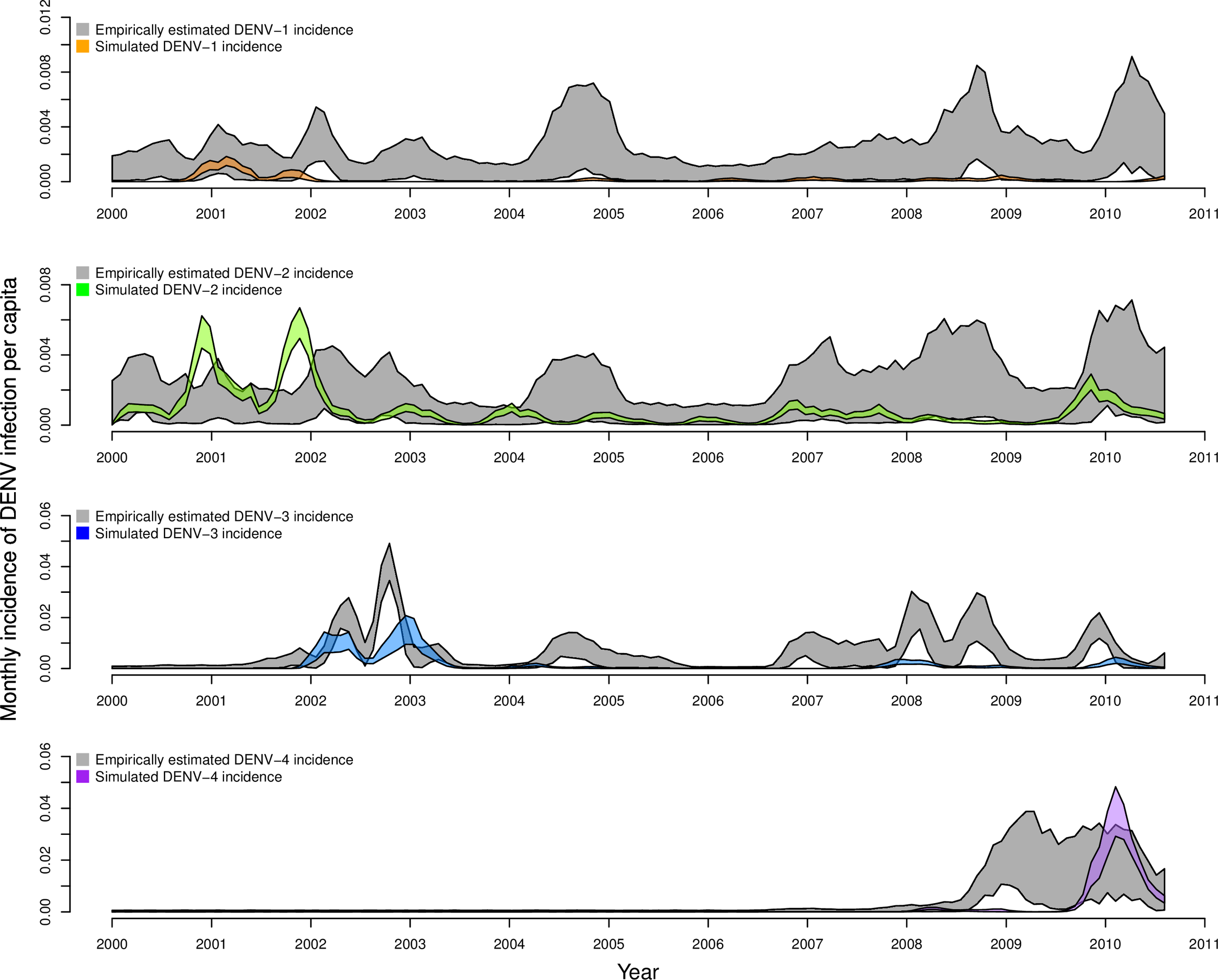

Supplement: S15 Fig — Bands show the range of values in which 95% of simulated values lie for a given serotype in a given month. These values were obtained under the assumption that mosquito biting rate was half that under default assumptions. Other assumptions followed the default set of assumptions. (TIF) [file pcbi.1006710.s018.tif]

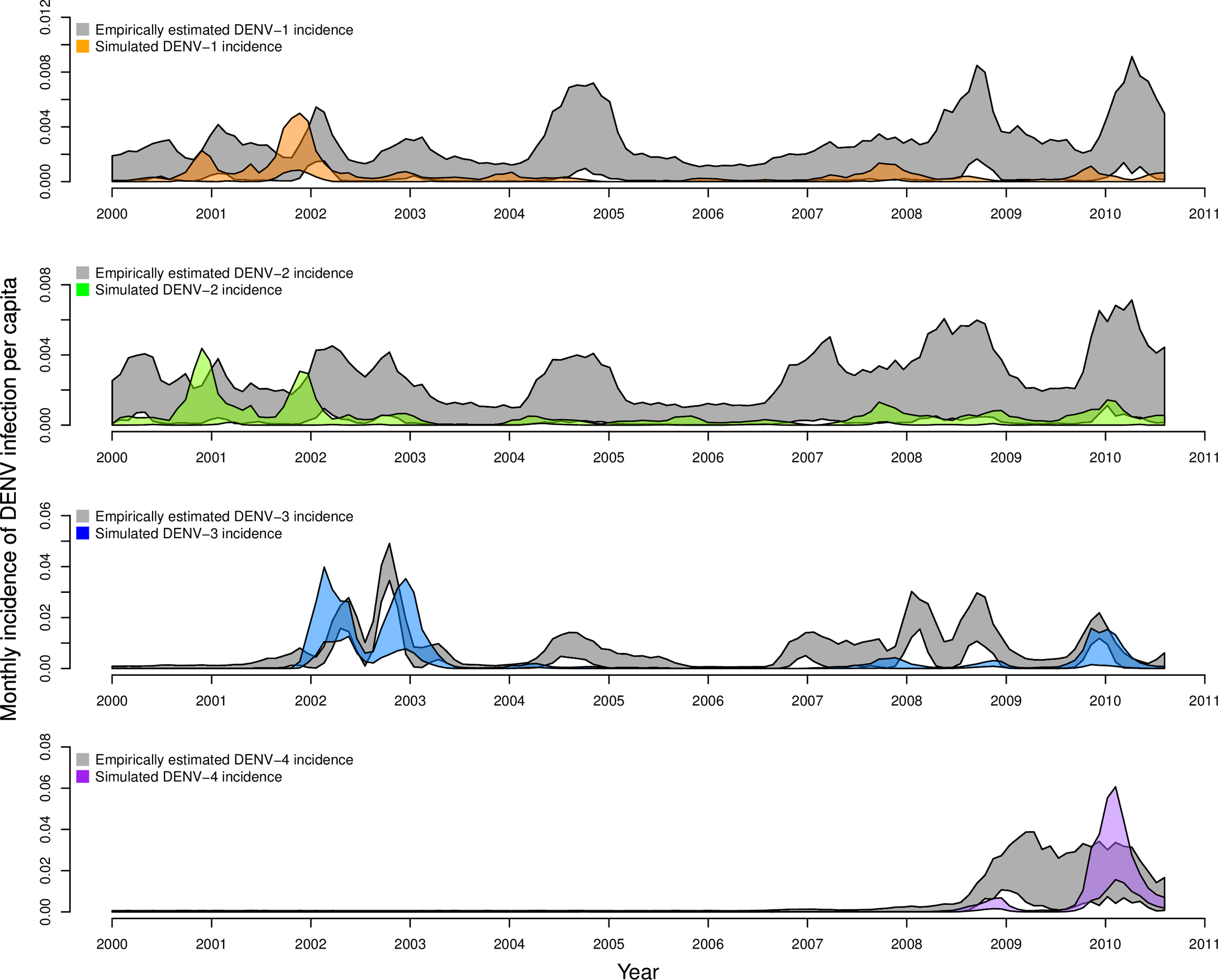

Supplement: S16 Fig — Bands show the range of values in which 95% of simulated values lie for a given serotype in a given month. These values were obtained under the assumption that mosquito biting rate was double that under default assumptions. Other assumptions followed the default set of assumptions. (TIF) [file pcbi.1006710.s019.tif]

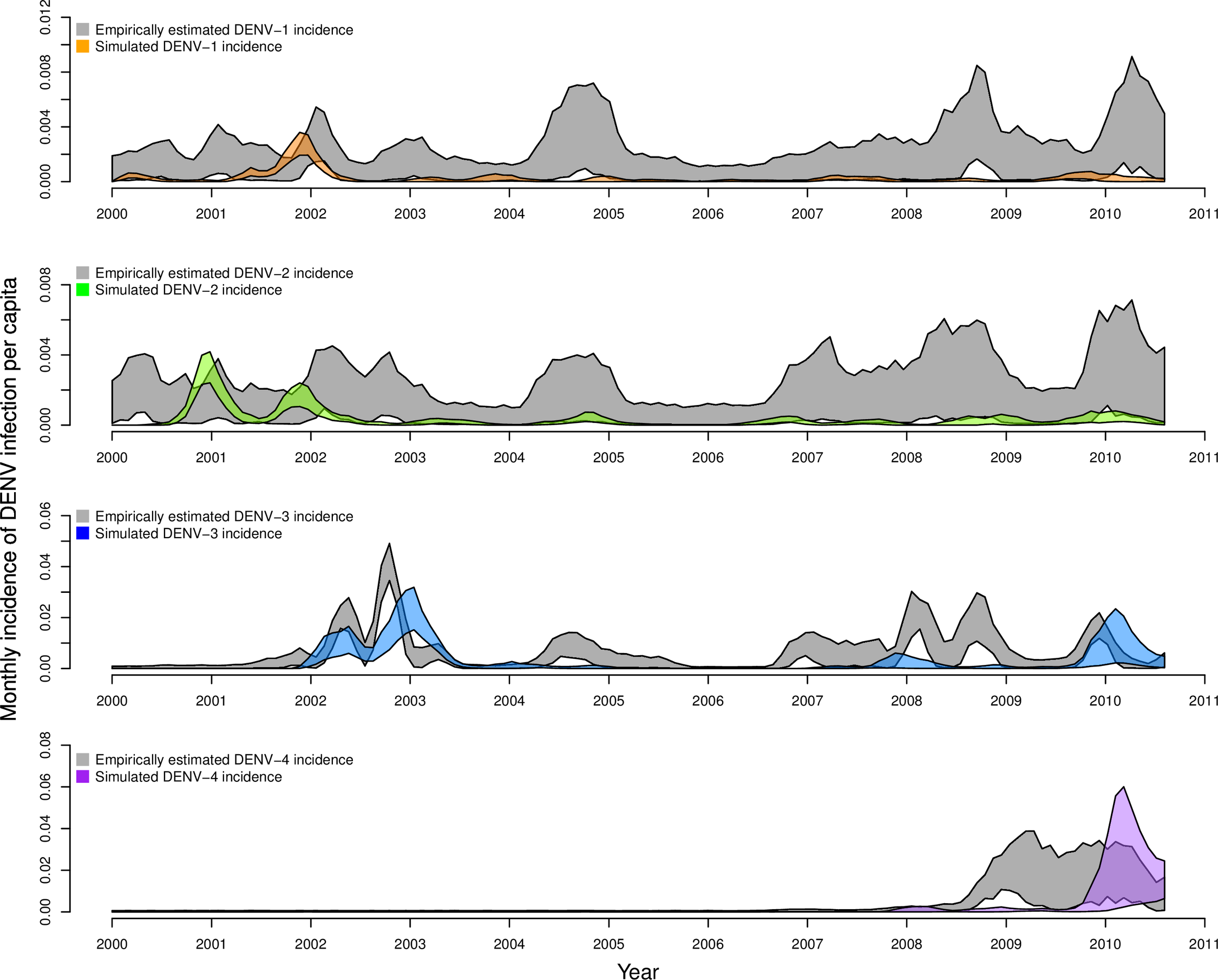

Supplement: S17 Fig — Bands show the range of values in which 95% of simulated values lie for a given serotype in a given month. These values were obtained under the assumption that mosquito death rate was half that under default assumptions. Other assumptions followed the default set of assumptions. (TIF) [file pcbi.1006710.s020.tif]

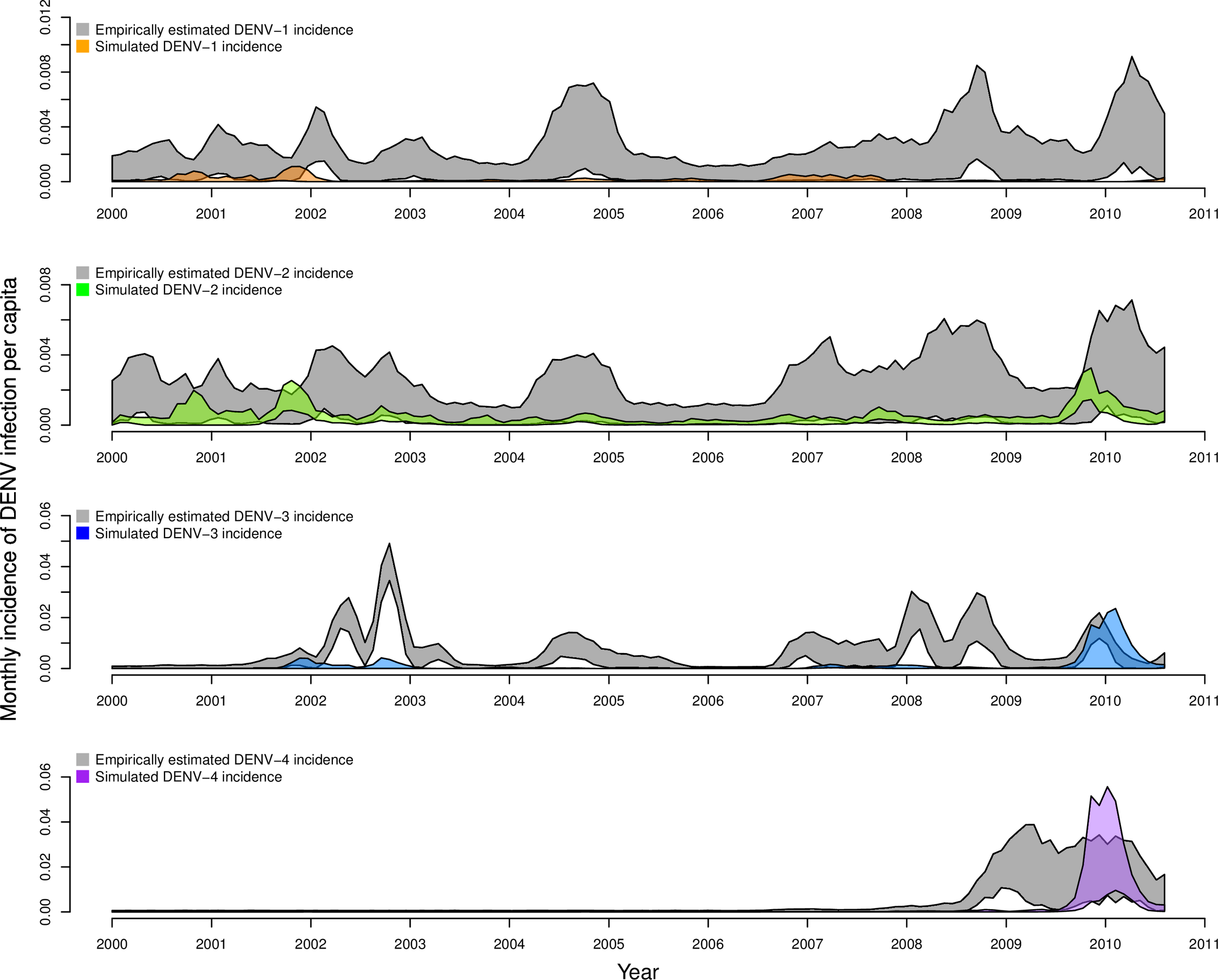

Supplement: S18 Fig — Bands show the range of values in which 95% of simulated values lie for a given serotype in a given month. These values were obtained under the assumption that mosquito death rate was double that under default assumptions. Other assumptions followed the default set of assumptions. (TIF) [file pcbi.1006710.s021.tif]

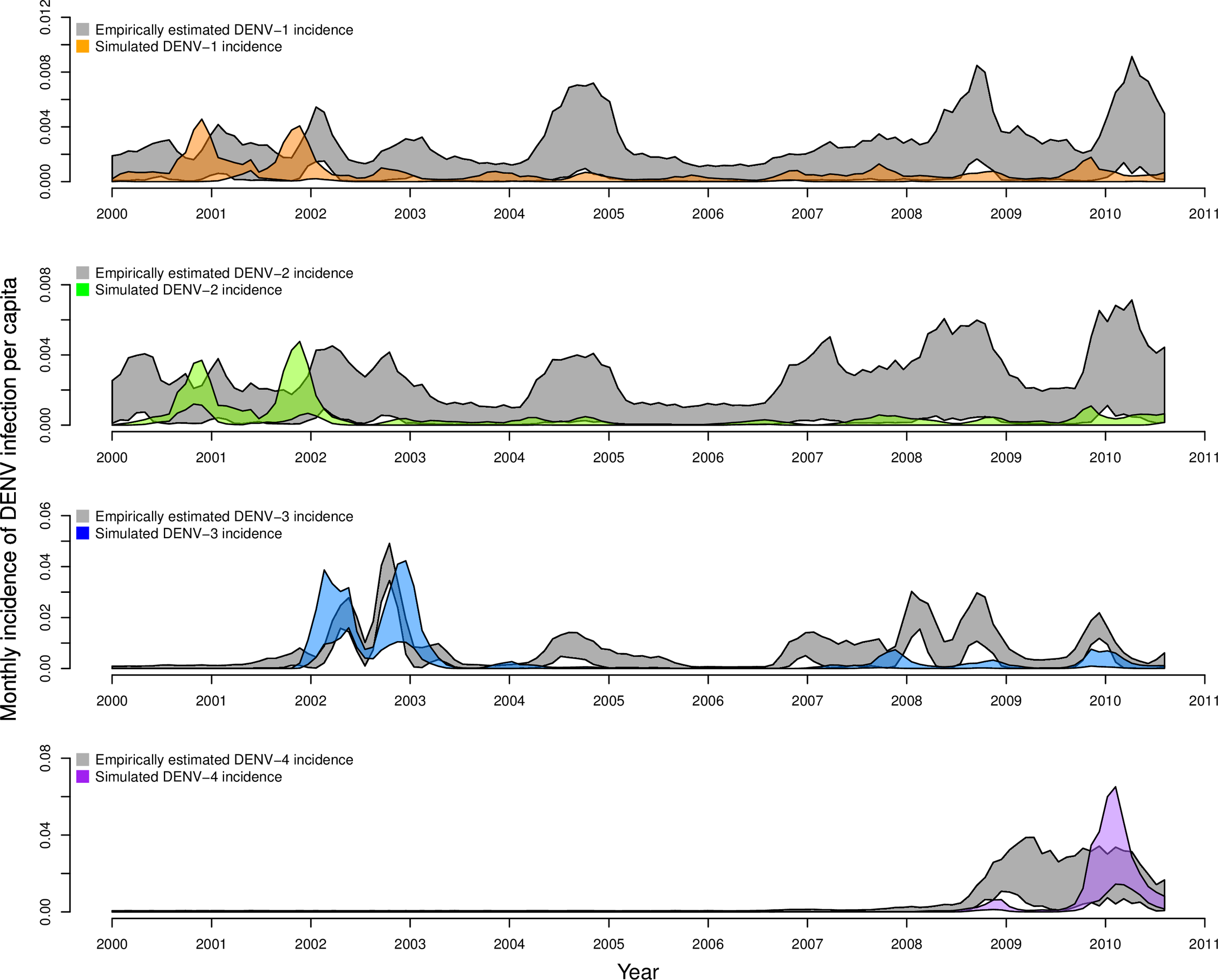

Supplement: S19 Fig — Bands show the range of values in which 95% of simulated values lie for a given serotype in a given month. These values were obtained under the assumption that the extrinsic incubation period was half that under default assumptions. Other assumptions followed the default set of assumptions. (TIF) [file pcbi.1006710.s022.tif]

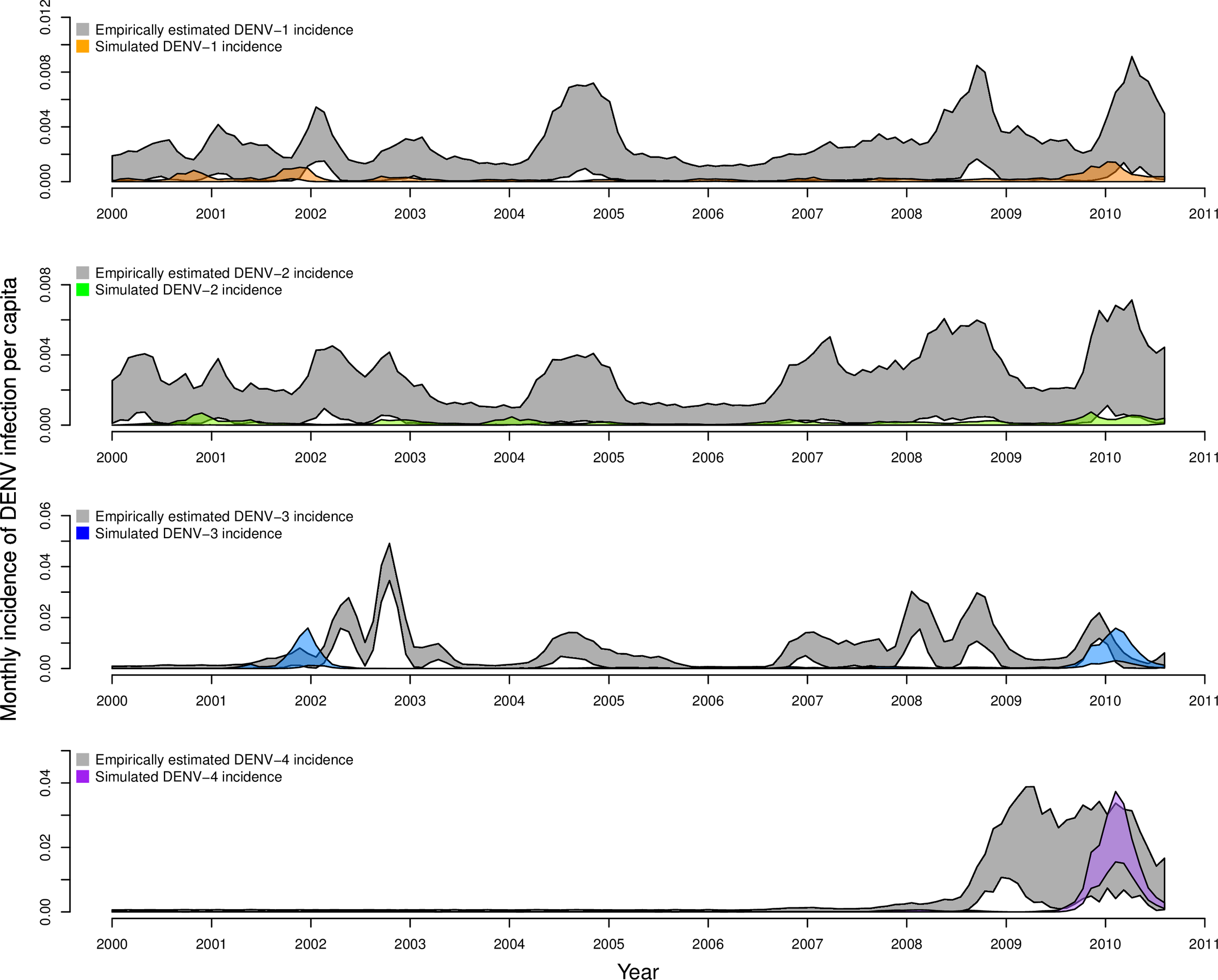

Supplement: S20 Fig — Bands show the range of values in which 95% of simulated values lie for a given serotype in a given month. These values were obtained under the assumption that the extrinsic incubation period was double that under default assumptions. Other assumptions followed the default set of assumptions. (TIF) [file pcbi.1006710.s023.tif]

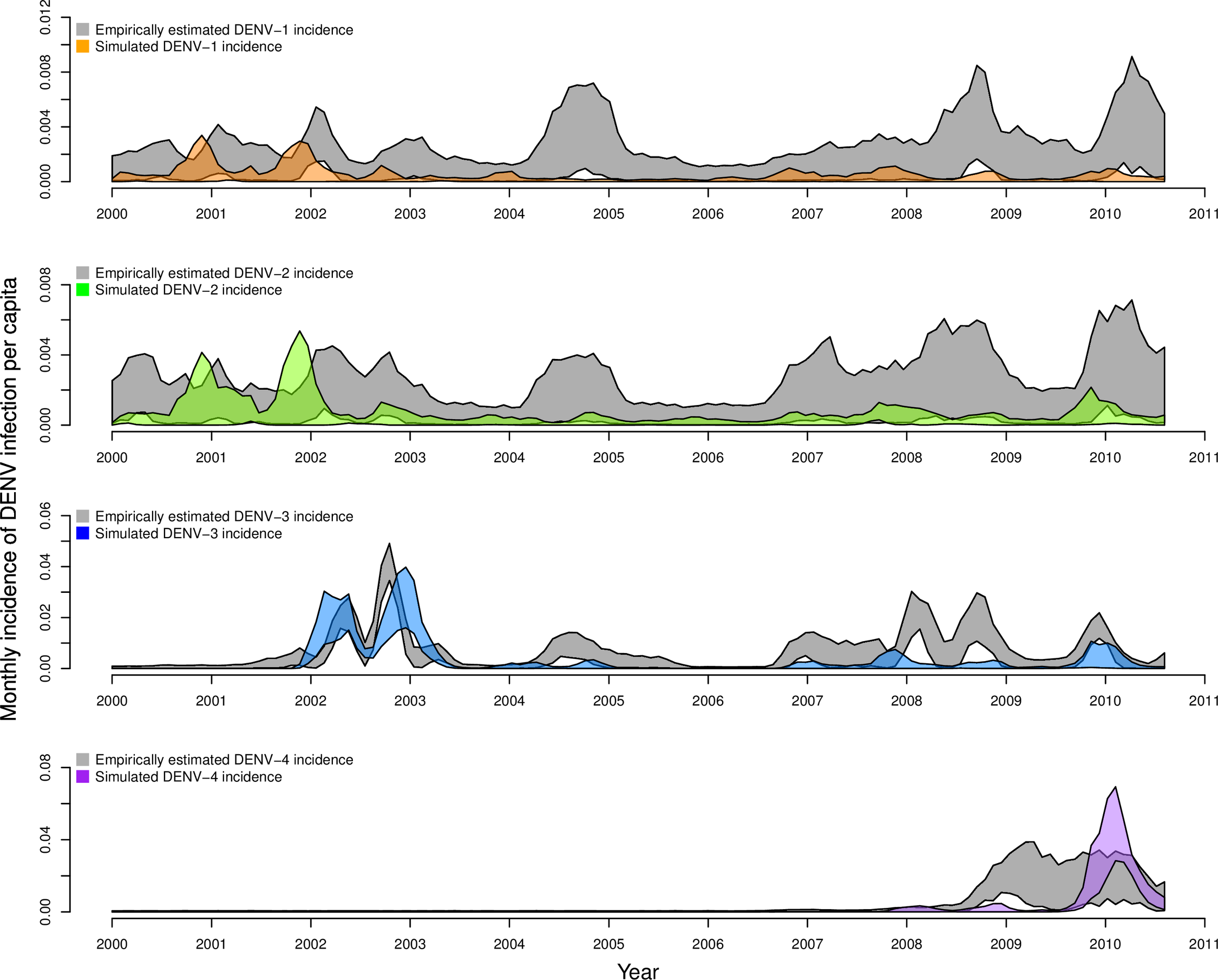

Supplement: S21 Fig — Bands show the range of values in which 95% of simulated values lie for a given serotype in a given month. These values were obtained under the assumption that mosquito movement probability was 0.1. Other assumptions followed the default set of assumptions. (TIF) [file pcbi.1006710.s024.tif]

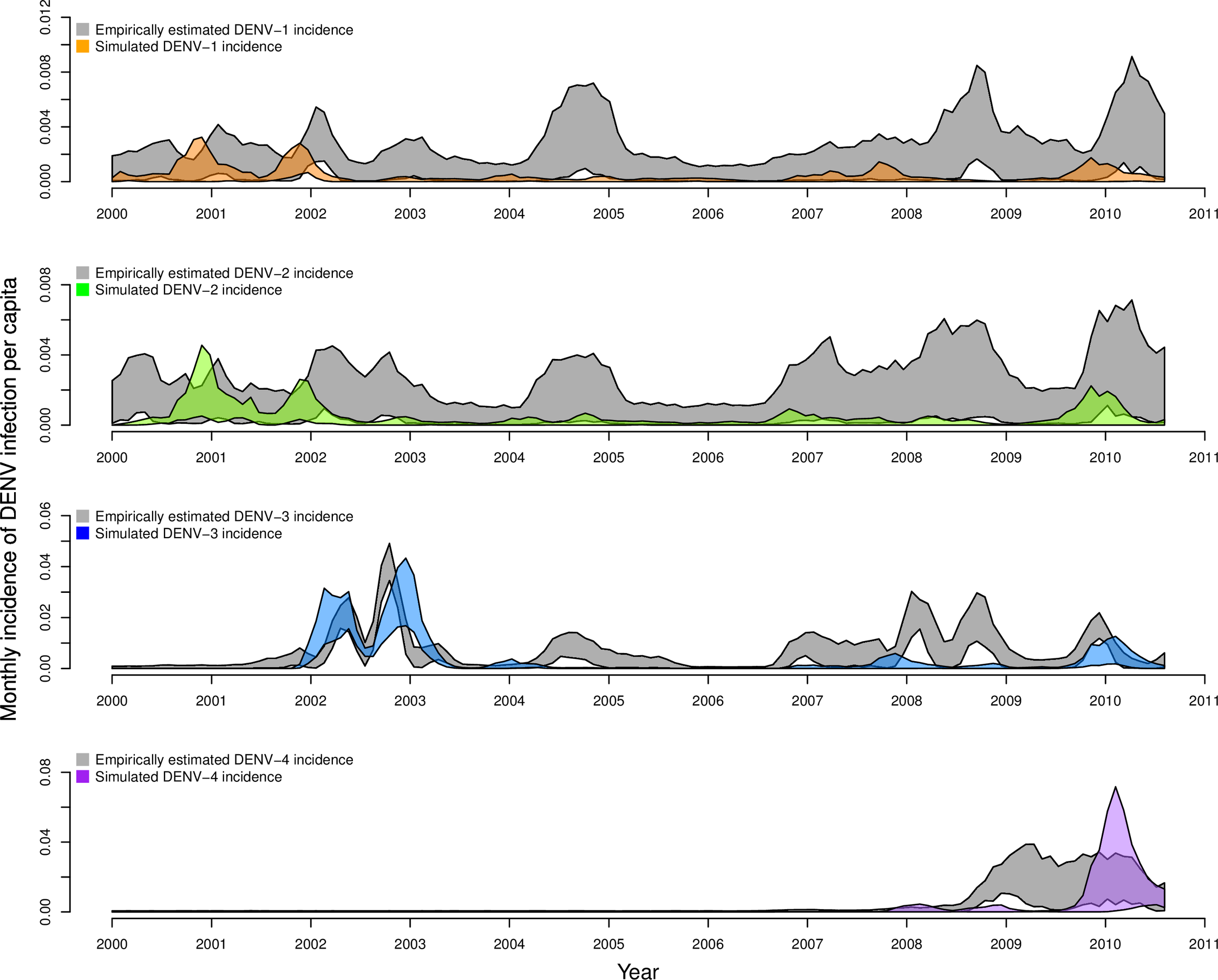

Supplement: S22 Fig — Bands show the range of values in which 95% of simulated values lie for a given serotype in a given month. These values were obtained under the assumption that mosquito movement probability was 0.5. Other assumptions followed the default set of assumptions. (TIF) [file pcbi.1006710.s025.tif]

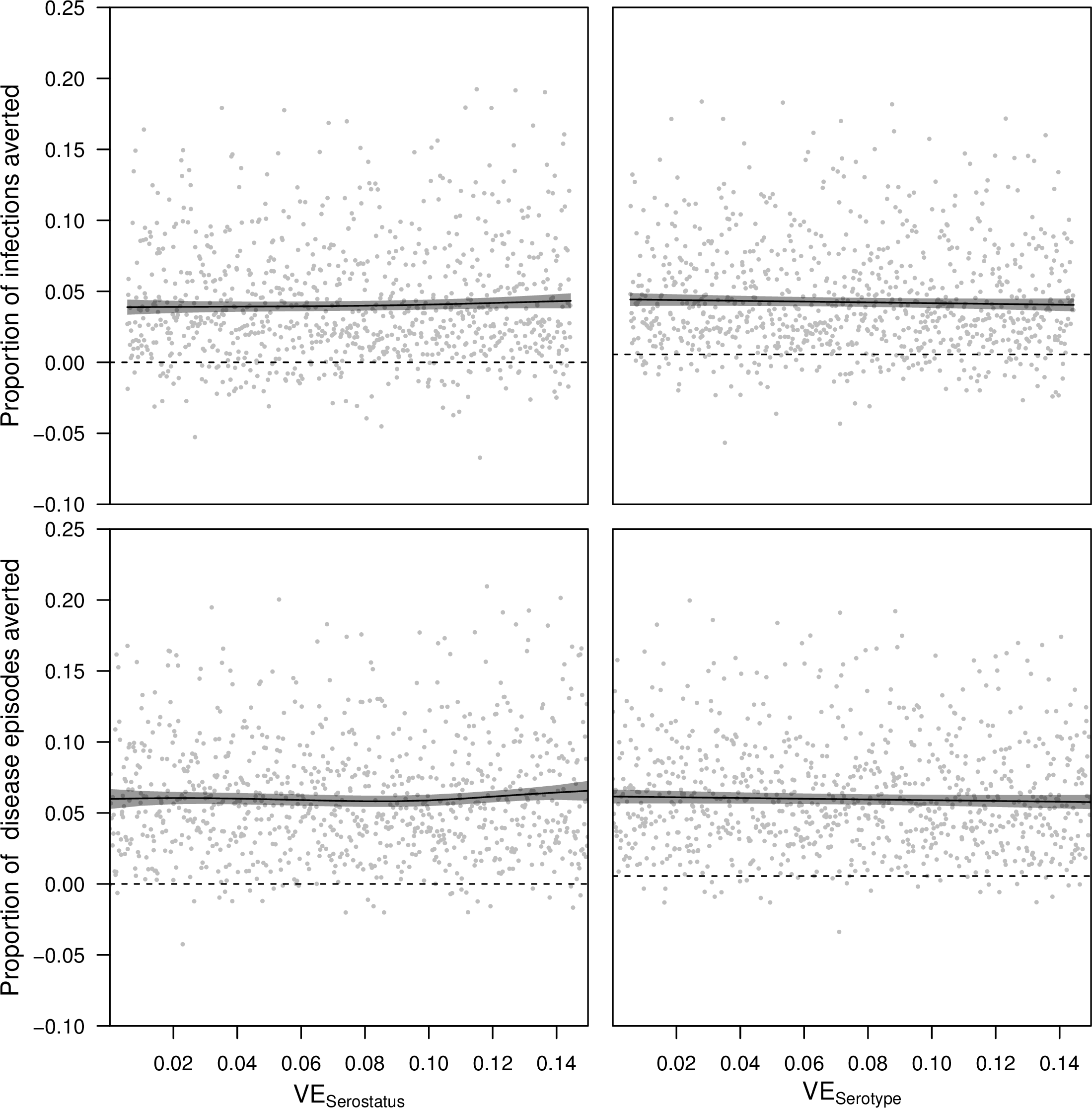

Supplement: S23 Fig — Simulation pairs varied with respect to variation in vaccine efficacy associated with serostatus, VEserostatus, (second column) and variation in vaccine efficacy associated with serotype, VEserotype (right column). The proportion of cumulative infections averted (top row) and cumulative disease episodes averted (bottom row) were based on the number of each in the simulation without vaccination minus the number of each in the simulation with vaccination, both following 20 years of routine vaccination of 9-year olds at 80% coverage. Lines show the proportion of infections or disease episodes averted as a function of each parameter varied on the x-axis, as estimated by a generalized additive model with independent smooth terms for each parameter. When one parameter is varied, the other is held constant at the midpoint of its range, as are p and VEmean. Gray bands indicate 95% confidence intervals. (TIF) [file pcbi.1006710.s026.tif]

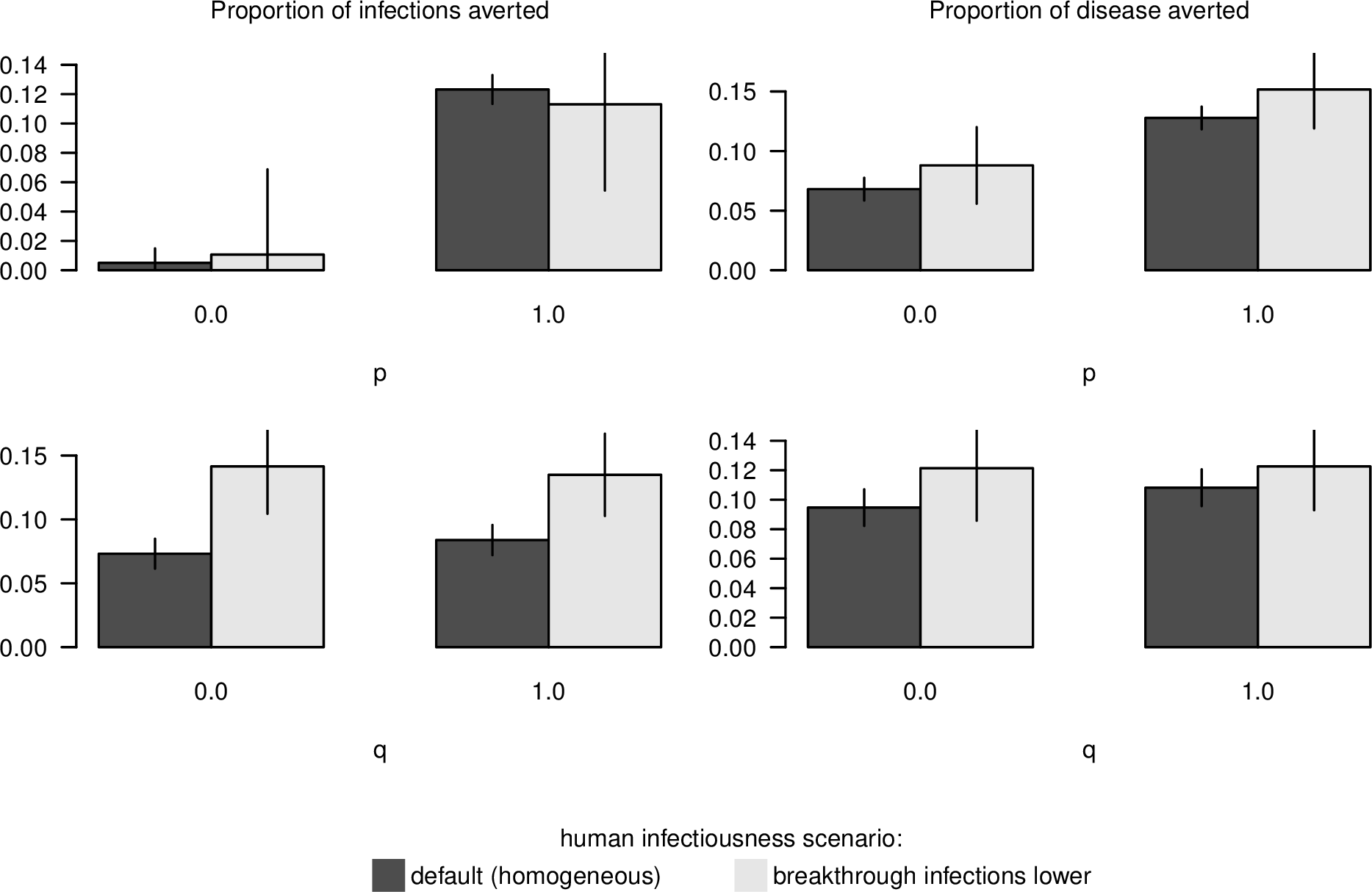

Supplement: S24 Fig — Bars display point estimates and 95% confidence intervals obtained from fitting generalized additive models to simulation results across the range of each parameter while holding others at the midpoints of their ranges. These values are comparable to the extremes displayed in Fig 8 (p, q) but under different assumptions about human infectiousness. (TIF) [file pcbi.1006710.s027.tif]

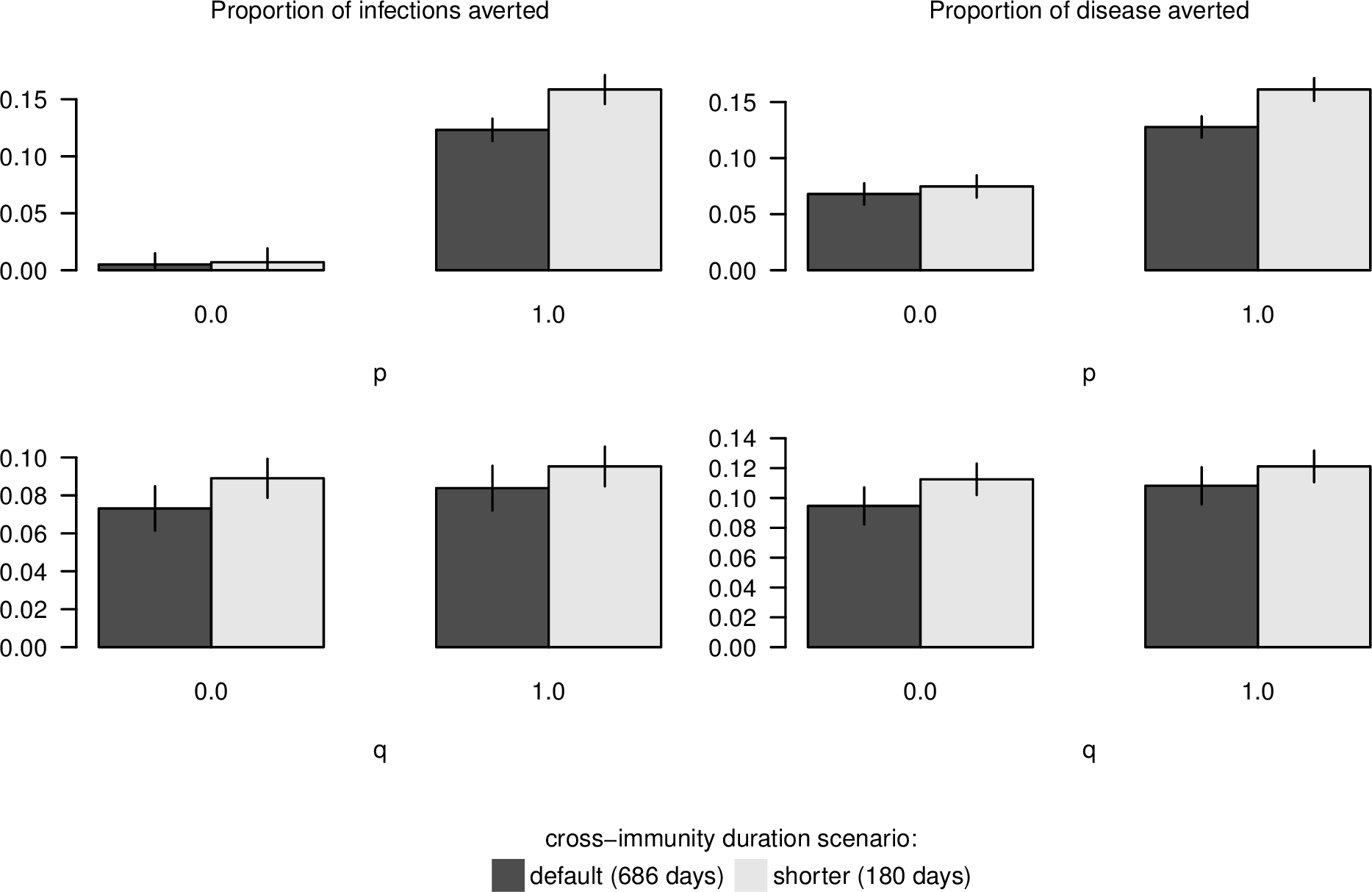

Supplement: S25 Fig — Bars display point estimates and 95% confidence intervals obtained from fitting generalized additive models to simulation results across the range of each parameter while holding others at the midpoints of their ranges. These values are comparable to the extremes displayed in Fig 8 (p, q) but under different assumptions about the duration of cross-immunity. (TIF) [file pcbi.1006710.s028.tif]

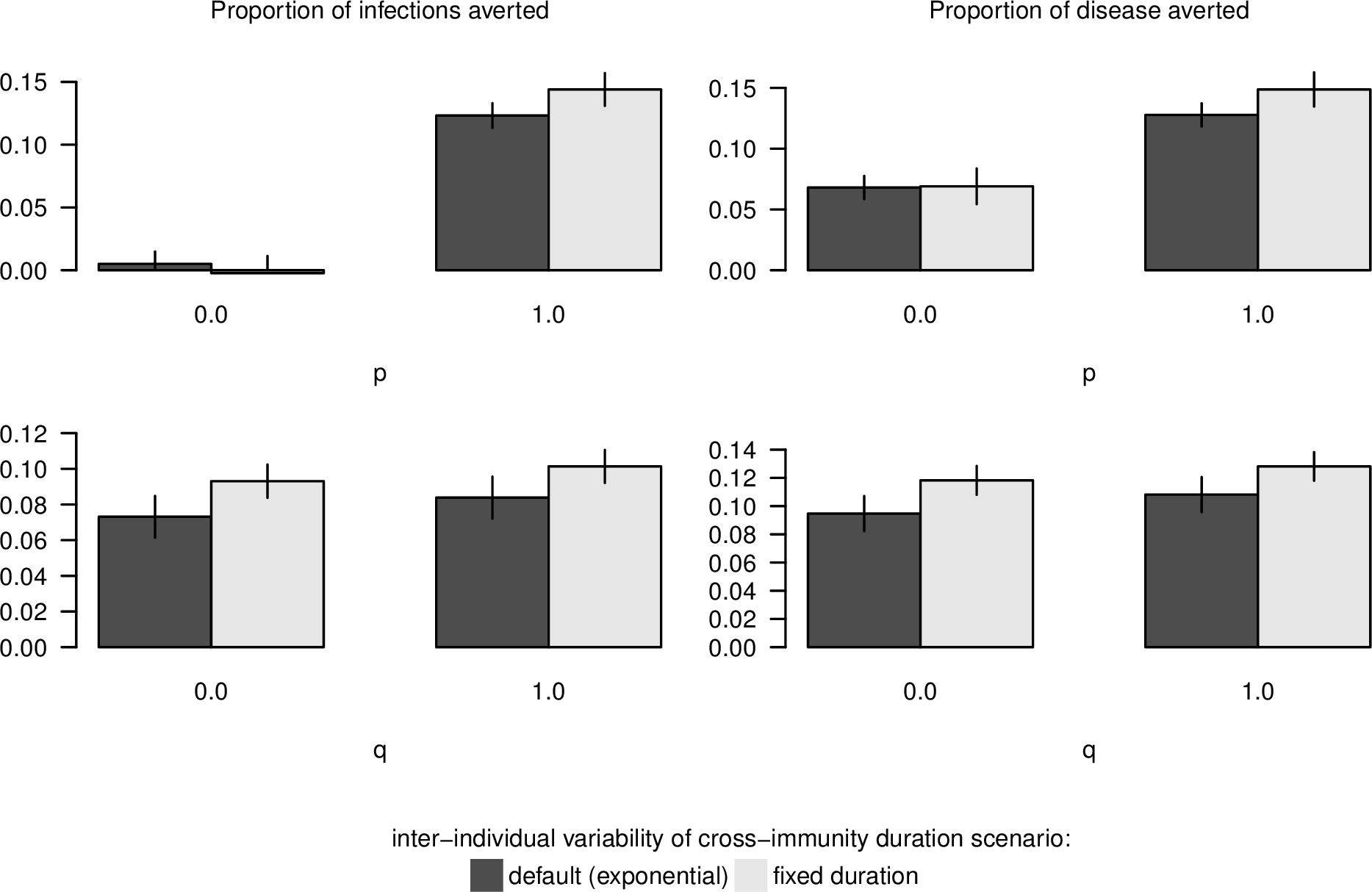

Supplement: S26 Fig — Bars display point estimates and 95% confidence intervals obtained from fitting generalized additive models to simulation results across the range of each parameter while holding others at the midpoints of their ranges. These values are comparable to the extremes displayed in Fig 8 (p, q) but under different assumptions about inter-individual variability in the duration of cross-immunity. (TIF) [file pcbi.1006710.s029.tif]

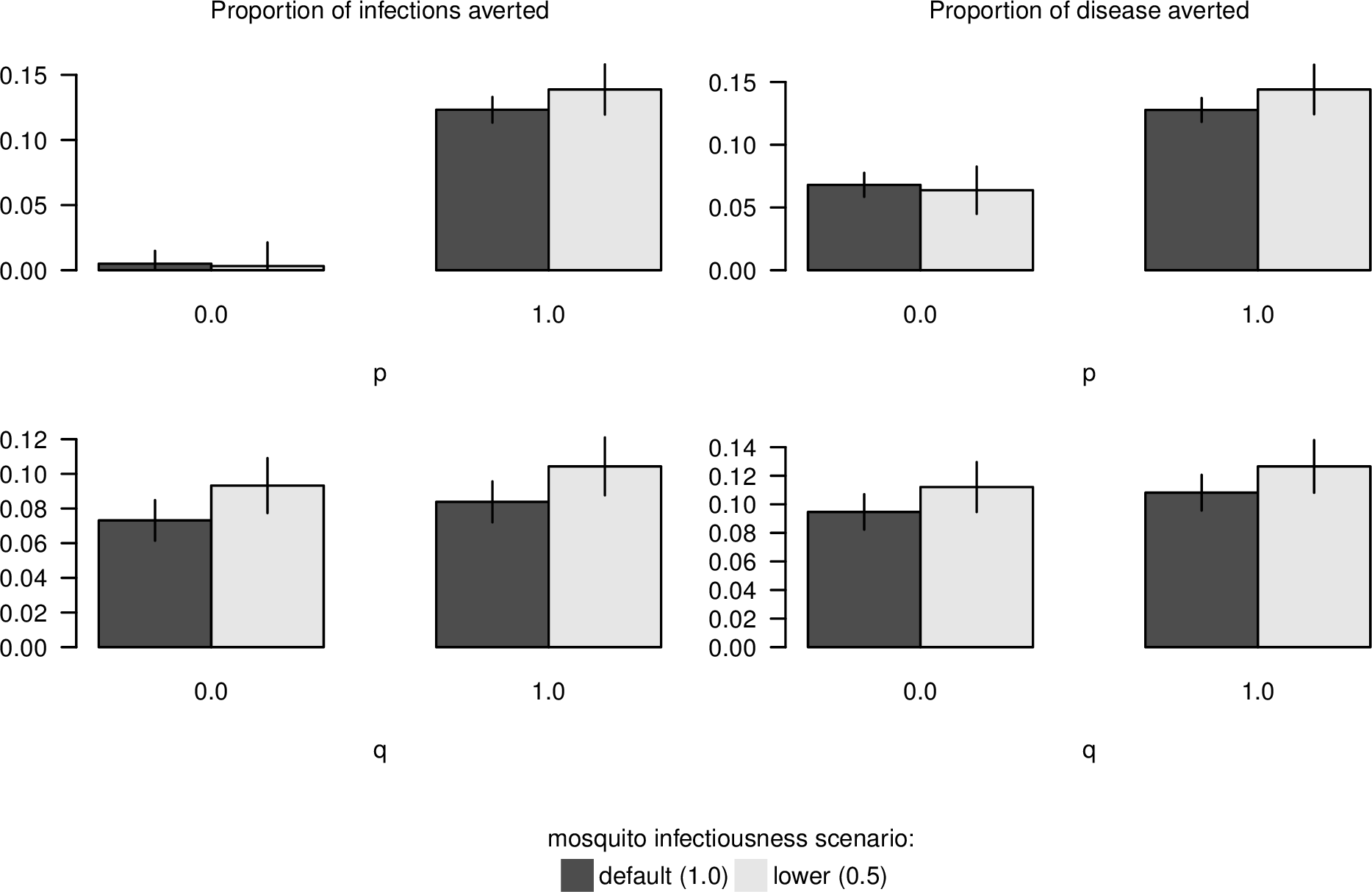

Supplement: S27 Fig — Bars display point estimates and 95% confidence intervals obtained from fitting generalized additive models to simulation results across the range of each parameter while holding others at the midpoints of their ranges. These values are comparable to the extremes displayed in Fig 8 (p, q) but under different assumptions about mosquito infectiousness. (TIF) [file pcbi.1006710.s030.tif]

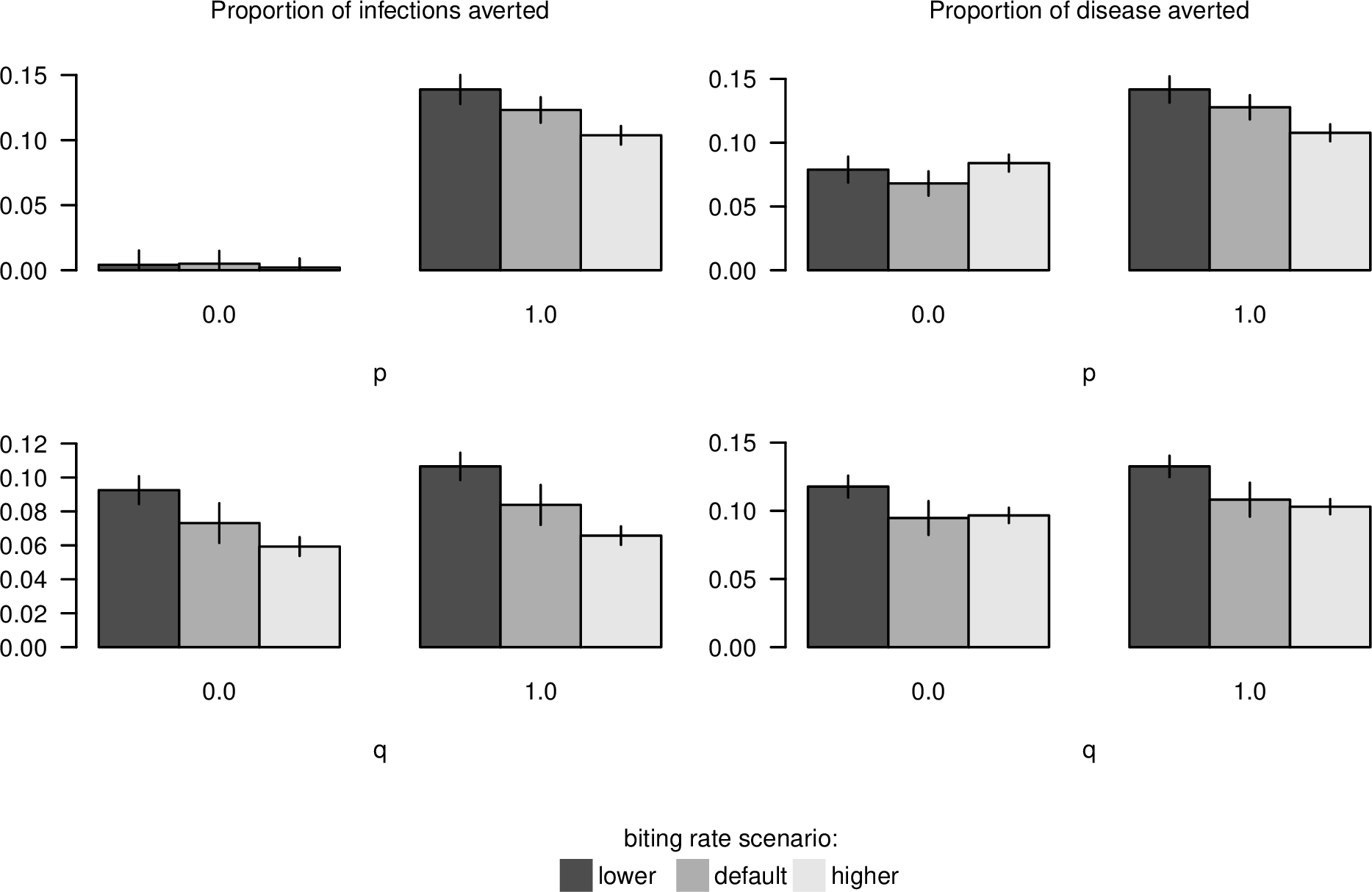

Supplement: S28 Fig — Bars display point estimates and 95% confidence intervals obtained from fitting generalized additive models to simulation results across the range of each parameter while holding others at the midpoints of their ranges. These values are comparable to the extremes displayed in Fig 8 (p, q) but under different assumptions about mosquito biting rate. (TIF) [file pcbi.1006710.s031.tif]

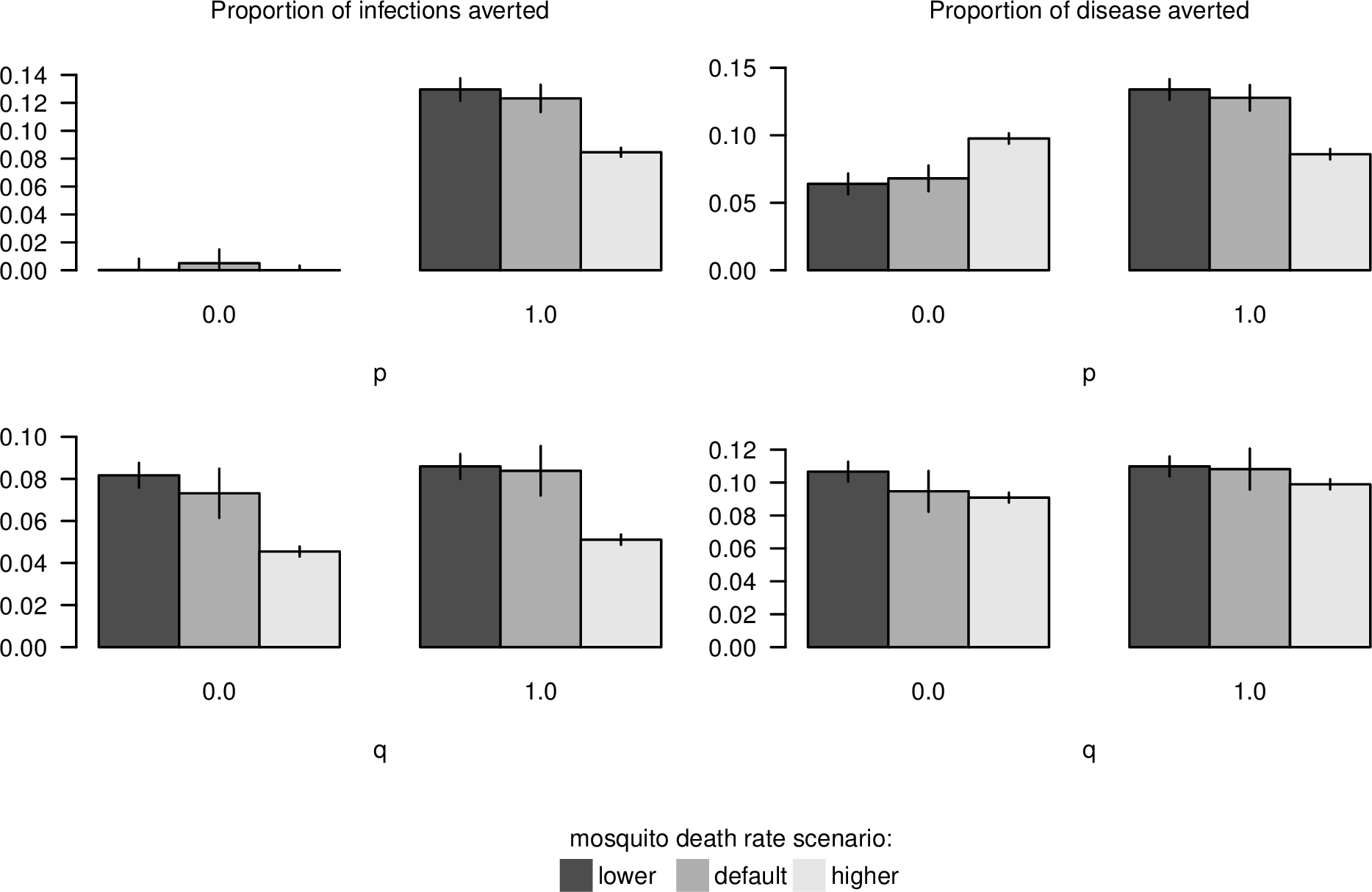

Supplement: S29 Fig — Bars display point estimates and 95% confidence intervals obtained from fitting generalized additive models to simulation results across the range of each parameter while holding others at the midpoints of their ranges. These values are comparable to the extremes displayed in Fig 8 (p, q) but under different assumptions about mosquito death rate. (TIF) [file pcbi.1006710.s032.tif]

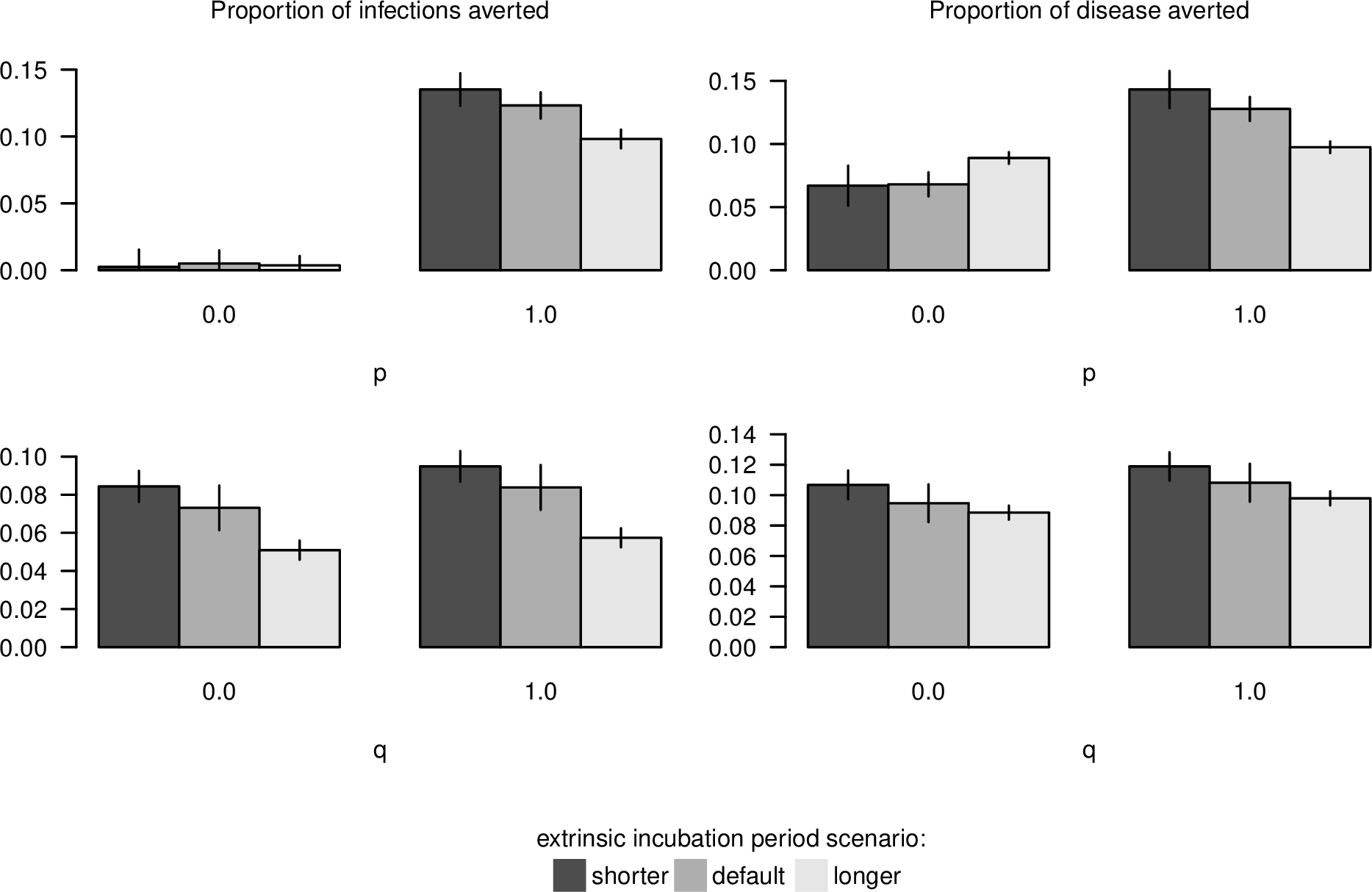

Supplement: S30 Fig — Bars display point estimates and 95% confidence intervals obtained from fitting generalized additive models to simulation results across the range of each parameter while holding others at the midpoints of their ranges. These values are comparable to the extremes displayed in Fig 8 (p, q) but under different assumptions about the extrinsic incubation period. (TIF) [file pcbi.1006710.s033.tif]

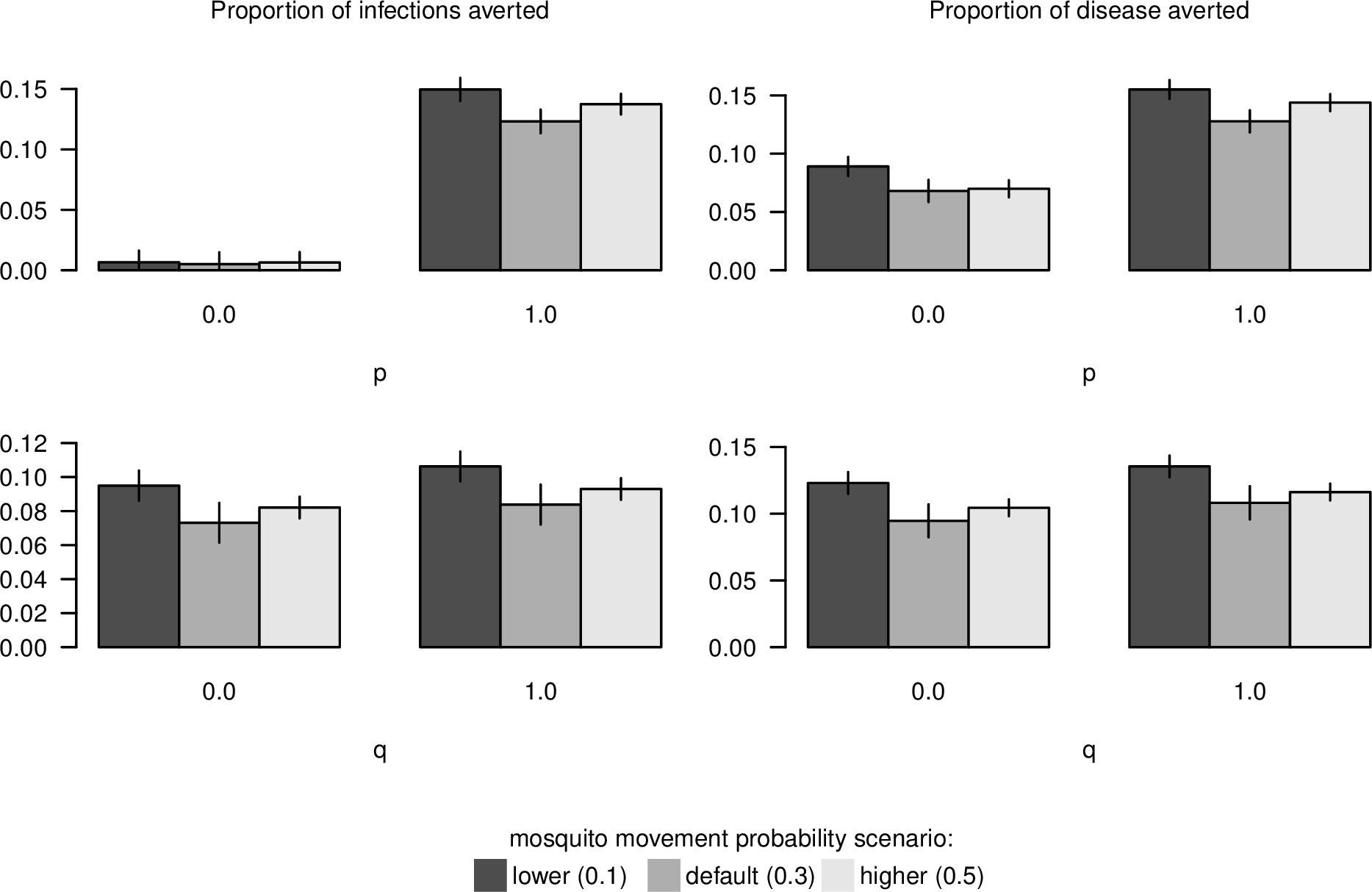

Supplement: S31 Fig — Bars display point estimates and 95% confidence intervals obtained from fitting generalized additive models to simulation results across the range of each parameter while holding others at the midpoints of their ranges. These values are comparable to the extremes displayed in Fig 8 (p, q) but under different assumptions about mosquito movement probability. (TIF) [file pcbi.1006710.s034.tif]

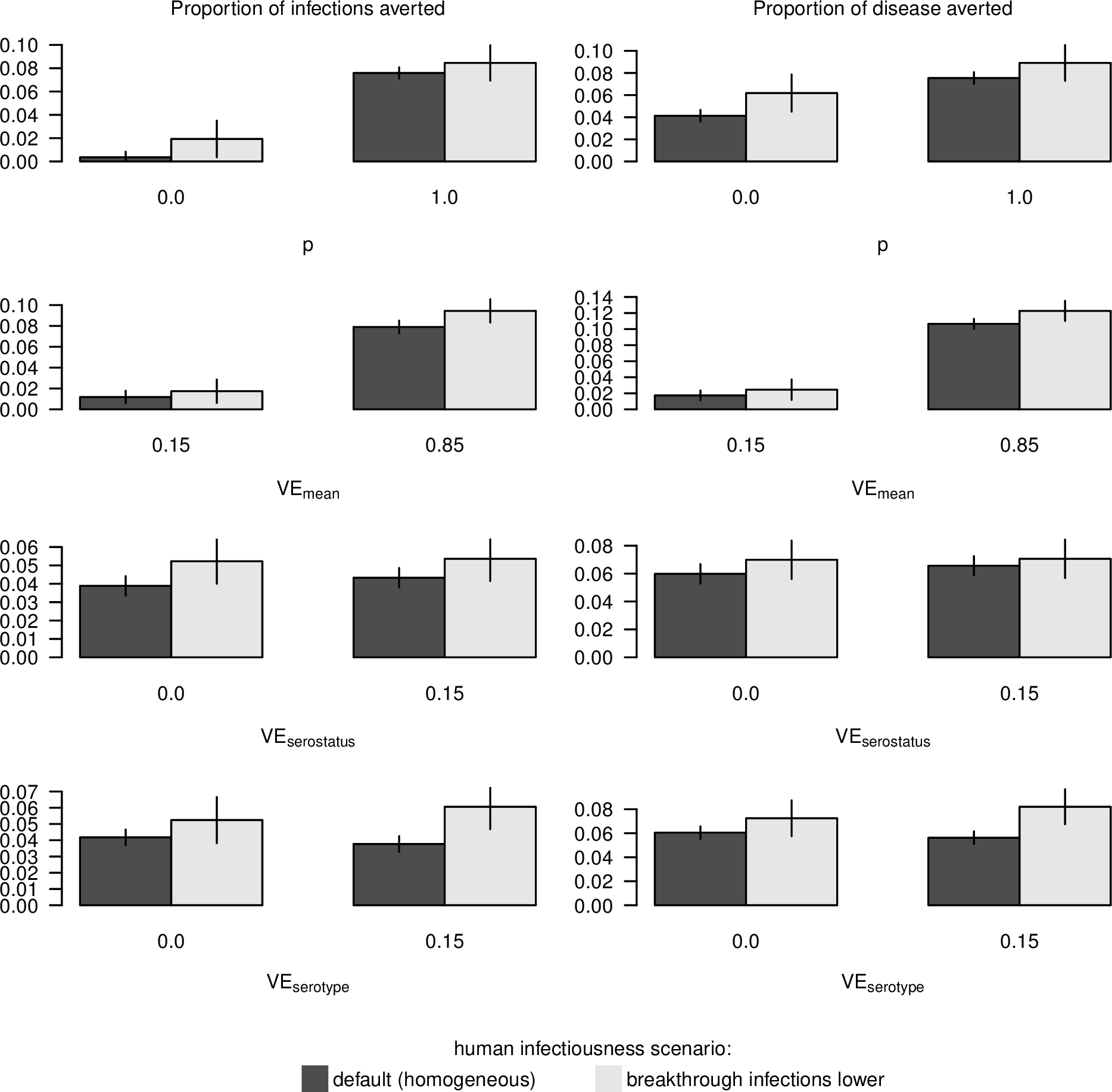

Supplement: S32 Fig — Bars display point estimates and 95% confidence intervals obtained from fitting generalized additive models to simulation results across the range of each parameter while holding others at the midpoints of their ranges. These values are comparable to the extremes displayed in Fig 9 (p, VEmean) and S23 Fig (VEserostatus, VEserotype) but under different assumptions about human infectiousness. (TIF) [file pcbi.1006710.s035.tif]

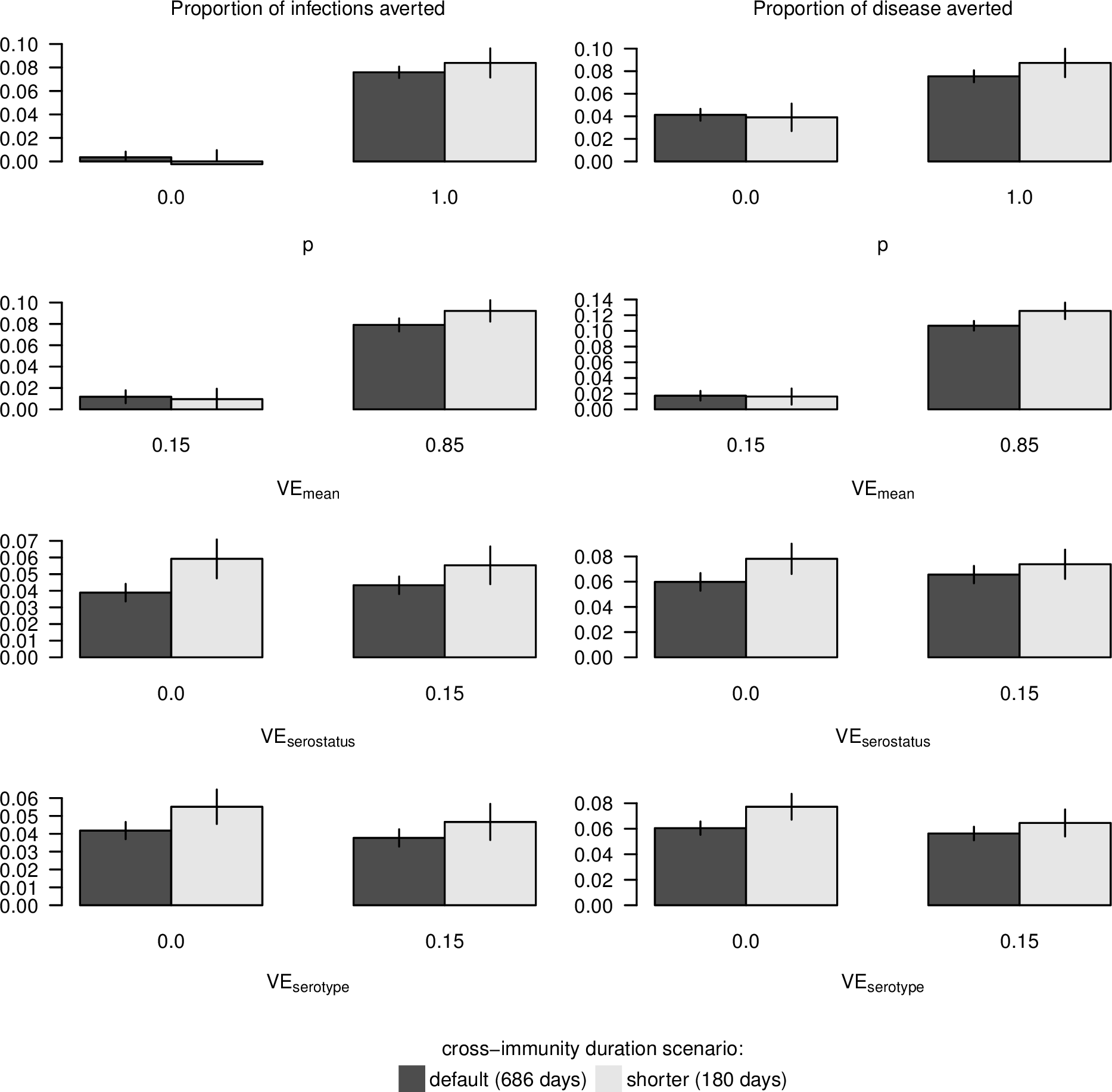

Supplement: S33 Fig — Bars display point estimates and 95% confidence intervals obtained from fitting generalized additive models to simulation results across the range of each parameter while holding others at the midpoints of their ranges. These values are comparable to the extremes displayed in Fig 9 (p, VEmean) and S23 Fig (VEserostatus, VEserotype) but under different assumptions about the duration of cross-immunity. (TIF) [file pcbi.1006710.s036.tif]

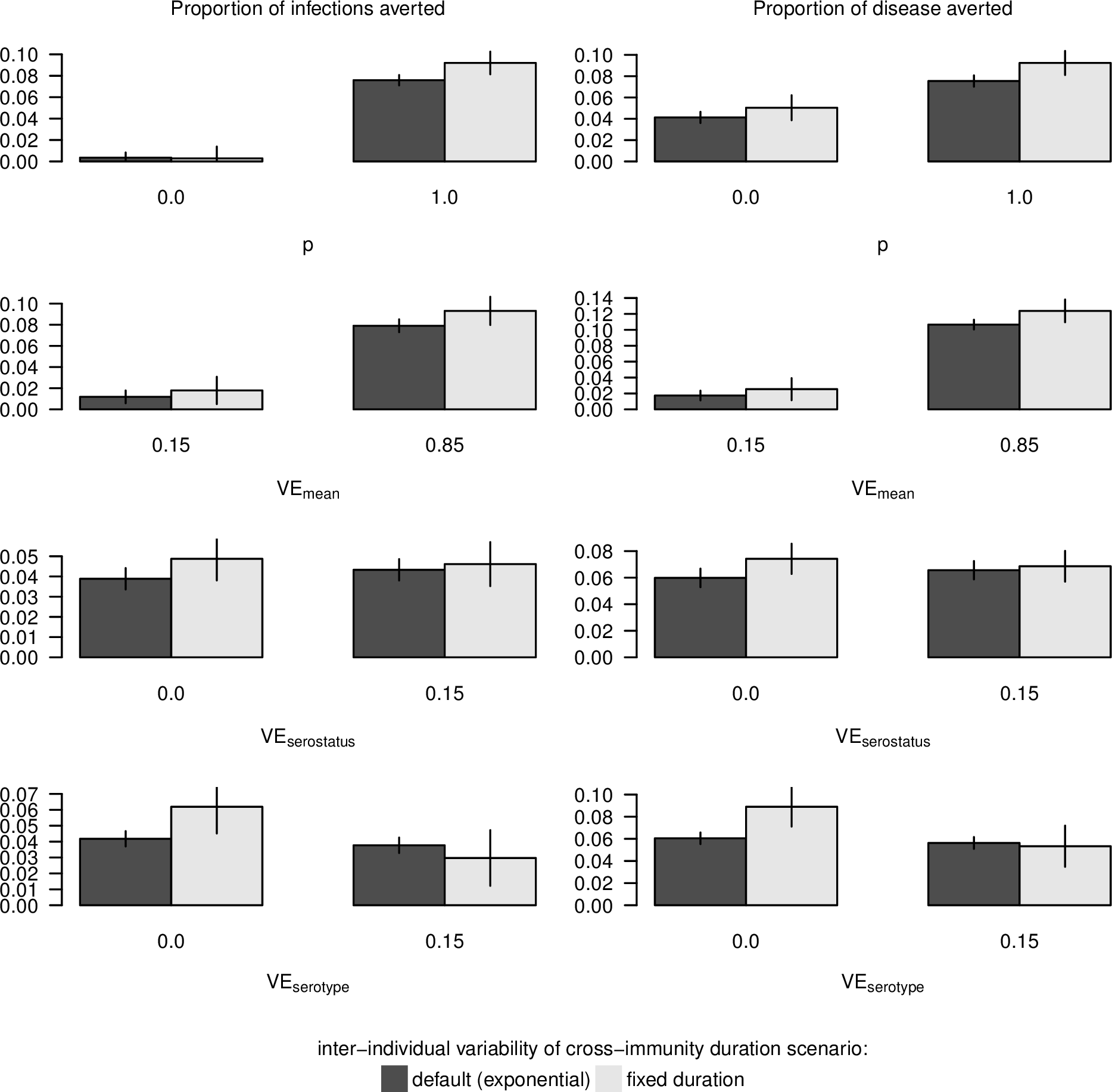

Supplement: S34 Fig — Bars display point estimates and 95% confidence intervals obtained from fitting generalized additive models to simulation results across the range of each parameter while holding others at the midpoints of their ranges. These values are comparable to the extremes displayed in Fig 9 (p, VEmean) and S23 Fig (VEserostatus, VEserotype) but under different assumptions about inter-individual variability in the duration of cross-immunity. (TIF) [file pcbi.1006710.s037.tif]

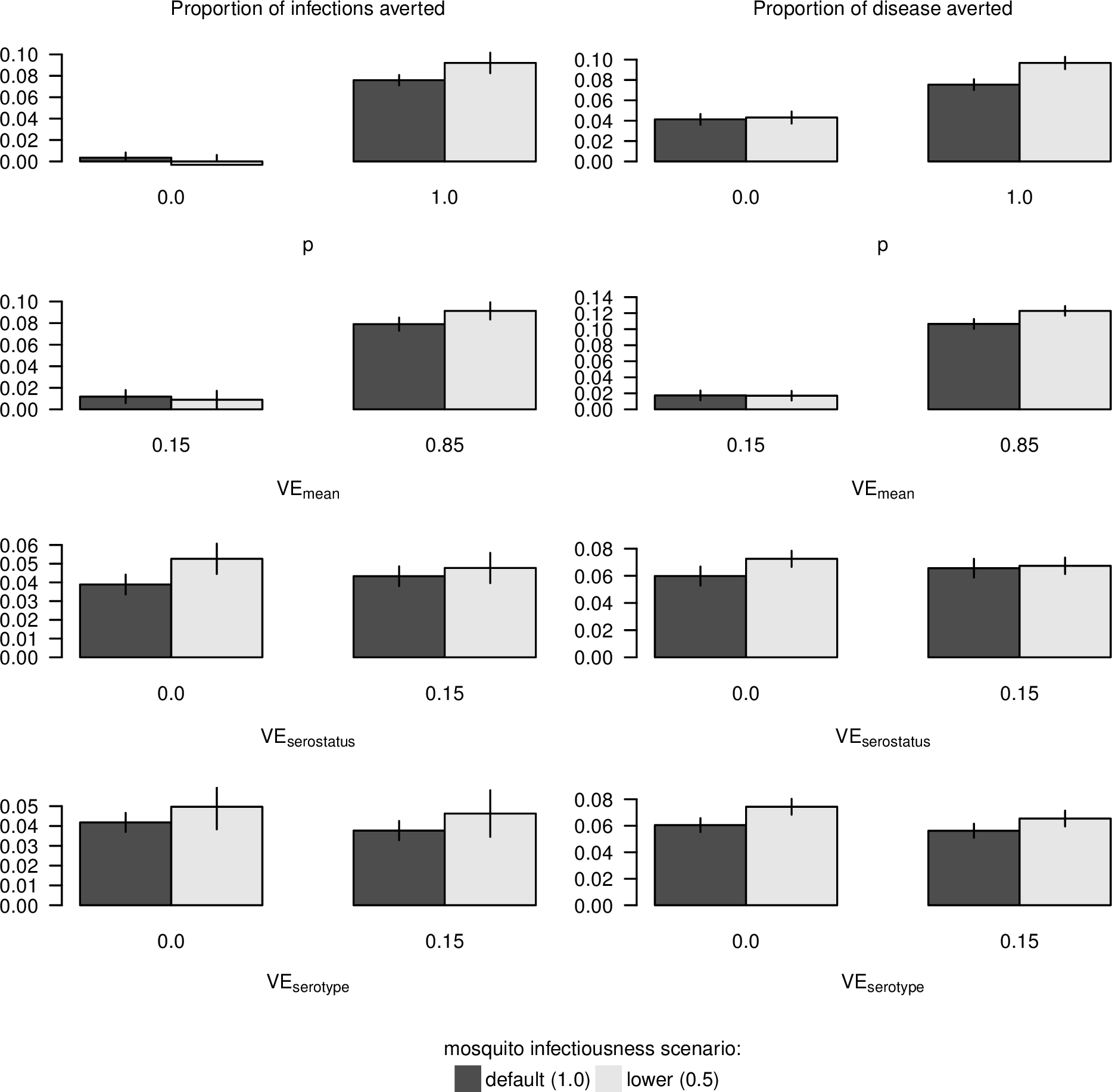

Supplement: S35 Fig — Bars display point estimates and 95% confidence intervals obtained from fitting generalized additive models to simulation results across the range of each parameter while holding others at the midpoints of their ranges. These values are comparable to the extremes displayed in Fig 9 (p, VEmean) and S23 Fig (VEserostatus, VEserotype) but under different assumptions about mosquito infectiousness. (TIF) [file pcbi.1006710.s038.tif]

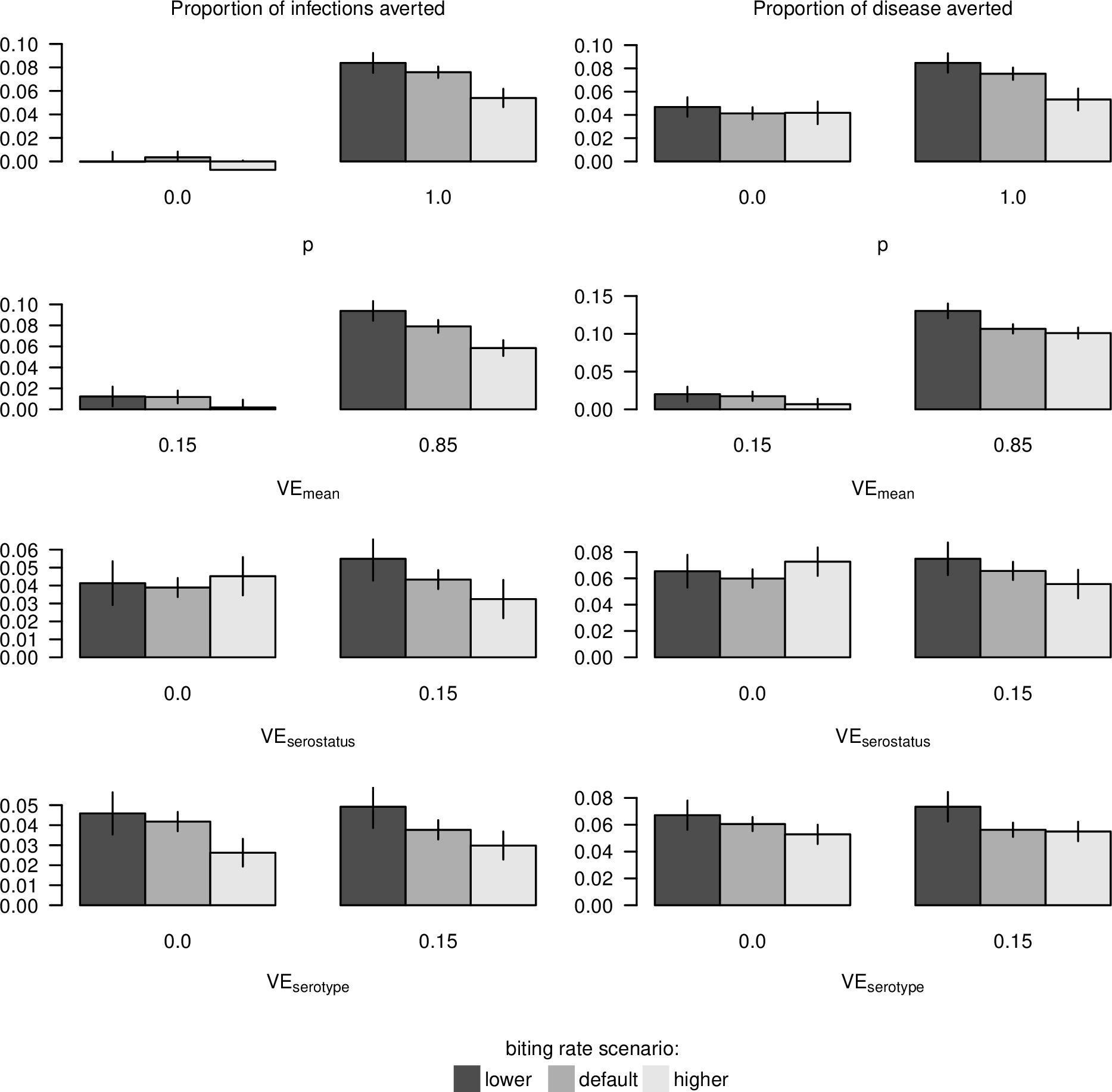

Supplement: S36 Fig — Bars display point estimates and 95% confidence intervals obtained from fitting generalized additive models to simulation results across the range of each parameter while holding others at the midpoints of their ranges. These values are comparable to the extremes displayed in Fig 9 (p, VEmean) and S23 Fig (VEserostatus, VEserotype) but under different assumptions about mosquito biting rate. (TIF) [file pcbi.1006710.s039.tif]

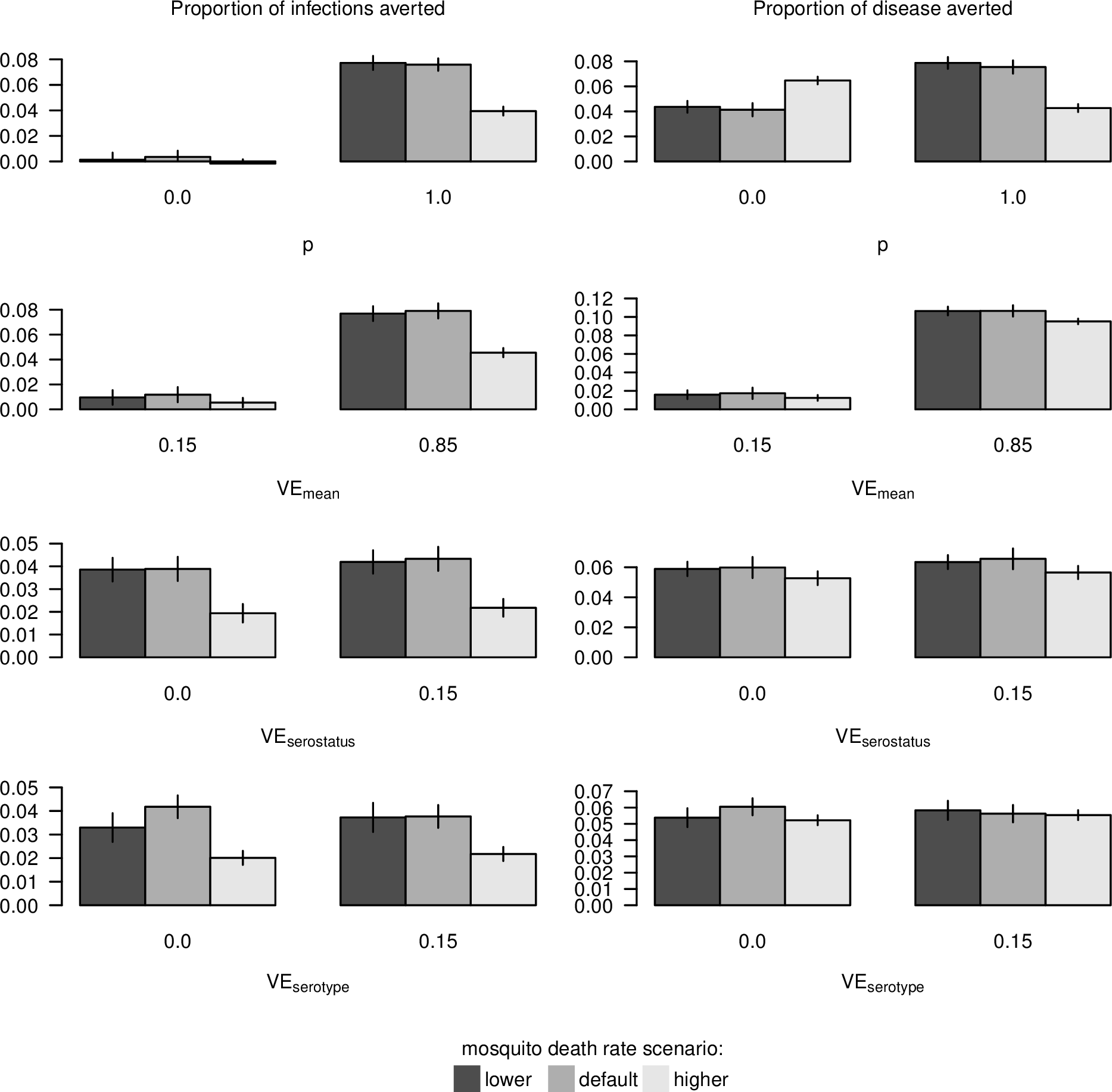

Supplement: S37 Fig — Bars display point estimates and 95% confidence intervals obtained from fitting generalized additive models to simulation results across the range of each parameter while holding others at the midpoints of their ranges. These values are comparable to the extremes displayed in Fig 9 (p, VEmean) and S23 Fig (VEserostatus, VEserotype) but under different assumptions about mosquito death rate. (TIF) [file pcbi.1006710.s040.tif]

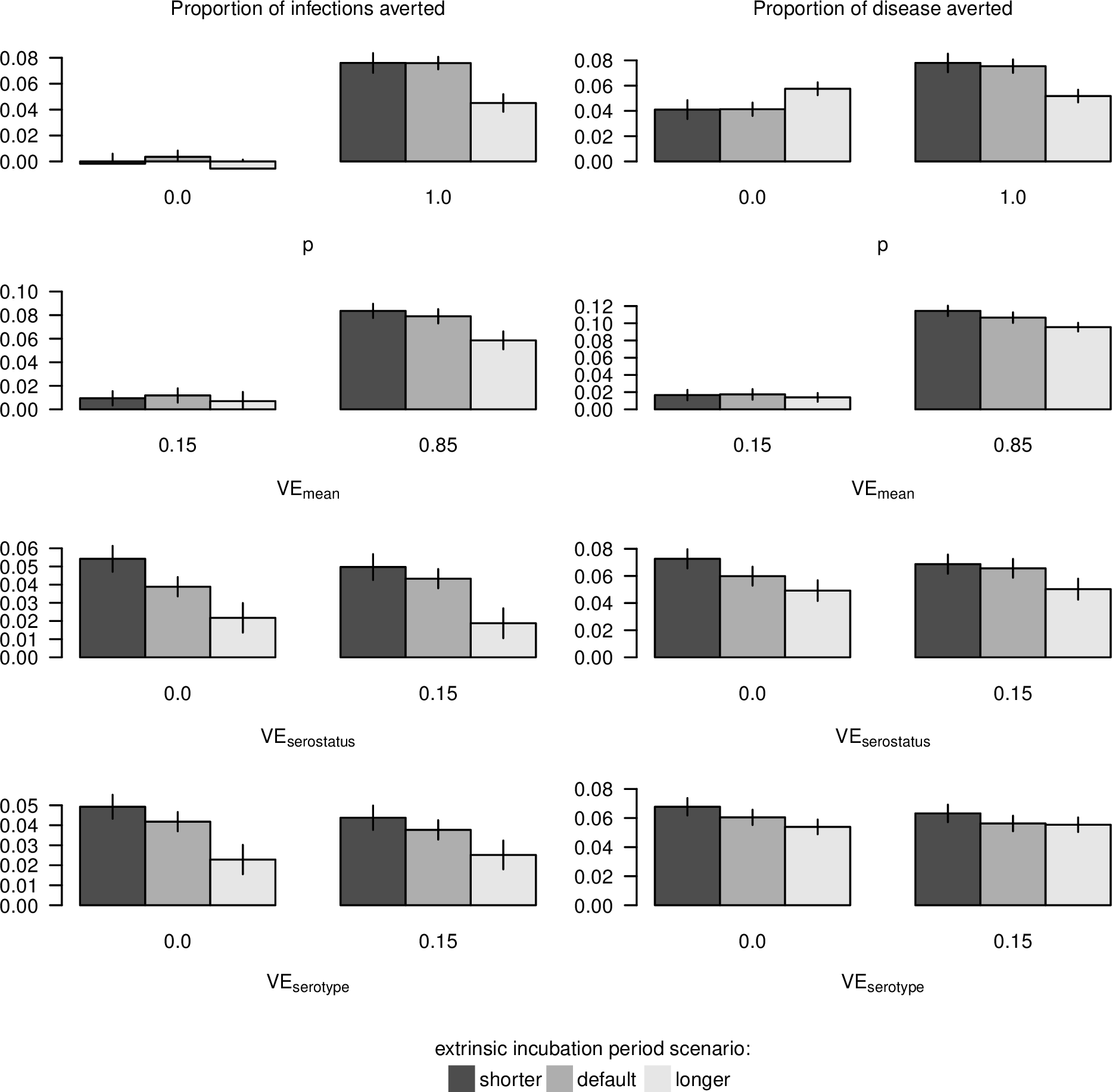

Supplement: S38 Fig — Bars display point estimates and 95% confidence intervals obtained from fitting generalized additive models to simulation results across the range of each parameter while holding others at the midpoints of their ranges. These values are comparable to the extremes displayed in Fig 9 (p, VEmean) and S23 Fig (VEserostatus, VEserotype) but under different assumptions about the extrinsic incubation period. (TIF) [file pcbi.1006710.s041.tif]

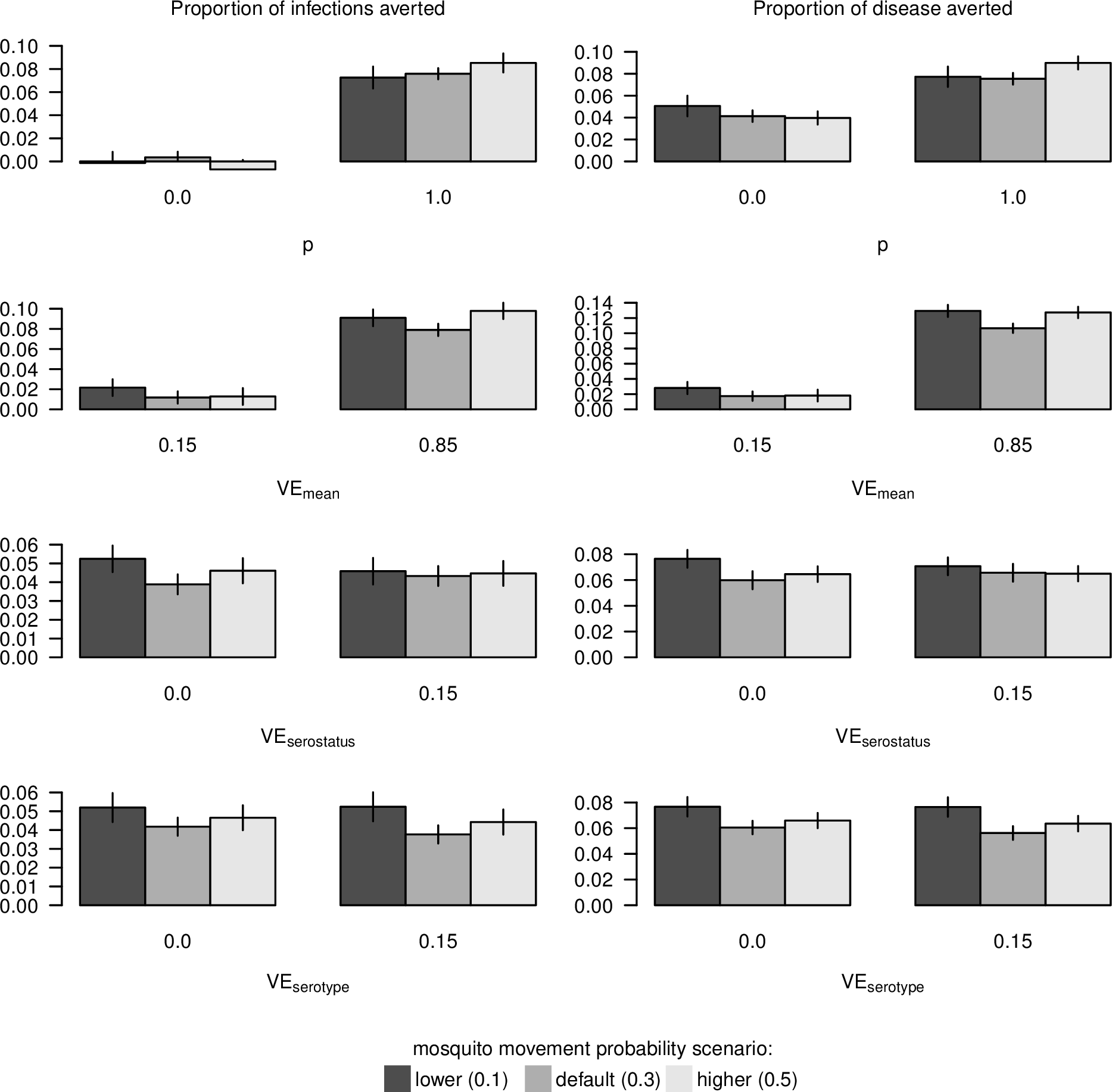

Supplement: S39 Fig — Bars display point estimates and 95% confidence intervals obtained from fitting generalized additive models to simulation results across the range of each parameter while holding others at the midpoints of their ranges. These values are comparable to the extremes displayed in Fig 9 (p, VEmean) and S23 Fig (VEserostatus, VEserotype) but under different assumptions about mosquito movement probability. (TIF) [file pcbi.1006710.s042.tif]

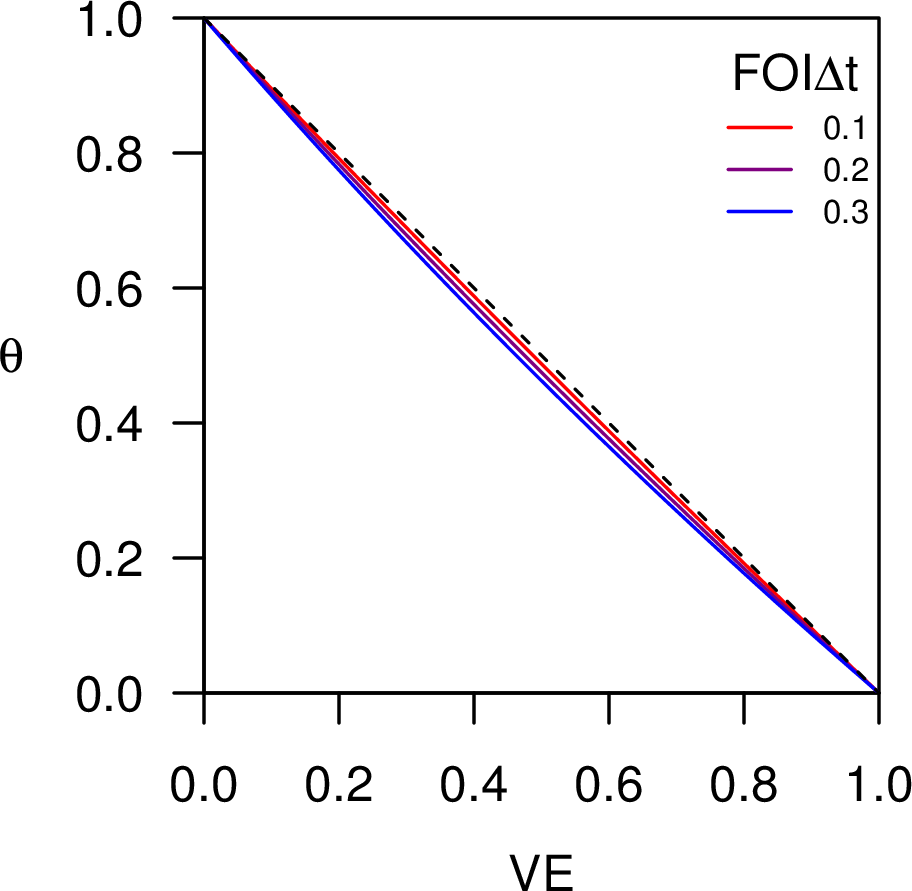

Supplement: S40 Fig — (TIF) [file pcbi.1006710.s043.tif]
